# Synthesis and preliminary evaluation of novel compounds that demonstrate broad host-directed anti-leishmanial activity

**DOI:** 10.1371/journal.pntd.0014520

**Published:** 2026-07-13

**Authors:** Elizabeth G. Gurysh, M. Shamim Hasan Zahid, Monica M. Johnson, Antonio Landavazo, Ojas A. Namjoshi, Joseph W. Wilson, Devika M. Varma, Ryan N. Woodring, Aaron T. Hendricksen, Alexandra M. Lopez, Joseph F. Vath, Baiyi Quan, Erica N. Pino, Michael C. Fitzgerald, Kevin J. Frankowski, Eric M. Bachelder, Bruce E. Blough, Kristy M. Ainslie

**Affiliations:** 1 Division of Pharmacoengineering and Molecular Pharmaceutics, Eshelman School of Pharmacy, University of North Carolina, Chapel Hill, North Carolina, United States of America; 2 Center for Drug Discovery, RTI International, Research Triangle Park, Durham, North Carolina, United States of America; 3 Department of Chemistry, Duke University, Durham, North Carolina, United States of America; 4 Center for Integrative Chemical Biology and Drug Discovery, Eshelman School of Pharmacy, University of North Carolina, Chapel Hill, North Carolina, United States of America; 5 Joint Department of Biomedical Engineering, UNC School of Medicine, University of North Carolina, Chapel Hill, North Carolina, United States of America; 6 Department of Microbiology and Immunology, UNC School of Medicine, University of North Carolina, Chapel Hill, North Carolina, United States of America; Institut Pasteur, FRANCE

## Abstract

Leishmaniasis, a neglected tropical disease affecting nearly 10% of the global population, suffers from limited therapeutic options and rising drug resistance. To address this, we developed 343 analogs of AR-12, a compound that has previously illustrated host-directed anti-leishmanial effects. Primary screening using a luminescence-based assay revealed 66 analogs with greater selectivity than the parent compound, AR-12. Sixteen promising candidates, selected for high potency (IC₅₀ < 1 µM) or high selectivity (>15), underwent secondary screening via Giemsa staining. Four lead compounds (**53**, **134**, **197**, and **354**) demonstrated therapeutic indices greater than 40. Tertiary assays confirmed their broad *in vitro* efficacy against both *Leishmania donovani* and *Leishmania mexicana*. Notably, 197 exhibited potent host-directed and proteomic analysis identified lysozyme as a mechanistic target, implicating it in the host-mediated clearance of intracellular parasites. These findings highlight the dual host- and pathogen-directed mechanisms of these compounds and support their potential as the basis for new therapeutic strategies. Further optimization and clinical exploration of these leads are warranted to meet the urgent need for effective leishmaniasis treatments.

## Author summary

Leishmaniasis is a parasitic infectious disease with limited therapeutic options and rising drug resistance. Drugs that act directly against *Leishmania* can further drive drug resistance. Host-directed therapies work to enable the host responses to enhance pathogen clearance and reduce disease progression. Host-directed therapies both limit the emergence of new drug resistance and combat drug resistant infections. In this work we screened a library of 343 AR-12 analogs for host-directed anti-leishmanial activity. This work identified four hit compounds. We then did proteomic analysis to understand the mechanism of action of the primary lead compound, identifying the protein lysozyme as having a role in host-directed activity against *Leishmania* infection.

## Introduction

Leishmaniasis, an infection caused by the parasites of the *Leishmania* genus*,* is classified by the WHO as a neglected tropical disease. Nearly 10% of the world’s population is at risk of acquiring one form of leishmaniasis. Worldwide it is estimated that there are 12 million active cases of leishmaniasis, with approximately 1 million new cases occurring each year. Among parasitic infections, this disease is responsible for the highest number of DALYs (Disability adjusted life years; a measure of health burden) after malaria. Visceral leishmaniasis (VL) is the most clinically serious form of leishmaniasis and is fatal without chemotherapeutic intervention. For the last 70 years, the most common treatment of VL is the systemic injection of sodium stibogluconate (SSG) or meglumine antimoniate formulations (antimonials). Antimonials are metalloid based (Sb), highly toxic, and have severe adverse side effects including pancreatitis and cardiac arrhythmia [1]. Additionally, many strains of *L. donovani* have become resistant to antimonial therapies, demanding the development of new chemotherapeutics for VL treatment. Miltefosine and the antifungal drug amphotericin B (AmpB) have also been used for *Leishmania* treatment. Unfortunately, miltefosine is not a preferred drug because it is teratogenic. While AmpB is highly effective in treating leishmaniasis, it is highly toxic and requires encapsulation in a liposomal formulation (AmBisome). In addition, AmpB is hypothesized to act directly on the *Leishmania* by binding to ergosterol found in the membrane of *Leishmania* [2] and preventing entry of the promastigote into the macrophage. This direct activity can drive selective pressure in the development of drug resistance towards the treatment. In fact, researchers have already isolated a strain of *Leishmania* that demonstrates resistance to AmpB [3]. It has also been observed that certain *Leishmania* strains have an inherent broad spectrum resistance against drugs they have not previously encountered, illustrating that there would be some resistance to any proposed parasite specific therapy [4].

One strategy to overcome drug resistance is through use of a host-directed therapy that improves the host cell’s ability to clear infection. This removes the direct pressure on the pathogen and can mitigate further drug resistance. Targeting the host rather than the pathogen can also potentially treat drug resistant strains [5]. Furthermore, because many pathogens take advantage of similar pathways, there is a potential for developing therapies that target a broad-spectrum of pathogens. OSU-03012, also known as AR-12, is an orally active pyruvate dehydrogenase kinase isoenzyme 1 (PDK-1) and protein kinase B (AKT) signaling pathway inhibitor that reached Phase 1 clinical trials as a potential cancer therapeutic. During development, AR-12 was found to induce autophagy, which has been shown to disrupt the life-cycle of some intracellular pathogens [6,7]. Additionally, it inhibits expression of Glucose Regulated Protein (GRP78), which has been shown to be induced in *Leishmania* infected macrophages [8]. As a host-directed therapy against VL, our work has shown that in vitro and in vivo, AR-12 can decrease the parasite burden of *L. donovani* in infected macrophages while having no direct effect on the promastigote [9]. Additionally, co-treatment with a conventional therapeutic, AmpB, resulted in a significant reduction in parasite burden compared to encapsulated OSU-03012 or AmpB alone, indicating the potential of AR-12 in sensitizing parasites to AmpB [10].

**Fig 1:**
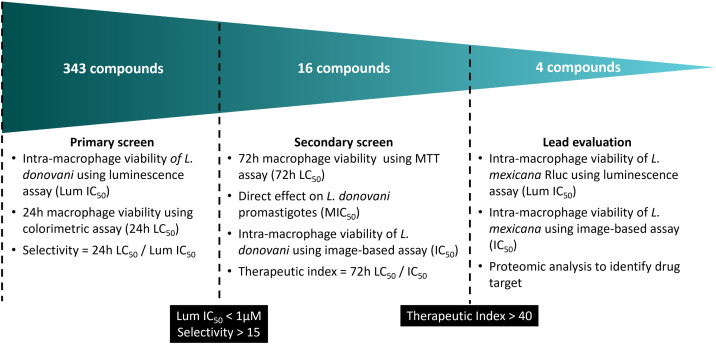
Process flow of screening methodology and lead compound selection. Concentration at which intracellular *Leishmania* burden is reduced by 50% in THP1 macrophages (Lum IC_50_) as identified by luminescence assay. Concentration where THP-1 macrophage cell viability is 50% (LC_50_) after incubation with compound as determined by MTT assay. Concentration at which intracellular *Leishmania* burden is reduced by 50% in bone marrow derived macrophages (IC_50_) as identified image-based Giemsa staining. Minimum inhibitory concentration (MIC) where extracellular *Leishmania* promastigote viability is reduced by 50% (MIC_50_) after 72-hour incubation with compound as measured by resazurin assay.

In this work, we have screened a re-purposed library of 343 compounds derived from the FDA IND approved cancer drug AR-12 for host-directed anti-leishmanial activity. Additionally, we used a medium-throughput luminescence-based screening assay for detection of intracellular *Leishmania* amastigote viability. This method was validated against more conventional image-based analysis (Giemsa stain). The culmination of this work (Fig 1) identified four novel lead compounds that are significantly improved host-directed therapies compared to parental compound AR-12. Furthermore, proteomic analysis identified a proposed mechanism of action for the most promising lead compound being linked to lysozyme and its interaction with parasite.

## Results and discussion

**Fig 2:**
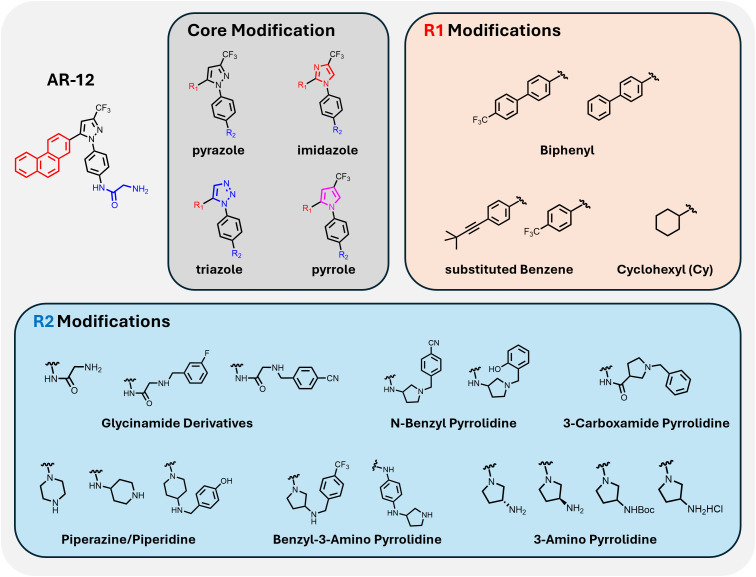
Schematic detailing workflow of structure activity relationship for chemical compounds derived from AR-12 with associated core, R1, and R2 modifications.

AR-12 (Fig 2) has been shown to have host-directed effects that decrease the parasite burden of *L. donovani* in infected macrophages [9]. AR-12 is based on an N1-aryl-3-trifluoromethyl pyrazole core structure, shown with variable R₁ (red) and R₂ (blue) positions. To explore structure–activity relationships, a focused library of 343 AR-12 analogs was synthesized with systematic substitutions at R₁ and R₂. To systematically expand the chemical space surrounding AR-12, we designed a modular library centered on a 5-trifluoromethyl-substituted pyrazole core. The CF3 group was strategically retained to preserve the lipophilicity and metabolic stability associated with the parent scaffold, while providing an electronically stabilized heterocyclic platform for regioselective diversification. Our design strategy utilized a two-vector approach to enable a comprehensive structure-activity relationship (SAR) exploration. The first is a C3-diversification (R1). This was undertaken to probe hydrophobic and steric interactions hypothesized to influence target engagement. We then introduced biphenyl substituents to increase aromatic surface area and rigidity, alongside a series of alkyl and aryl groups (e.g., cyclohexyl) to systematically vary steric bulk and conformational flexibility. The second site of diversity is the N2-Functionalization (R2). This was achieved through the installation of a 4-aminobenzyl handle which was selected as a synthetically tractable and chemically versatile intermediate that permitted late-stage derivatization while maintaining a consistent spatial orientation relative to the pyrazole core. The synthetic route was designed to be convergent and modular, allowing for the independent variation of the R1 and R2 positions. The 5-CF3 pyrazole intermediate served as a common scaffold from which sequential N-alkylation and C3 cross-coupling (or equivalent substitution chemistry) enabled the rapid generation of 343 structurally diverse analogues. This approach facilitated efficient library expansion while maintaining synthetic scalability and reproducibility across the chemical series. By varying R1 and R2, we were able to isolate the specific electronic and steric contributions of each substituent to the observed antiparasitic activity. The complete chemical structures of all compounds can be found in S1 Table. R₁ modifications included cyclohexyl, substituted benzene and biphenyl derivatives. R₂ modifications encompass a chemically diverse set of glycinamide derivatives, 3-amino pyrrolidine, piperazine, N-benzyl pyrrolidine, and other elaborations on isosteres for the carboxyamide group of AR-12 (Fig 2).

### Primary screen

Primary screening of the 343 compounds was performed by medium throughput luminescence-based screening assay using THP-1 macrophages and luminescent *L. donovani* (S1 Fig, S2 Table). Additionally, the effect of the compounds on host cell viability was evaluated in uninfected THP-1 cells using a colorimetric assay. These two values were used to calculate selectivity of the compounds (24hr LC_50_/ Lum IC_50_). Comparing these compounds to AR-12 potency and toxicity to the host cell revealed 33 compounds both less cytotoxic and more potent (S2 Fig). These hits outline clear structure–activity trends. Biphenyl- and cyclohexyl-substituted pyrazoles emerged as favored scaffolds and select imidazole-core variants validated the conserved behavior of these motifs. Within R₂ substitutions, glycinamide derivatives (**50**, **158**, **197**, **354**, and **355**) consistently enhanced selectivity, while 3-amino pyrrolidines, particularly those decorated with polar benzyl groups (**53**, **91**, **356**, **362**) increased potency, though sometimes at the expense of host tolerability. Importantly, modification of the pyrrolidine amine by protection or ionization (**133**, **134**, **281**) yielded solubility and selectivity advantages, suggesting an accessible synthetic handle within this series. Cyclohexyl R₁ analogs paired with piperidine or piperazine derivatives (**324**, **334**, **336**, **339**, **370**, **416**, **417, 418, 419, 420, 421**) extended the structure-activity landscape, with alkoxypiperidines preserving activity while maintaining host viability. Changing the core-scaffold to imidazole series reinforced these trends, with compounds **154**, **158**, and **197** retaining potent intracellular activity. From these observations, sixteen compounds (Table 1, S4 and S5 Figs) were identified with either a high potency (Lum IC_50_ < 1 µM) or a high selectivity (>15) and were selected for secondary screening.

**Table 1:** Hits identified by primary screen. Hits determined by potent host-directed activity (IC_50_ < 1 µM) or high selectivity (>15). Lum IC_50_ is the concentration at which intracellular *L. donovani* burden is reduced by 50% in THP1 macrophages as identified by luminescence assay. Concentration where THP-1 macrophage cell viability is 50% (LC_50_) after 24-hour incubation with compound as determined by MTT assay. Selectivity between host-directed effect and cytotoxicity, defined as 24h LC_50_/ Lum IC_50_. Parental compound AR-12 provided for reference.

	24 hr viability of THP-1 macrophages	Intracellular *L. donovani* amastigotes	Selectivity
Compounds	24h LC_50_ (µM)	Lum IC_50_ (µM)	24h LC_50_/ Lum IC_50_
**AR-12**	13.3	3.4	3.9
**23**	5.8	0.4	14.8
**25**	28.9	0.7	43.8
**44**	7.4	0.9	8.1
**50**	23.5	1.5	16.0
**53**	>50	0.4	>112.6
**86**	21.9	1.4	16.0
**91**	>50	1.4	>36.1
**129**	6.3	0.1	46.7
**130**	4.2	0.2	23.9
**133**	29.0	0.9	32.5
**134**	22.6	0.5	42.1
**158**	16.0	0.2	81.0
**197**	>50	2.4	>20.6
**319**	6.4	0.8	8.1
**354**	22.0	0.5	44.9
**408**	25.4	1.5	17.0

### Secondary screen

Additional screening of the selected 16 compounds was performed to confirm drug activity. Specifically, the effect of drugs on macrophage viability was evaluated over a longer range of time (72 hrs, S6 Fig). Furthermore, the direct effect of the compounds on extracellular *L. donovani* promastigotes was measured using resazurin assay (S7 Fig). Lastly, activity of compounds to reduce intracellular *L. donovani* was confirmed using Giemsa staining and image-based analysis in BMDMs was assessed (S8 Fig). The host-directed therapeutic index was calculated by the 72 hr LC_50_/ IC_50_. This characterization is detailed in Table 2 for all 16 compounds. Structure-activity analysis of the 16 candidates converged on four leads (**53**, **134**, **197**, and **354**) that achieved therapeutic indices above 40. A strong preference for biphenyl substitution at R₁ was evident, appearing in three of the four leads, emphasizing its role as a privileged motif for potency and selectivity. Compound **53** exemplified how polar, electron-withdrawing groups can improve intracellular efficacy. In contrast, compound **197** demonstrated the successful transfer of these structural features to a new heteroaryl core. Cyclohexyl analog **354** confirmed that glycinamide R₂ groups can pair productively with alternative hydrophobic motifs, yielding a profile of balanced potency and host safety. Taken together, these results illustrate that optimal host-directed activity is achieved when hydrophobic R₁ scaffolds are complemented by solubilizing, electronically altered R₂ functionalities. These four leads were prioritized for tertiary screening in cutaneous strain, *L. mexicana,* to ensure broad host-directed activity.

**Table 2:** Further analysis of hit compounds. Concentration at which intracellular *L. donovani* burden is reduced by 50% in bone marrow derived macrophages (IC_50_) as identified image-based Giemsa staining. Minimum inhibitory concentration (MIC) where extracellular *L. donovani* promastigote viability is reduced by 50% (MIC_50_) after 72-hour incubation with compound as measured by resazurin assay. Concentration where THP-1 macrophage cell viability is 50% (LC_50_) after 72-hour incubation with compound as determined by MTT assay. Therapeutic index between host-directed effect and cytotoxicity, defined as LC_50_/IC_50_. Parental compound AR-12 provided for reference.

	Extracellular *L. donovani* promastigotes	Intracellular *L. donovani* amastigotes	72 hr viability of THP-1 macrophages	Therapeutic Index
Compounds	MIC_50_ (µM)	IC_50_ (µM)	LC_50_ (µM)	LC_50_/ IC_50_
**AR-12**	2.2	2.2	8.6	4
**23**	4.0	0.9	6.9	8
**25**	2.1	2.5	6.3	2
**44**	1.9	1.0	14.5	14
**50**	14.6	9.1	42.7	5
**53**	>200	1.1	>300	>273
**86**	3.2	0.6	8.2	14
**91**	56.3	2.4	53.4	22
**129**	1.7	0.4	6.2	17
**130**	2.1	1.6	6.4	4
**133**	13.0	1.7	33.9	20
**134**	5.7	0.7	32.0	46
**158**	4.6	0.6	16.5	29
**197**	6.4	1.1	>300	>273
**319**	0.8	3.4	7.2	2
**354**	22.1	0.4	20.2	51
**408**	0.9	2.6	25.1	10

### Tertiary screen

**Fig 3:**
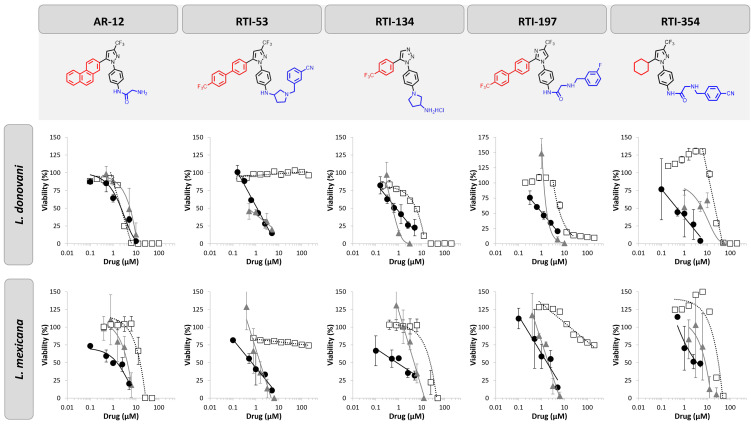
Lead compounds. Dose response of intracellular *Leishmania* burden in THP-1 macrophages as identified by luminescence-based screen (gray triangle). Dose response of intracellular *Leishmania* burden in bone marrow derived macrophages as identified image-based Giemsa staining (black circle). Dose response of extracellular *Leishmania* promastigote viability after 72-hour incubation with compound as measured by resazurin assay (open square). Parental compound AR-12 provided for reference. Data is presented as mean ± standard deviation of biological triplicates.

The four lead compounds were then screened in *L. mexicana* using the same assays described for *L. donovani*. The ability of compounds to reduce intracellular *Leishmania* was evaluated by both luminescent and image-based assays. Additionally, the direct activity of compounds on extracellular *L. mexicana* promastigotes was measured using resazurin assay (Fig 3, Table 3). The ability of these four compounds to reduce intracellular burden was similar for both *L. mexicana* and *L. donovani* as measured by luminescent and image-based assays. However, the direct effect of compounds on promastigotes varied between the visceral and cutaneous strains. This suggests that the host-directed effect is the driving factor in intracellular pathogen clearance and is less susceptible to differences in *Leishmania* strains.

**Table 3:** Comparison across cutaneous and visceral *Leishmania* strains. Concentration where THP-1 macrophage cell viability was 50% (LC_50_) after 72-hour incubation with compound as determined by MTT assay. Concentration at which intracellular *L. donovani* or *L. mexicana* burden was reduced by 50% in bone marrow derived macrophages (IC_50_) as identified image-based Giemsa staining. Minimum inhibitory concentration (MIC) where extracellular *L. donovani* or *L. mexicana* promastigote viability was reduced by 50% (MIC_50_) after 72-hour incubation with compound as measured by resazurin assay. Therapeutic index between host-directed effect and cytotoxicity, defined as LC_50_/IC_50_. Parental compound AR-12 provided for reference.

		*L. donovani*			*L. mexicana*		
	Viability of THP-1 macrophages	Extracellular promastigotes	Intracellular amastigotes	Therapeutic Index	Extracellular promastigotes	Intracellular amastigotes	Therapeutic Index
**Cmpd**	**LC**_**50**_ **(µM)**	**MIC**_**50**_ **(µM)**	**IC**_**50**_ **(µM)**	**LC**_**50**_ **/ IC**_**50**_	**MIC**_**50**_ **(µM)**	**IC**_**50**_ **(µM)**	**LC**_**50**_ **/ IC**_**50**_
**AR-12**	8.6	2.2	2.2	4	15.1	1.5	6
**53**	>300	>200	1.1	>273	>200	0.7	>428
**134**	32.0	5.7	0.7	46	16.5	0.8	40
**197**	>300	6.4	1.1	>273	>200	1.7	>176
**354**	20.2	22.1	0.4	51	30.7	3.5	6

Tertiary screening revealed two compounds of interest: **53** and **197**. Neither compound had any reduction in macrophage viability over a 72 hr incubation up to 300µM (Table 3). However, both were able to decrease intracellular parasite burden by 50% with less than 2 µM in both visceral and cutaneous *Leishmania* strains (Table 3, Fig 3). Interestingly, **53** had no direct effects on extracellular promastigote viability indicating that all anti-leishmanial effects are host-directed. While **197** does have some direct reduction in *L. donovani* extracellular promastigote viability, the host-directed effects are five-fold more significant and notably, **197** had no effect on *L. mexicana* extracellular promastigote viability.

### 197 proteomic analysis and target validation

Although the highest selectivity was noted with **53** in two strains, **197** displayed broad activity against the promastigote as well as host-directed activity. Furthermore, the synthetic strategy required to introduce a chemical handle onto **197** for affinity-capture studies was more approachable than for **53**. Therefore, **197** was selected for subsequent proteomic analyses. For these reasons we utilized two different proteomic strategies to identify the molecular target of **197**. In one strategy, **197** was chemically modified (S9A Fig) and conjugated to agarose beads using a 5-carbon spacer (termed ‘197-bead’). Chemical modification did not significantly affect 197 anti-*Leishmania* activity (S9B Fig). We also prepared a bead conjugated to butylamine to serve as a control for nonspecific interactions (termed ‘control-bead’). The beads were then incubated with *Leishmania* infected macrophage lysate. Afterwards, the beads were washed and boiled in sample buffer to release bound proteins which were then evaluated by LC-MS/MS. This analysis identified 841 human and 65 *Leishmania* enriched proteins on the 197-beads compared to control beads. These enriched proteins all had a Log2 fold-change >1 and p < 0.05 (S10A Fig**)**.

In a second strategy, the thermal proteome profiling (TPP) approach was used to identify the proteins in *Leishmania* infected macrophage lysate that displayed a **197**-induced change in thermal stability. The TPP approach is an attractive method by which to identify the direct (and indirect) targets of protein-ligands including small molecule drugs. The TPP experiment performed here effectively assayed over 1600 human proteins and over 1000 Leishmania proteins for binding to **197**. A total of 123 human and 71 *Leishmania* proteins were identified with **197**-induced changes in their thermal stability using selection criteria analogous to that used in the affinity pull-down experiment (i.e., a |z-score| > 1 and a p < 0.05) (S10B Fig).

**Fig 4:**
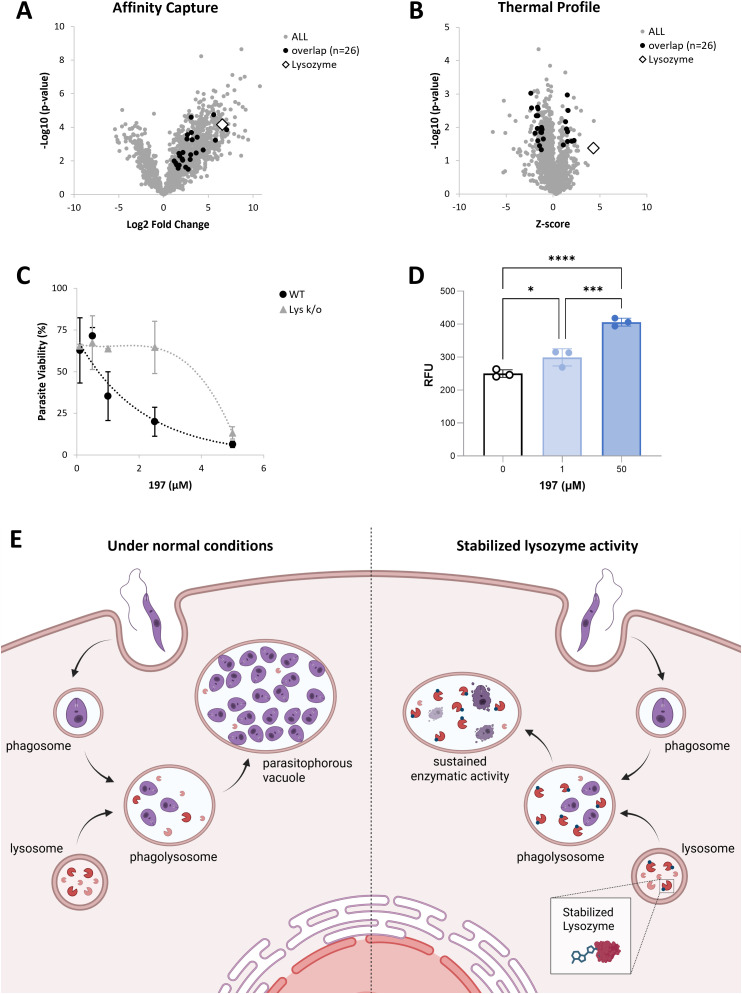
Proteomic Analysis. **A)** All human proteins (gray circle) identified by affinity capture using **197** functionalized bead plotted Log2(fold-change) and -Log10(p-value) over control bead. Proteins with a Log2FC > 1 and p < 0.05 overlapping with significant proteins identified by thermal profile analysis (|z-score| > 1 and p < 0.05) are shown with black circles. Lysozyme protein identified by white diamond. **B)** All human proteins (gray circle) identified by thermal profile analysis with **197**. Proteins with a (|z-score| > 1 and p < 0.05 overlapping with significant proteins identified by affinity capture (Log2FC > 2 and p < 0.05) are shown with black circles. Lysozyme protein identified by white diamond. **C**) Dose response of **197** on intracellular *Leishmania* burden in bone marrow derived macrophages derived from wildtype C57BL/6 (black circle) or lysozyme knockout mice (gray triangle) as identified image-based Giemsa staining. Data is presented as mean ± standard deviation of biological triplicates. **D)** Fluorometric lysozyme activity of recombinant human lysozyme protein (1mg/mL) with increased **197** concentration (0, 1, and 50 µM). *p < 0.05, *** < 0.001, **** p < 0.001 by one-way ANOVA with Tukey’s multiple comparisons test. **E)** Schematic of proposed mechanism. Under normal conditions (left), *Leishmania* promastigote is internalized into a phagosome which matures into a phagolysosome by fusing with a lysosome. *Leishmania* amastigotes interfere with maturation, remodeling the phagolysosome into a parasitophorous vacuole permissive for parasite replication. With host-lysozyme stabilized by **197** (right), phagolysosome maturation proceeds and enzymatic activity is maintained allowing host cell degradation of amastigotes. Created in BioRender. Gurysh, **E.** (2026) https://BioRender.com/r0k0zjq.

Cross-referencing the 841 significant human proteins from the affinity capture and 123 human proteins from TPP analysis revealed 26 overlapping proteins (Fig 4A and 4B). Further evaluating strength of interaction, refining affinity capture proteins to Log2 fold-change > 4 and TPP proteins to |z-score| > 4 revealed one protein: lysozyme (Fig 4A and 4B). The overlap between the 65 and 71 *Leishmania* proteins identified in affinity and TPP analysis, respectively, was also investigated revealing three overlapping proteins (S10C and S10D Fig). Further evaluating strength of interaction, refining affinity capture proteins to Log2 fold-change > 4 and TPP proteins to |z-score| > 4 revealed 0 proteins (S10E Fig). These results further support the idea that **197** is acting in a host-directed manner.

To evaluate lysozyme as a mechanistic target for **197**, the ability of **197** to eradicate parasitic burden from BMDM cells derived from both wild-type (WT) and lysozyme knockout (Lys K/O) mice were evaluated. Amphotericin B, which has a different mechanism of action, acting on membrane sterols to decrease permeability barrier to small metabolites, was utilized as a control [11]. Amphotericin B retained its anti-leishmanial activity in both WT and Lys K/O BMDMs (S11 Fig); however, **197** activity was notably decreased in Lys K/O BMDMs (Fig 4C). Specifically, the IC_50_ for 197 in Lys K/O BMDMs was more than five-fold greater than in WT BMDMs. This supports proteomic analysis that lysozyme is a target of 197 and plays a role in host-directed anti-*Leishmania* effect.

To further assess the biological relevance of lysozyme activation by **197**, lysozyme activity was measured using a commercially available assay kit. Treatment with 197 resulted in a dose-dependent increase in lysozyme activity (Fig 4D), consistent with the proteomic findings and the reduced efficacy observed in lysozyme-deficient macrophages. This data provides additional evidence supporting lysozyme as a mechanistic target of **197**.

Lysozyme is a ~ 14 kDa enzyme present in mucosal secretions and tissues of animals. It is also present in cytoplasmic granules of macrophages and the polymorphonuclear neutrophils and plays role in innate immunity. Lysozyme catalyzes the hydrolysis of 1,4-beta-linkages in peptidoglycan, which is a major component of gram-positive bacterial cell wall, resulting in bacterial lysis. Lysozyme has recently been explored as an alternative to antibiotics [12]. While lysozyme is primarily known for its antibacterial properties, Valigurova et al. has shown that lysosomal endocytosis plays a role in *Leishmania* host cell infection [13]. Most notably, Kumar et al. concluded that low lysozyme activity in patients may account for persistence of *Leishmania* parasites in VL infections [14].

Under normal conditions, the *Leishmania* promastigote is internalized into a phagosome which matures into a phagolysosome by fusing with a lysosome. *Leishmania* amastigotes interfere with phagolysosome maturation, remodeling it into a parasitophorous vacuole permissive for parasite replication. TPP analysis of **197** had a strongly positive z-score of 4.32 for lysozyme. A positive z-score typically indicates increased protein stability often due to interactions such as ligand binding, post-translational modifications, or complex formation [15,16]. This TPP result is consistent with the affinity capture finding of an increase in abundance of lysozyme (Log2 fold change = 6.57). These results both suggest that **197** helps stabilize the enzyme lysozyme. Mechanistically, this host-modification could help reduce intracellular infection by preventing *Leishmania*-induced remodeling of the phagolysosome into a parasitophorous vacuole. This prevents the formation of the permissive niche for parasitic replication and allows destruction of internalized amastigotes by the host cell (Fig 4E).

This study has several limitations. First, all experiments were conducted in vitro. Future studies evaluating compound **197** in relevant animal models of leishmaniasis will be important to determine translational potential. Additionally, while the proteomic analyses and biologic assays identify lysozyme as a mechanistically relevant target, direct evidence of protein–ligand binding has not yet been measured. Biophysical approaches will be necessary to confirm target engagement and further define the interaction between lysozyme and compound **197**. Extending these studies to the other three lead analogs, as well as the parent compound AR-12, may provide additional insight into the structural determinants underlying this host-directed anti-leishmanial activity.

Overall, this work advances our understanding of the molecular mechanisms underlying anti-leishmanial activity of our analogs and confirms host-directed activity. Additionally, it paves the way for the development of novel therapeutic strategies to combat this pervasive disease. Future research should focus on optimizing these lead compounds and exploring their potential in clinical settings to address the urgent need for effective leishmaniasis treatments.

## Methods and materials

### Synthetic methods for hit compounds

Below is the synthesis for the 16 hit compounds, a more complete methods and characterization can be found at the end of the S1 File.

**23:** A solution of **7** (150 mg, 0.29 mmol), 4-nitrobenzaldehyde (44 mg, 0.29 mmol) and 4A molecular sieves (150 mg) in anhydrous methanol (3 mL) and anhydrous tetrahydrofuran (1.5 mL) was stirred at room temperature for 18 hours. The reaction was cooled to 0^o^ C and treated with sodium borohydride (22 mg, 0.58 mmol) was added and the reaction stirred for 4 hours at room temperature. The reaction was concentrated and the residue partitioned between saturated aqueous sodium bicarbonate solution and ethyl acetate. The combined organic layers were washed with brine, dried (Na_2_SO_4_), filtered and concentrated to yield 203.6 mg of a brown gel. The crude material was purified over silica gel using 0–10% methanol from dichloromethane to yield 65 mg (36%) of **23** as an off-white solid. ^1^H-NMR (CDCl_3_) δ 7.69 (s, 4 H), 7.56 (d, 2 H, J = 9 Hz), 7.34 (d, 2 H, J = 6 Hz), 7.14 (dd, 4 H, J = 3 Hz, 9 Hz), 6.77 (s, 1 H), 6.66 (d, 2 H, J = 6 Hz), 6.47 (d, 2 H, J = 9 Hz), 3.76 (s, 2 H), 3.63-3.41 (m, 6 H), 3.39-3.29 (m, 1 H), 3.19-3.08 (m, 1 H), 2.31-2.19 (m, 1 H), 1.99-1.88 (m, 1 H). ESI-MS, calculated for C_34_H_27_F_6_N_5_O_2_ (MH)^+^ 622.6; observed 622.3.

**25:** A solution of **15** (4.90 g, 7.96 mmol) in dichloromethane (55 mL) was cooled to 0^o^ C and treated with trifluoroacetic acid (5.9 mL, 79.4 mmol). The reaction warmed to room temperature and stirred for 18 hours. Upon completion, the mixture was concentrated and the residue was partitioned between ethyl acetate and 2 N aqueous NaOH. The aqueous layer was extracted with ethyl acetate and the combined organic layers were washed with brine, dried (Na_2_SO_4_), filtered and concentrated to yield a brown solid (**25**, 4.0 g, 97%) that required no further purification. ^1^H-NMR (CDCl_3_) δ 7.68 (dd, 4 H, J = 9 Hz), 7.56 (d, 2 H, J = 9 Hz), 7.36 (d, 2 H, J = 9 Hz), 7.14 (d, 2 H, J = 9 Hz), 6.78 (s, 1 H), 6.56 (d, 2 H, J = 9 Hz), 4.05-3.91 (m, 2 H), 3.21-3.08 (m, 2 H), 3.01-2.93 (m, 1 H), 2.91-2.85 (m, 1 H), 2.27-2.15 (m, 1 H). LC-MS, calculated for C_27_H_22_F_6_N_4_ (MH)^+^ 517.4; observed 517.2. Anal. Calculated (with 0.8 mol of water) for C_27_H_22_F_6_N_4_; C, 61.08; H, 4.48; N, 10.55. Found: C, 61.23; H, 4.30; N, 10.43.

**44:** A solution of ***int-28*** (34 mg, 0.061 mmol) in dichloromethane (2 mL) was cooled to 0^o^ C and treated with trifluoroacetic acid (0.2 mL, 2.69 mmol). The reaction warmed to room temperature and stirred for 15 hours. Upon completion, the mixture was concentrated and the residue was partitioned between ethyl acetate and 2 N aqueous NaOH. The aqueous layer was extracted with ethyl acetate and the combined organic layers were washed with brine, dried (Na_2_SO_4_), filtered and concentrated to yield 281 mg of **44** (>100%) of a yellow gel, which required no further purification. ^1^H-NMR (CD_3_OD) 7.67-7.61 (m, 2 H), 7.58-7.52 (m, 2 H), 7.30-7.17 (m, 4 H), 3.49-3.43 (m, 4 H), 3.37-3.32 (m, 4 H), 1.27 (s, 9 H). LC-MS, calculated for C_26_H_27_F_3_N_4_ (MH)^+^ 452.5; observed 453.2.

**50:** Following the procedures for preparing **77** and **79**, **50** was isolated as an off-white solid (264 mg, 79% for the final step). ^1^H-NMR (CDCl_3_) δ 9.66 (br s, 1 H), 7.74 (d, 2 H, J = 9 Hz), 7.57 (d, 2 H, J = 9 Hz), 7.50 (dd, 4 H, J = 9 Hz), 7.43 (dd, 2 H, J = 6 Hz, 9 Hz), 7.36 (d, 1 H, J = 9 Hz), 7.32-7.24 (m, 3 H), 3.52 (s, 2 H). ESI-MS, calculated for C_24_H_19_F_3_N_4_O (MH)^+^ 437.4; observed 437.0. Anal. Calculated (with 0.2 mol water) for C_24_H_19_F_3_N_4_O; C, 65.50; H, 4.44; N, 12.73. Found: C, 66.04; H, 4.38; N, 12.83.

**86:** A mixture containing ***int-11*** (400 mg, 1.05 mmol), 1-(Phenylmethyl)-3-pyrrolidinecarboxylic acid (325 mg, 1.58 mmol), diisopropylethylamine (0.65 mL, 3.73 mmol) and Propylphosphonic anhydride solution, 50 wt. % in ethyl acetate (1.9 mL, 3.19 mmol) in anhydrous tetrahydrofuran (38 mL) was sealed tightly and stirred at room temperature for 18 hours. The solvent was concentrated to 20% volume and the residue was partitioned between ethyl acetate and saturated aqueous NaHCO_3_. The organic layer was washed with brine, dried (Na_2_SO_4_), filtered and concentrated. The crude material was adsorbed onto silica gel and purified via ISCO using 0–10% methanol from dichloromethane to yield 513 mg (**86**%) of an off-white solid (**86**). ^1^H-NMR (CDCl_3_) δ 9.77 (s, 1 H), 7.57 (dd, 4 H, J = 6 Hz, 9 Hz), 7.48 (dd, 6 H, J = 6 Hz), 7.42 (d, 1 H, J = 6 Hz), 7.37-7.32 (m, 5 H), 7.29-7.20 (m, 4 H), 3.73 (dd, 2 H, J = 6 Hz, 12 Hz), 3.16 (dd, 2 H, J = 6 Hz, 9 Hz), 2.95 (dd, 1 H, J = 6 Hz, 9 Hz), 2.42-2.33 (m, 3 H), 2.12-2.05 (m, 1 H). LC-MS, calculated for C_34_H_29_F_3_N_4_O (MH)^+^ 567.6; observed 567.2. Anal. Calculated for C_34_H_29_F_3_N_4_O; C, 72.07; H, 5.15; N, 9.88. Found: C, 71.97; H, 5.25; N, 9.82.

**53 [R = CH**_**2**_**-(3-cyanophenyl)]:** Using 3-cyanobenzaldehyde, the product was isolated as a white solid in 58% yield (142 mg). ^1^H-NMR (CDCl_3_) δ 7.65 (dd, 5 H, J = 12 Hz), 7.55 (d, 2 H, J = 9 Hz), 7.43 (m, d, 1 H, J = 6 Hz), 7.35 (dd, 2 H, J = 9 Hz), 7.14 (dd, 2 H, J = 9 Hz), 6.77 (s, 1 H), 6.55 (d, 2 H, J = 9 Hz), 4.16-4.03 (m, 2 H), 3.65 (s, 2 H), 2.84-2.71 (m, 2 H), 2.59 (dd, 1 H, J = 9 Hz), 2.46-2.28 (m, 2 H), 1.76-1.65 (m, 1 H). ^13^C-NMR (CDCl_3_, 75 MHz) δ 147.6, 143.7, 140.4, 139.9, 133.0, 132.2, 130.9, 129.5, 129.2, 127.4, 126.9, 125.9, 118.9, 113.3, 112.5, 105.0, 60.8, 59.3, 52.8, 52.4, 32.6. ESI-MS, calculated for C_35_H_27_F_6_N_5_ (MH)^+^ 632.6; observed 632.6.

**91 [R = CH**_**2**_**-(2-hydroxyphenyl)]:** Using 2-hydroxybenzaldehyde, the product was isolated as an off-white solid in 48% yield (138.6 mg). ^1^H-NMR (CDCl_3_) δ 7.68 (dd, 4 H, J = 9 Hz), 7.56 (d, 2 H, J = 9 Hz), 7.35 (dd, 2 H, J = 9 Hz), 7.16 (dd, 3 H, J = 6 Hz, 9 Hz), 6.99 (d, 1 H, J = 9 Hz), 6.84-6.76 (m, 3 H), 6.53 (d, 2 H, J = 9 Hz), 4.06-3.97 (m, 2 H), 3.84 (s, 2 H), 2.97-2.85 (m, 2 H), 2.70 (dd, 1 H, J = 3 Hz, 6 Hz), 2.56 (dd, 1 H, J = 9 Hz), 2.42 (dd, 1 H, J = 6 Hz, 9 Hz), 1.78-1.72 (m, 1 H). ESI-MS, calculated for C_34_H_28_F_6_N_4_O (MH)^+^ 623.6; observed 623.8.

**129 (5b: R-amino orientation):** The product was isolated as a brown gel in 59% yield (160 mg). ^1^H-NMR (CDCl_3_) δ 7.69 (s, 4 H), 7.56 (d, 2 H, J = 9 Hz), 7.35 (d, 2 H, J = 9 Hz), 7.17 (d, 2 H, J = 9 Hz), 6.78 (s, 1 H), 6.49 (d, 2 H, J = 9 Hz), 3.76 (dd, 1 H, J = 3 Hz, 6 Hz), 3.55-3.45 (m, 2 H), 3.38-3.30 (m, 1 H), 3.04 (dd, 1 H, J = 3 Hz, 6 Hz), 2.29-2.18 (m, 1 H), 1.88-1.78 (m, 1 H). ^13^C-NMR (CDCl_3_, 75 MHz) δ 147.7, 143.7, 139.7, 129.2, 127.4, 127.3, 126.7, 125.9, 111.4, 104.9, 56.4, 51.4, 46.3, 34.9. ESI-MS, calculated for C_27_H_22_F_6_N_4_ (MH)^+^ 517.4; observed 517.6; Anal. Calculated for C_27_H_22_F_6_N_4_; C, 62.79; H, 4.29; N, 10.84. Found: C, 62.69; H, 4.37; N, 10.55; [α] = + 4.28 (c = 0.70/CHCl_3_).

**130 (5c: S-amino orientation):** The product was isolated as a tan solid in 82% yield (335 mg). ^1^H-NMR (CDCl_3_) δ 7.66 (s, 4 H), 7.56 (d, 2 H, J = 9 Hz), 7.35 (d, 2 H, J = 9 Hz), 7.17 (d, 2 H, J = 9 Hz), 6.78 (s, 1 H), 6.49 (d, 2 H, J = 9 Hz), 3.75 (dd, 1 H, J = 6 Hz), 3.55-3.45 (m, 2 H), 3.38-3.30 (dd, 1 H, J = 6 Hz, 9 Hz), 3.04 (dd, 1 H, J = 3 Hz, 6 Hz), 2.29-2.18 (m, 1 H), 1.88-1.78 (m, 1 H). ESI-MS, calculated for C_27_H_22_F_6_N_4_ (MH)^+^ 517.4; observed 517.6; Anal. Calculated (with 0.2 mol water) for C_27_H_22_F_6_N_4_; C, 62.35; H, 4.34; N, 10.77. Found: C, 62.16; H, 4.37; N, 10.65; [α] = - 2.50 (c = 0.80/CHCl_3_).

**133:** The following were combined in a heavy-duty glass reactor: ***int-20*** (110 mg, 0.3 mmol), 3-N-Boc-aminopyrrolidine (72.4 mg, 0.39 mmol), BINAP (56 mg, 0.09 mmol), Pd_2_(dba)_3_ (27.5 mg, 0.03 mmol) and Cs_2_CO_3_ (127 mg, 0.39 mmol) in anhydrous toluene (3 mL) and nitrogen gas was bubbled into the mixture for two minutes. The reactor was then sealed with a Teflon cap and heated to 110^o^ C for 15 hours. Upon cooling, the mixture was filtered through Celite and the filter pad was rinsed with ethyl acetate. The filtrate was washed with water and brine, dried (Na_2_SO_4_), filtered and concentrated. The crude material was purified over silica gel using 0–100% ethyl acetate from hexanes to yield 158.5 mg (>100%) of a yellow solid (**133**). ^1^H-NMR (CDCl_3_) δ 7.89 (s, 1 H), 7.59 (d, 2 H, J = 7.6 Hz), 7.37 (d, 2 H, J = 8.4 Hz), 7.15 (d, 2 H, J = 8.8 Hz), 6.53 (d, 2 H, J = 8.8 Hz), 4.74-4.68 (m, 1 H), 4.44-4.36 (m, 1 H), 3.66-3.60 (m, 1 H), 3.47-3.42 (m, 1 H), 3.41-3.34 (m, 1 H), 3.21-3.16 (m, 1 H), 2.36-2.17 (m, 1 H), 2.04-1.97 (m, 1 H), 1.44 (s, 9 H). LC-MS, calculated for C_24_H_26_F_3_N_5_O_2_ (MH)^+^ 473.5; observed 474.2.

**134:** A solution of **133** (158.5 mg, 0.334 mmol) in dichloromethane (20 mL) was cooled to 0^o^ C and treated with trifluoroacetic acid (0.5 mL, 6.73 mmol). The reaction warmed to room temperature and stirred for 15 hours. Upon completion, the mixture was concentrated and the residue was partitioned between ethyl acetate and 2 N aqueous NaOH. The aqueous layer was extracted with ethyl acetate and the combined organic layers were washed with brine, dried (Na_2_SO_4_), filtered and concentrated to yield 112.5 mg (90%) of a yellow solid, which required no further purification. This material was dissolved in diethyl ether and treated with 2 N HCl/diethyl ether, stirred at room temperature for 18 hours, filtered, washed with diethyl ether and dried to yield 104 mg (76%) of **134** as a white solid. ^1^H-NMR (CD_3_OD) δ 8.08 (s, 1 H), 7.63 (d, 2 H, J = 8.0 Hz), 7.48 (d, 2 H, J = 8.4 Hz), 7.21 (d, 2 H, J = 8.8 Hz), 6.71 (d, 2 H, J = 8.8 Hz), 4.09-4.01 (m, 1 H), 3.72-3.61 (m, 2 H), 3.49-3.42 (m, 2 H), 2.54-2.44 (m, 2 H). ^13^C-NMR (CD_3_OD, 75 MHz) δ 148.2, 137.1, 132.8, 129.2, 129.1, 128.1, 126.6, 125.5, 125.4, 125.3, 125.2, 124.7, 118.1, 112.2, 50.3, 48.3, 47.0, 29.1. LC-MS, calculated for C_19_H_18_F_3_N_5_ (MH)^+^ 374.4; observed 374.2. Anal. Calculated (with 0.4 mol diethyl ether) for C_19_H_19_ClF_3_N_5_; C, 54.62; H, 5.45; N, 15.46. Found: C, 54.64; H, 5.05; N, 15.05.

**158:** A mixture containing ***int-14*** (50 mg, 0.132 mmol), Boc-glycine (69 mg, 0.394 mmol), diisopropylethylamine (0.15 mL, 0.792 mmol) and Propylphosphonic anhydride solution, 50 wt. % in ethyl acetate (0.25 mL, 0.394 mmol) in anhydrous tetrahydrofuran (20 mL) was sealed tightly and stirred at room temperature for 18 hours. The solvent was diluted with ethyl acetate (20 mL) and saturated aqueous NaHCO_3_ (25 mL). The organic layer was washed with brine, dried (Na_2_SO_4_), filtered and concentrated. The crude material was adsorbed onto silica gel and purified via ISCO using 10–50% ethyl acetate from hexanes to yield 50 mg (0.0933 mmol, 71%) of an off-white solid, which was dissolved in methanol (5 mL), cooled to 0^o^ C and treated with 4.0 N hydrochloric acid in dioxane (1.2 mL, 4.8 mmol). The reaction warmed to room temperature and stirred for 18 hours. Upon completion, the mixture was concentrated and the residue was partitioned between dichloromethane and saturated aqueous NaHCO_3_. The aqueous layer was extracted with dichloromethane and the combined organic layers were washed with brine, dried (Na_2_SO_4_), filtered and concentrated. The crude material was adsorbed onto silica gel and purified via ISCO using 0–10% methanol from dichloromethane to yield 25 mg (63%) of an off-white solid (**158**). ^1^H-NMR (CDCl_3_) δ 9.60 (br s, 1 H), 7.68 (dd, 4 H, J = 8.4 Hz, 9.6 Hz), 7.49 (dd, 4 H, J = 2.0 Hz), 7.29-7.21 (m, 6 H), 3.50 (s, 2 H). LC-MS, calculated for C_24_H_19_F_3_N_4_O(MH)^+^ 436.4; observed 437.2.

**197:** A mixture containing **79** (405 mg, 0.805 mmol), 3-fluorobenzyl bromide (0.11 mL, 0.897 mmol) and triethylamine (0.28 mL, 2.01 mmol) in dimethylformamide (7 mL) was stirred for 25 hours at room temperature. The reaction was poured into a saturated aqueous LiCl solution and extracted with ethyl ether. The organic layer was washed with brine, dried (Na_2_SO_4_), filtered and concentrated to obtain 523 mg of a yellow gel. The crude material was purified over silica gel using 0–5% methanol from dichloromethane to yield 212 mg (43%) of an off-white solid (**197**). ^1^H-NMR (CDCl_3_) δ 9.39 (s, 1 H), 7.71-7.64 (m, 6 H), 7.54-7.47 (m, 6 H), 7.37-7.23 (m, 3 H), 7.11-6.96 (m, 3 H), 3.88 (s, 2 H), 3.47 (s, 2 H). ^13^C-NMR (CDCl_3_, 75 MHz) δ 169.7, 138.3, 133.1, 130.5, 129.4, 128.8, 127.4, 126.7, 125.8, 123.7, 120.3, 115.0, 114.8, 53.6, 52.4. LC-MS, calculated for C_32_H_23_F_7_N_4_O (MH)^+^ 613.5; observed 613.2. Anal. Calculated for C_32_H_23_F_7_N_4_O; C, 62.74; H, 3.78; N, 9.14. Found: C, 62.46; H, 3.86; N, 9.09.

**319:** A solution of ***int-23*** (262 mg, 0.667 mmol), 4-hydroxybenzaldehyde (83 mg, 0.68 mmol) and 4A molecular sieves (300 mg) in anhydrous methanol (7.5 mL) and anhydrous tetrahydrofuran (3.5 mL), was stirred at room temperature for 18 hours. The reaction was cooled to 0^o^ C and treated with sodium borohydride (51 mg, 1.33 mmol); the reaction stirred for 4 hours at room temperature. The reaction was concentrated and the residue partitioned between saturated aqueous sodium bicarbonate solution and ethyl acetate. The combined organic layers were washed with brine, dried (Na_2_SO_4_), filtered and concentrated to yield 390 mg of a yellow gel. The crude material was purified over silica gel using 0.5-5% methanol from dichloromethane to yield 146 mg (44%) of **319** as a white solid. ^1^H-NMR (CDCl_3_) δ 7.30 (d, 2 H, J = 9 Hz), 7.17 (d, 2 H, J = 9 Hz), 6.93 (d, 2 H, J = 9 Hz), 6.72 (d, 2 H, J = 9 Hz), 6.55 (s, 1 H), 3.77 (s, 2 H), 3.72 (dd, 2 H, J = 12 Hz), 2.80 (dd, 2 H, J = 12 Hz), 2.74-2.65 (m, 2 H), 2.36 (br s, 1 H), 2.02 (d, 4 H, J = 12 Hz), 1.89-1.71 (m, 3 H), 1.62-1.22 (m, 7 H). ^13^C NMR (CDCl_3_, 75 MHz) δ 151.5, 129.4, 126.5, 115.6, 115.4, 54.0, 50.2, 47.9, 37.3, 33.0, 32.1, 26.2, 26.0; ESI-MS, calculated for C_28_H_33_F_3_N_4_O (M)^-^ 497.6; observed 497.2. Anal. Calculated for C_28_H_33_F_3_N_4_O; C, 67.45; H, 6.67; N, 11.23. Found: C, 67.19; H, 6.63; N, 11.11.

**354:** A mixture containing **341** (300 mg, 0.82 mmol), 4-cyanobenzyl bromide (164 mg, 0.835 mmol) and triethylamine (0.29 mL, 2.08 mmol) in dimethylformamide (7 mL) was stirred for 18 hours at room temperature. The reaction was poured into a saturated aqueous LiCl solution and extracted with ethyl ether. The organic layer was washed with brine, dried (Na_2_SO_4_), filtered and concentrated. The crude material was purified over silica gel using 1–5% methanol from dichloromethane to yield 212 mg (54%) of a white solid (**354**). ^1^H-NMR (CDCl_3_) δ 9.19 (s, 1 H), 7.68 (d, 2 H, J = 9 Hz), 7.45 (dd, 2 H, J = 6 Hz, 9 Hz), 6.60 (s, 1 H), 3.95 (s, 2 H), 3.46 (s, 2 H), 2.72 (dd, 1 H, J 3 Hz, 6 Hz), 2.07-1.99 (m, 2 H), 1.85-1.72 (m, 5 H), 1.52-1.25 (m, 6 H). ^13^C NMR (CDCl_3_, 75 MHz) δ 169.0, 158.4, 144.2, 137.9, 135.2, 132.6, 128.6, 126.4, 119.4, 111.6, 106.0, 53.5, 52.4, 37.3, 33.0, 26.2, 25.9; ESI-MS, calculated for C_26_H_26_F_3_N_5_O (MH)^+^ 482.5; observed 482.0. Anal. Calculated for C_26_H_26_F_3_N_5_O; C, 64.85; H, 5.44; N, 14.54. Found: C, 64.56; H, 5.51; N, 14.48.

**408:** A mixture containing **17** (100 mg, 0.269 mmol), tert-Butyl 4-oxopiperidine-1-carboxylate (160 mg, 0.809 mmol) and anhydrous sodium sulfate (catalytic amount) in acetic acid (5 mL) was stirred at room temperature for 2 hours. Sodium triacetoxyborohydride (360 mg, 1.61 mmol) was added and the reaction stirred at room temperature for 18 hours. The reaction was concentrated and the residue was partitioned between ethyl acetate and saturated aqueous NaHCO_3_. The crude material was adsorbed onto silica gel and purified via ISCO using 10–75% ethyl acetate from hexanes to yield 100 mg (0.181 mmol, 67%) of an off-white solid, which was dissolved in anhydrous dioxane (5 mL), cooled to 0^o^ C and treated with 4.0 N hydrochloric acid in dioxane (2.25 mL, 9.03 mmol). The reaction warmed to room temperature and stirred for 18 hours. Upon completion, the mixture was concentrated and the residue was partitioned between dichloromethane and saturated aqueous NaHCO_3_. The aqueous layer was extracted with dichloromethane and the combined organic layers were washed with brine, dried (Na_2_SO_4_), filtered and concentrated to obtain 25 mg (30%) of an off-white solid (**408**). ^1^H-NMR (CDCl_3_) δ 7.56 (d, 2 H, J = 8.0 Hz), 7.51 (d, 2 H, J = 8.4 Hz), 7.41 (s, 1 H), 7.00 (dd, 2 H, J = 6.8 Hz, 8.8 Hz), 6.62 (dd, 2 H, J = 8.8 Hz, 9.6 Hz), 3.78-3.61 (m, 4 H), 3.27-3.17 (m, 2 H), 3.12-3.01 (m, 1 H), 2.36-2.23 (m, 2 H). LC-MS, calculated for C_22_H_20_F_6_N_4_(MH)^+^ 455.4; observed 455.2.

### Mammalian and parasitic cell lines

Human monocytes, THP-1 (ATCC, TIB002) were used as host cells for *Leishmania* infection and to assess drug cytotoxicity. The cells were cultured at 37C, 5% CO_2_ in RPMI medium (ATCC) supplemented with 10% FBS, 1% Penicillin/Streptomycin and 0.05 mM of β-mercaptoethanol. Cells were used in experiments up to passage 10.

Luminescent strains, *L. donovani* LV82 expressing firefly luciferase (a kind gift from Dr. Abhay Satoskar, Ohio State University) and *L. mexicana* (NR-51210, ATCC) expressing renilla luciferase were used to evaluate the effect of the compounds in intracellular and extracellular conditions [17]. Wild-type *L. donovani* LV82 (ATCC) were used for Giemsa staining based experiments. All parasites were cultured at 25°C and 5% CO_2_ in M199 media (Corning) supplemented with 10% FBS, 1% Penicillin/Streptomycin, and Hemin (0.01 mg/mL), and used in experiments up to passage 15.

### Luminescent-based evaluation of intracellular anti-leishmanial activity

THP-1 cells were seeded in a 96 well plate (25,000 cells/well) overnight then differentiated with 150 nM phorbol 12-myristate 13-acetate (PMA) over 72 hours (S1 Fig). The resulting macrophages were infected with *Leishmania* promastigotes at a multiplicity of infection of 1:10. Briefly, promastigotes of a known cell density were resuspended in RPMI media (with 10% FBS, 1% P/S and 0.05 mM β-mercaptoethanol) and incubated with adhered THP-1 cells over 18 hours. After infection, cells were washed three times with fresh media to remove extracellular promastigotes.

Infected macrophages were then treated with compounds solubilized in DMSO ranging from 0.1-10 µM in concentrations. After 72 hours of treatment, the viability of the amastigotes within macrophages was determined using Promega firefly luminescence assay (Cat E1500) or Pierce Renilla luciferase assay (Cat 16166) for *L. donovani* and *L. mexicana* strains, respectively. Briefly, media was removed, and cells were lysed with the assay lysis buffer. The luciferase substrate was then incubated with the lysed cells for 10 min at room temperature and luminescence of live *Leishmania* was measured in a white opaque 96 well plate using a Biotek plate reader. The IC_50_ value for each compound was obtained from the best fit curves obtained by plotting the relative luminescence units against the drug concentration.

### Effect of compounds on host cell viability

Effect of the compounds on THP-1 cell viability measured using thiazolyl blue tetrazolium bromide which measures cell metabolic activity (MTT). THP-1 cells were seeded in a 96 well plate (25,000 cells/well) overnight then differentiated with 150 nM phorbol 12-myristate 13-acetate (PMA) over 72 hours. Macrophages were then treated with compounds resuspended in RPMI media (1–50 µM) for 24 hours. Media was replaced with thiazolyl blue tetrazolium bromide solubilized in RPMI media (0.5 mg/mL) and incubated at 37°C for 2 hours. The reduced formazan crystals were solubilized with isopropyl alcohol and the absorbance of the resulting solution was measured at 560 nm with a background subtraction at 670 nm. The concentration required to reduce host cell viability by 50% (24hr LC_50_) value was measured from best fit curves plotting the relative absorbance values against drug concentration. This process was repeated with a 72-hr incubation and extended concentration range (1–300 µM) for select compounds.

### Image-based evaluation of intracellular anti-leishmanial activity

Bone marrow derived macrophages (BMDMs) were isolated from BALB/c mice and cultured as previously described [18,19]. Briefly, bone marrow was harvested from long bones of mice and the isolated cells were seeded at a concentration of 2 x 10^6^ cells/mL in petri dishes with RPMI media supplemented with 10% FBS, 1% Penn-Strep and 10% L929 conditioned media (LCM). Completely differentiated BMDMs are obtained after 7 days of culture with media change every 2 days. Harvested cells are seeded onto 10 mm glass cover slips at 5 x 10^5^ cells/ well of a 24 well plate in DMEM media (without LCM) and allowed to adhere overnight. BMDM cells are then infected overnight with LV82 *L. donovani* or *L. mexicana* (NR-51210) at a multiplicity of infection of 10. After infection, cells were washed three times with fresh media to remove extracellular promastigotes and treated with compounds for a 72-hour incubation. BMDM cells were then washed with phosphate buffered saline, fixed with ice-cold methanol, and stained with Giemsa (5% v/v in water). The cover slips with stained cells are mounted onto glass slides and imaged on EVOS XL (100X, Thermo Fisher Scientific). *Leishmania* amastigotes per 100 macrophages were determined in a blinded manner. The concentration required to reduce intracellular amastigote viability by 50% (IC_50_) value was measured from best fit curves plotting the normalized values against drug concentration.

### Effect of compounds on promastigote viability

The effect of compounds on extracellular promastigotes was evaluated using a resazurin-based assay as described previously [10]. In short, late log phase *Leishmania* promastigotes were seeded in a 96 well plate at 1 x 10^5^ parasites per well and treated with drugs (0.5 – 200 µM) for 72 hours at 25°C. Ten microliters of Resazurin (0.02% w/v) were added to the treated parasites to achieve a final concentration of 0.002% w/v and incubated for 24 hours. Viability of the promastigotes was assessed by fluorescence (excitation 544 nm, emission 590 nm, SpectraMax M2, Molecular Devices). The minimum inhibitory concentration required to reduce promastigote viability by 50% (MIC_50_) value was determined from best fit curves plotting the relative fluorescence values against drug concentration.

### Proteomic analysis via affinity capture

To generate cell lysate, ten million THP-1 cells were plated overnight in a 10 cm dish, then differentiated with 150 nM phorbol 12-myristate 13-acetate (PMA) for 72 hours. The resulting macrophages were infected with *L. mexicana* promastigotes at a multiplicity of infection of 1:10 and incubated for 18 hours. After infection, cells were washed three times with PBS to remove extracellular promastigotes. Cells were then collected by scraping and pelleted with centrifugation. The cell pellet was resuspended in cell buffer (50mM Tris-HCl, 150mM NaCl, 2mM EDTA, 0.5% triton-x-100) and subjected to five freeze-thaw cycles. Samples were centrifuged 14,000g x 10 minutes to remove cellular debris. Protein concentration of supernatant was determined by BCA assay and cell lysate was stored at -80 ° C until further use.

Functionalized agarose beads were made as follows. NHS activated agarose beads were washed three times to remove acetone and resuspended in PBS to create a slurry. 494 (197 with ligation chemical handle) was prepared in 0.3 mL at 25mM in DMSO and 1.7 mL PBS was added to 1 mL of agarose slurry for a final volume of 3 mL at a concentration of 2.5mM (10% v/v DMSO). Beads were incubated with 494 for 2 hrs at room temperature, then washed three times with PBS to remove unbound drug. Beads were then blocked with 1M Tris-HCl (pH 7.5) for 1 hr at room temperature. Beads were washed twice with buffer (100mM Tris-HCl, 300mM NaCl, 2mM EDTA, 0.5% NP-40) and resuspended in 1 mL of buffer. Control beads were generated by coupling 2.5mM butylamine to NHS-agarose beads in the same manner as above.

500 µg cell lysate was incubated with 200 µL slurry at a final volume of 1 mL for 1 hr. Unbound protein was removed through four 15-minute washes. Beads were then boiled in Laemli sample buffer and resolved in a polyacrylamide gel and stained with Coomassie. Lanes (1 cm) for each sample were excised and cut into 1mm cubes and placed in 1ml of destain solution for 2 hours at room temperature (RT). After removal of destain solution, each sample was washed 2x with 100% acetonitrile (ACN) and incubated for 10 min at RT. Proteins were reduced with 10 mM dithiothreitol (DTT) for 10 min at 55 °C, then for 20 min at RT. After DTT was removed, samples were alkylated with 100 mM IAA for 45 min in the dark at RT, and in-gel digested with 20 ng/µl trypsin (Promega) overnight at 37˚C. Peptides were extracted with 200 ul of ACN, gently vortex and incubated at RT for 10 minutes, then transferred to a new tube. This was performed twice. Peptides were desalted with C18 spin columns (Pierce) and then dried via vacuum centrifugation. Peptide samples were stored at -80˚C until further analysis.

The peptide samples were resuspended in 15 μL of 5% ACN/0.1% formic acid and analyzed by LC-MS/MS using an Easy nLC 1200 coupled to a QExactive HF mass spectrometer (Thermo Scientific). Samples were injected onto an Easy Spray PepMap C18 column (75 μm id × 25 cm, 2 μm particle size) (Thermo Scientific) and separated over a 90 minute method. The gradient for separation consisted of 5–45% mobile phase B at a 250 nL/min flow rate, where mobile phase A was 0.1% formic acid in water and mobile phase B consisted of 0.1% formic acid in ACN. The QExactive HF was operated in data-dependent mode where the 15 most intense precursors were selected for subsequent fragmentation. Resolution for the precursor scan (m/z 375–1700) was set to 60,000, while MS/MS scan resolution was set to 15,000. The normalized collision energy was set to 27% for higher-energy collisional dissociation (HCD_. Peptide match was set to preferred, and precursors with unknown charge or a charge state of 1 and ≥ 7 were excluded.

Raw data files were searched against the Uniprot reviewed human database (containing 20,360 entries, downloaded January 2022), a Uniprot *L. mexicana* database (containing 8,269 entries, downloaded April 2022), and the MaxQuant common contaminants database (246 entries), using the Sequest HT search engine node within Proteome Discoverer (v2.5, Thermo Fisher). Enzyme specificity was set to trypsin/P, up to two missed cleavage sites were allowed, methionine oxidation and N-terminus acetylation were set as variable modifications, and cysteine carbamidomethylation was set as a static modification. The Minora node was used to extract label-free quantification (LFQ) intensities. A 1% peptide-level false discovery rate (FDR) and a 5% protein-level FDR were used to filter all data. Match between runs was enabled. Data filtering, imputation, and statistical analysis was performed in Perseus software (version 1.6.14.0). Corrected p-values (q-values) were calculated by permutation FDR method and are reported with the raw data file. Proteins hits were defined as those with Log2(fold-change) > 1 and p-value 0.05 compared to butylamine bead.

### Proteomic analysis via thermal proteome profiling

To generate cell lysate, ten million THP-1 cells were plated overnight in a 10 cm dish, then differentiated with 150 nM phorbol 12-myristate 13-acetate (PMA) for 72 hours. The resulting macrophages were infected with *L. mexicana* promastigotes at a multiplicity of infection of 10 and incubated for 18 hours. After infection, cells were washed three times with PBS to remove extracellular promastigotes. Cells were then collected by scraping and pelleted with centrifugation. The cell pellet was resuspended in phosphate buffered saline and subjected to five freeze-thaw cycles. Samples were centrifuged 14,000g x 10 minutes to remove cellular debris. Protein concentration of supernatant was determined by BCA assay and cell lysate was stored at -80 ° C until further use.

The cell lysate was divided into two portions. One portion was spiked with **197** in DMSO to generate the ‘with ligand’ sample; and the other portion was spiked with DMSO to generate the ‘without ligand’ sample. The final concentration of **197** in the ‘with ligand’ sample was 100 µM, and both the without and with ligand samples contained 1% DMSO. The with and without samples were each subjected five replicate one-pot TPP analyses like that previously described [20–22]. In each replicate aliquots of the with and without samples were distributed into a series of 12 different samples before heating for 3 min at a temperature gradient ranging from 43– 65 °C with 2 °C intervals. After heat treatment, the samples are equilibrated at room temperature for 3 min before placing on ice. The with samples and the without samples in each biological replicate were combined to generate a single with and without ligand sample, respectively. The combined samples were centrifuged at 48000 rpm for 20 min using a TPA100.1 rotor and a Beckman Optima TL ultracentrifuge. The supernatants were transferred into 10 kDa MWCO centrifugal filter units and buffer exchanged to 8 M urea in 0.1 M Tris-HCl pH 8.5 before TCEP reduction and MMTS alkylation. The alkylated proteins were then digested with trypsin, and the peptides generated form the without and with samples from each of the five replicates were labeled with a TMT 10-Plex according to the manufacturer’s protocol. Ultimately, a C18 Macrospin column cleanup was performed on the combined TMT 10-plex sample, sample prior to LC-MS/MS analysis.

The LC-MS/MS analyses were performed using a nanoAcquity UPLC system (Waters) coupled to a Thermo Orbitrap Fusion Lumos mass spectrometer system. The dried peptide material generated from TPP analysis was reconstituted in 15 µL of 1% TFA, 2% acetonitrile in H2O, and a 1 µl aliquot was injected into the system. The peptides were first trapped on a Symmetry C18 20 mm × 180 µm trapping column (5 µL/min at 99.9/0.1 water/acetonitrile, v/v). The analytical separation was performed using an Acquity 75 µm × 250 mm high strength silica (HSS) T3 C18 column with a 1.8 µm particle size (Waters); the column temperature was set to 55 °C. Peptide elution was performed using a 90 min linear gradient of 3–30% ACN with 0.1% formic acid at a flow rate of 400 nL/min. The MS data were collected using a top 20 data-dependent acquisition (DDA) method which included MS1 at 120k and MS2 at 50k resolution. The MS1 AGC target was 4.0 × 10^5^ ions with a max injection time of 50 ms. For MS2, the AGC target was 1.0 × 10^5^ ions with a max injection time of 105 ms. The collision energy was set to 38%, and the scan range was 375– 1500 m/z. The isolation window was 0.7 and the dynamic exclusion duration was 60 s. The peptide sample was subjected to three LC-MS/MS analyses.

Proteome Discoverer 2.2 (Thermo) was used to search the raw LC-MS/MS data against the mouse and *Leishmania* proteins in the 2017-06-07 release of the UniProt Knowledgebase. The raw LC MS/MS data were searched using fixed MMTS modification on cysteine; TMT 10-Plex labeling of lysine side chains and peptide N-termini; variable oxidation of methionine; variable deamidation of asparagine and glutamine; and variable acetylation of the protein N-terminus. Trypsin was set as the enzyme, and up to two missed cleavages were allowed. For peptide and protein quantification, reporter ion abundance was set as intensity, and the normalization mode and scaling mode were each set as none. All other settings were left as the default values. Only proteins/peptides with protein/peptide FDR confidence labeled as “high” (i.e., FDR < 0.01) and with no quantification channels being 0 were used for subsequent analyses. For each biological replicate, a normalization factor was calculated by dividing the ratio of the summed signal intensities recorded in the samples from each biological replicate by the summed signal intensities in the 126 TMT channel. For each identified protein, a ratio of the observed reporter ion intensities in the with sample to the without sample was generated for each biological replicate. The resulting ratio was divided by the normalization factor for each of the replicates. These normalized ratios (fold-change) were then 𝑙𝑜𝑔2-base transformed, averaged, and tested by a two-tailed student’s t-test comparing with a mean of 0. Proteins hits were defined as those with a |z-score| > 1 and p-value < 0.05.

### 197 target validation

B6.129P2-*Lyz2*^*tm1(cre)Ifo*^/J mice [23] (stock #004781, herein termed ‘Lys K/O’) were obtained from Jackson Laboratory. This strain has a nuclear-localized Cre recombinase inserted into the first coding ATG of the lysozyme 2 gene (Lyz2) which eliminates endogenous Lyz2 gene function. Wildtype C57BL6/J mice (stock #000664) were obtained as a control.

Bone marrow derived macrophages (BMDMs) were isolated from both mice and cultured as described above in “Image-based Evaluation of Intracellular Anti-Leishmanial Activity”. BMDMs were seeded onto glass cover slips, infected with LV82 *L. donovani* at a multiplicity of infection of 1:10. Cells were washed to remove extracellular promastigotes and treated with compounds (197, amphotericin B) for a 72-hour incubation. BMDM cells were then washed with phosphate buffered saline, fixed with ice-cold methanol, and stained with Giemsa (5% v/v in water). The cover slips with stained cells are mounted onto glass slides and imaged on EVOS XL (100X, Thermo Fisher Scientific). Leishmania amastigotes per 100 macrophages were determined in a blinded manner. The concentration required to reduce intracellular amastigote viability by 50% (IC_50_) value was measured from best fit curves plotting the normalized values against drug concentration.

### Lysozyme activity

Recombinant human lysozyme protein (1mg/mL, Sigma, Cat # L1667) was incubated with 0 µM, 1 µM or 50 µM **197**. Lysozyme activity was measured via a fluorometric assay (Abcam, Cat # ab211113) according to manufacturer’s protocol.

## Supporting information

S1 Table Chemical Structures of all compounds screened. (DOCX)

S2 Table Results of primary screen in all compounds. Concentration at which intracellular *Leishmania donovani* burden is reduced by 50% in THP1 macrophages (Lum IC50) as identified by luminescence assay. Concentration where THP1 macrophage cell viability is 50% (LC50) after 24-hour incubation with compound as determined by MTT assay. Selectivity between host-directed effect and cytotoxicity, defined as 24h LC50/ Lum IC50. Parental compound AR-12 provided for reference. Compounds highlighted in grey have higher selectivity than parental compound AR-12. Compounds highlighted in yellow were selected for secondary screening. ND = not determined.
(DOCX)

S1 Fig Initial Screening Approach. Initial Screening Approach. Schematic illustrating the medium-throughput luminescence-based assay to determine effect of compounds on intracellular Leishmania infection and parallel screening to determine effect of compounds on host cell viability. Created in BioRender. Gurysh, E. (2026) https://BioRender.com/p5az9fi.
(DOCX)

S2 Fig Venn diagram demonstrating compound potency against intracellular *L. donovani* (Lum IC_50_) and cytotoxicity against THP-1 host cell (24 hr LC_50_) relative to parental compound AR-12. (DOCX)

S3 Fig Chemical structures of 16 hit compounds derived from AR-12 with associated core, R1, and R2 modifications highlighted. (DOCX)

S4 Fig Luminescent activity of intracellular *L. donovani* infected THP1 macrophage cell after 72-hour incubation with compounds. (DOCX)

S5 Fig Graphs of THP1 macrophage cell viability after 24-hour incubation with compounds as determined by MTT assay. (DOCX)

S6 Fig Graphs of THP1 macrophage cell viability after 72-hour incubation with compounds as determined by MTT assay. (DOCX)

S7 Fig Dose response of extracellular *Leishmania* promastigote viability after 72-hour incubation with compound as measured by resazurin assay. (DOCX)

S8 Fig Dose response of intracellular *Leishmania donovani* burden in bone marrow derived macrophages after 72-hour incubation with compound as identified image-based Giemsa staining. (DOCX)

S9 Fig A) Chemical structure of 197 chemically modified for conjugation to agarose bead for affinity capture proteomic analysis. **B)** Luminescent activity of intracellular *L. donovani* infected THP1 macrophage cell after 72-hour incubation with 197 compared to chemically modified 197.
(DOCX)

S10 Fig A) All human proteins (gray circle) identified by affinity capture using 197 functionalized bead plotted Log2(fold-change) and -Log10(p-value) over control bead. **Significant proteins Log2FC > 1 and p < 0.05 are shown with black circles. B)** All human proteins (gray circle) identified by thermal profile analysis with 197 plotted z-score and -Log10(p-value). Significant proteins with a |z-score| > 1 and p < 0.05 are shown with black circles. **C)** All *Leishmania* proteins (gray circle) identified by affinity capture using 197 functionalized bead plotted Log2(fold-change) and -Log10(p-value) over control bead. Significant proteins Log2FC > 1 and p < 0.05 are shown with black circles. Proteins with a (|z-score| > 1 and p < 0.05 overlapping with significant proteins identified by affinity capture (Log2FC > 2 and p < 0.05) are shown with open circles. **D)** All *Leishmania* proteins (gray circle) identified by thermal profile analysis with 197 plotted z-score and -Log10(p-value). Significant proteins with a |z-score| > 1 and p < 0.05 are shown with black circles. Significant proteins Log2FC > 1 and p < 0.05 are shown with black circles. Proteins with a (|z-score| > 1 and p < 0.05 overlapping with significant proteins identified by affinity capture (Log2FC > 2 and p < 0.05) are shown with open circles. **E)** Values for three *Leishmania* proteins overlapping between two proteomic approaches.
(DOCX)

S11 Fig Dose response of amphotericin B on intracellular *Leishmania* burden in bone marrow derived macrophages derived from wildtype C57BL/6 (WT, black circle) or lysozyme knockout mice (Lys K/O, gray triangle) as identified image-based Giemsa staining. Data is presented as mean ± standard deviation of biological triplicates.
(DOCX)

S1 File Synthesis and Characterization of Selected Analogs. (DOCX)
