# Supplementary figures and images for: Synthesis and preliminary evaluation of novel compounds that demonstrate broad host-directed anti-leishmanial activity
Source: PLoS Negl Trop Dis. 2026 Jul 13;20(7):e0014520. doi: 10.1371/journal.pntd.0014520 (PMC13379085; doi:10.1371/journal.pntd.0014520)

**S1 Table.** Chemical Structures of all compounds screened.

| **AR-12** | **1** | **2** | **3** | **4** | **5** | **6** |
| --- | --- | --- | --- | --- | --- | --- |
|  |  |  |  |  |  |  |
| **7** | **8** | **9** | **10** | **11** | **12** | **13** |
|  |  |  |  |  |  |  |
| **14** | **15** | **16** | **17** | **18** | **19** | **20** |
|  |  |  |  |  |  |  |
| **21** | **22** | **23** | **24** | **25** | **26** | **27** |
|  |  |  |  |  |  |  |
| **28** | **29** | **30** | **31** | **32** | **33** | **34** |
|  |  |  |  |  |  |  |
| **35** | **36** | **37** | **38** | **39** | **40** | **41** |
|  |  |  |  |  |  |  |
| **42** | **43** | **44** | **45** | **46** | **47** | **48** |
|  |  |  |  |  |  |  |
| **49** | **50** | **51** | **52** | **53** | **54** | **55** |
|  |  |  |  |  |  |  |
| **56** | **57** | **58** | **59** | **60** | **61** | **62** |
|  | 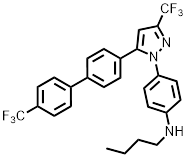 |  |  |  |  |  |
| **63** | **64** | **65** | **66** | **67** | **68** | **69** |
|  |  |  |  |  |  |  |

| **70** | **71** | **72** | **73** | **74** | **75** | **76** |
| --- | --- | --- | --- | --- | --- | --- |
|  |  |  |  |  |  |  |
| **77** | **78** | **79** | **80** | **81** | **82** | **83** |
|  |  |  |  |  |  |  |
| **84** | **85** | **86** | **87** | **88** | **89** | **90** |
|  |  |  |  |  |  |  |
| **91** | **92** | **93** | **94** | **95** | **96** | **97** |
|  |  | 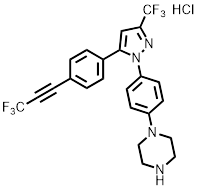 |  |  |  |  |
| **98** | **99** | **100** | **101** | **103** | **104** | **105** |
|  |  |  | 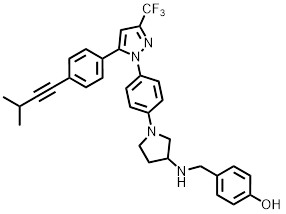 |  |  |  |

| **106** | **107** | **108** | **109** | **110** | **111** | **112** |
| --- | --- | --- | --- | --- | --- | --- |
|  |  |  | 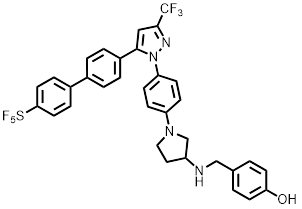 |  |  |  |
| **113** | **114** | **115** | **116** | **117** | **118** | **119** |
|  |  |  |  |  |  |  |
| **120** | **121** | **122** | **123** | **124** | **125** | **126** |
|  |  |  |  |  |  | 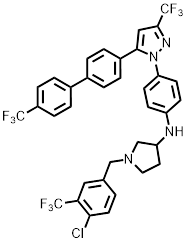 |
| **127** | **128** | **129** | **130** | **131** | **132** | **133** |
|  |  |  |  |  |  |  |
| **134** | **135** | **136** | **137** | **138** | **139** | **140** |
|  |  |  |  |  |  |  |

| **141** | **142** | **143** | **144** | **145** | **146** | **147** |
| --- | --- | --- | --- | --- | --- | --- |
|  |  |  |  |  |  |  |
| **148** | **149** | **150** | **151** | **152** | **153** | **154** |
|  |  |  |  |  |  |  |
| **155** | **156** | **157** | **158** | **168** | **172** | **174** |
|  |  |  |  |  |  |  |
| **175** | **176** | **177** | **178** | **179** | **180** | **181** |
|  |  |  |  |  |  |  |
| **182** | **183** | **184** | **185** | **186** | **187** | **188** |
|  |  | 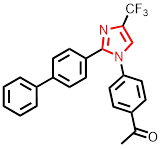 |  |  |  |  |

| **189** | **190** | **191** | **192** | **193** | **194** | **195** |
| --- | --- | --- | --- | --- | --- | --- |
|  |  |  |  |  |  |  |
| **196** | **197** | **198** | **199** | **201** | **202** | **203** |
|  |  |  |  |  |  |  |
| **204** | **205** | **206** | **207** | **208** | **209** | **210** |
|  |  |  |  |  |  |  |
| **211** | **212** | **213** | **214** | **215** | **216** | **217** |
|  |  |  |  |  |  |  |
| **218** | **219** | **220** | **221** | **222** | **229** | **230** |
|  |  |  |  |  |  |  |

| **231** | **232** | **243** | **244** | **245** | **246** | **247** |
| --- | --- | --- | --- | --- | --- | --- |
|  | 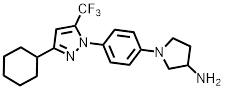 |  |  |  |  |  |
| **248** | **249** | **250** | **251** | **252** | **253** | **254** |
|  |  |  |  |  | 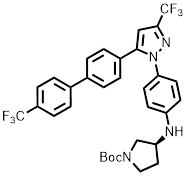 |  |
| **255** | **256** | **257** | **259** | **260** | **261** | **266** |
|  |  |  |  |  |  |  |
| **267** | **268** | **269** | **272** | **273** | **274** | **275** |
|  |  |  |  |  |  |  |
| **276** | **277** | **278** | **279** | **280** | **281** | **282** |
|  |  |  |  |  |  |  |

| **283** | **284** | **285** | **286** | **291** | **292** | **293** |
| --- | --- | --- | --- | --- | --- | --- |
|  |  |  |  |  |  |  |
| **294** | **295** | **296** | **297** | **298** | **299** | **312** |
|  |  |  |  |  |  |  |
| **313** | **314** | **315** | **316** | **317** | **318** | **319** |
|  |  |  |  |  |  |  |
| **320** | **321** | **322** | **323** | **324** | **327** | **328** |
|  |  |  |  |  |  |  |
| **329** | **330** | **331** | **332** | **333** | **334** | **335** |
|  |  |  |  |  |  |  |

| **336** | **337** | **338** | **339** | **340** | **341** | **352** |
| --- | --- | --- | --- | --- | --- | --- |
|  |  |  |  |  |  |  |
| **353** | **354** | **355** | **356** | **357** | **358** | **362** |
|  |  |  |  |  |  |  |
| **363** | **364** | **365** | **366** | **367** | **368** | **370** |
|  |  |  |  |  |  |  |
| **371** | **372** | **373** | **374** | **375** | **376** | **377** |
|  |  |  |  |  |  |  |
| **378** | **381** | **389** | **392** | **394** | **395** | **396** |
|  |  |  |  |  |  |  |

| **397** | **402** | **403** | **404** | **405** | **406** | **408** |
| --- | --- | --- | --- | --- | --- | --- |
|  |  |  |  |  |  |  |
| **409** | **411** | **412** | **413** | **414** | **415** | **416** |
|  |  |  |  |  |  |  |
| **417** | **418** | **419** | **420** | **421** | **422** | **423** |
|  |  |  |  |  |  |  |
| **424** | **425** | **426** | **427** | **428** | **429** | **430** |
|  |  |  |  |  |  |  |
| **431** | **432** | **433** | **434** | **435** | |
|  |  |  |  |  | |

Supplement: S1 Table — (DOCX) [file pntd.0014520.s001.docx]
