# Supplementary material for: Synthesis and preliminary evaluation of novel compounds that demonstrate broad host-directed anti-leishmanial activity
Source: PLoS Negl Trop Dis. 2026 Jul 13;20(7):e0014520. doi: 10.1371/journal.pntd.0014520 (PMC13379085; doi:10.1371/journal.pntd.0014520)
Supplement: S2 Table — Concentration at which intracellular Leishmania donovani burden is reduced by 50% in THP1 macrophages (Lum IC50) as identified by luminescence assay. Concentration where THP1 macrophage cell viability is 50% (LC50) after 24-hour incubation with compound as determined by MTT assay. Selectivity between host-directed effect and cytotoxicity, defined as 24h LC50/ Lum IC50. Parental compound AR-12 provided for reference. Compounds highlighted in grey have higher selectivity than parental compound AR-12. Compounds highlighted in yellow were selected for secondary screening. ND = not determined. (DOCX) [file pntd.0014520.s002.docx]

**S2 Table. Results of primary screen in all compounds.** Concentration at which intracellular *Leishmania donovani* burden is reduced by 50% in THP1 macrophages (Lum IC_50_) as identified by luminescence assay. Concentration where THP1 macrophage cell viability is 50% (LC_50_) after 24-hour incubation with compound as determined by MTT assay. Selectivity between host-directed effect and cytotoxicity, defined as 24h LC_50_ / Lum IC_50_. Parental compound AR-12 provided for reference. Compounds highlighted in grey have higher selectivity than parental compound AR-12. Compounds highlighted in yellow were selected for secondary screening. ND = not determined.

| **CMPD** | **24h LC_50_ (µM)** | **Lum IC_50_ (µM)** | **Selectivity** |
| --- | --- | --- | --- |
| **AR-12** | **13.3** | **3.4** | **3.9** |
| 1 | 19.1 | >10 | <1.9 |
| 2 | >50 | >10 | ND |
| 3 | 11.0 | 1.8 | 6.1 |
| 4 | >50 | >10 | ND |
| 5 | 13.3 | 2.3 | 5.8 |
| 6 | >50 | >10 | ND |
| 7 | 5.2 | 2.4 | 2.1 |
| 8 | >50 | >10 | ND |
| 9 | >50 | >10 | ND |
| 10 | >50 | >10 | ND |
| 11 | 1.4 | >10 | <0.1 |
| 12 | >50 | >10 | ND |
| 13 | >50 | >10 | ND |
| 14 | 50.0 | >10 | <5.0 |
| 15 | >50 | >10 | ND |
| 16 | >50 | >10 | ND |
| 17 | >50 | >10 | ND |
| 18 | 24.5 | 4.0 | 6.1 |
| 19 | >50 | >10 | ND |
| 20 | >50 | >10 | ND |
| 21 | >50 | >10 | ND |
| 22 | >50 | >10 | ND |
| 23 | 5.8 | 0.4 | 14.8 |
| 24 | >50 | >10 | ND |
| 25 | 28.9 | 0.7 | 43.8 |
| 26 | 5.8 | >10 | <0.6 |
| 27 | >50 | >10 | ND |
| 28 | >50 | >10 | ND |
| 29 | >50 | >10 | ND |
| 30 | >50 | >10 | ND |
| 31 | >50 | >10 | ND |
| 32 | 6.3 | >10 | <0.6 |
| 33 | 1.5 | >10 | <0.2 |
| 34 | >50 | 6.0 | >8.3 |
| 35 | >50 | >10 | ND |
| 36 | >50 | >10 | ND |
| 37 | >50 | >10 | ND |
| 38 | >50 | >10 | ND |
| 39 | >50 | >10 | ND |
| 40 | >50 | >10 | ND |
| 41 | >50 | >10 | ND |
| 42 | 0.7 | 4.2 | 0.2 |
| 43 | 0.5 | 4.8 | 0.1 |
| 44 | 7.4 | 0.9 | 8.1 |
| 45 | 3.2 | 3.7 | 0.9 |
| 46 | 10.0 | >10 | <1.0 |
| 47 | >50 | >10 | ND |
| 48 | >50 | >10 | ND |
| 49 | 48.8 | >10 | <4.8 |
| 50 | 23.5 | 1.5 | 16.0 |
| 51 | 25.7 | 8.2 | 3.1 |
| 52 | >50 | >10 | ND |
| 53 | >50 | 0.4 | >112.6 |
| 54 | 9.9 | 2.4 | 4.1 |
| 55 | 29.7 | 8.5 | 3.5 |
| 56 | >50 | >10 | ND |
| 57 | >50 | >10 | ND |
| 58 | 2.6 | 2.2 | 1.2 |
| 59 | >50 | >10 | ND |
| 60 | >50 | >10 | ND |
| 61 | 6.1 | 2.3 | 2.7 |
| 62 | >50 | >10 | ND |
| 63 | >50 | >10 | ND |
| 64 | >50 | >10 | ND |
| 65 | 4.0 | 2.3 | 1.7 |
| 66 | >50 | >10 | ND |
| 67 | >50 | >10 | ND |
| 68 | 34.7 | >10 | <3.5 |
| 69 | 12.4 | 5.5 | 2.3 |
| 70 | >50 | >10 | ND |
| 71 | 8.4 | >10 | <0.8 |
| 72 | >50 | >10 | ND |
| 73 | 4.8 | 2.7 | 1.8 |
| 74 | 6.5 | 2.8 | 2.3 |
| 75 | 48.8 | >10 | <4.9 |
| 76 | 33.2 | >10 | <3.3 |
| 77 | >50 | >10 | ND |
| 78 | >50 | >10 | ND |
| 79 | 6.3 | 7.1 | 0.9 |
| 80 | 8.2 | 3.0 | 2.7 |
| 81 | >50 | >10 | ND |
| 82 | >50 | >10 | ND |
| 83 | 6.2 | 1.8 | 3.5 |
| 84 | >50 | 6.5 | >7.7 |
| 85 | >50 | >10 | ND |
| 86 | 21.9 | 1.4 | 16.0 |
| 87 | >50 | 6.7 | >7.4 |
| 88 | >50 | >10 | ND |
| 89 | 8.0 | 4.0 | 2.0 |
| 90 | >50 | >10 | ND |
| 91 | >50 | 1.4 | >36.1 |
| 92 | 14.7 | >10 | <1.5 |
| 93 | 40.0 | >10 | <4.0 |
| 94 | 29.0 | >10 | <2.9 |
| 95 | >50 | >10 | ND |
| 96 | 37.5 | >10 | <3.8 |
| 97 | >50 | >10 | ND |
| 98 | 19.5 | >10 | <1.9 |
| 99 | 29.2 | >10 | <2.9 |
| 100 | >50 | >10 | ND |
| 101 | 0.5 | 1.1 | 0.4 |
| 103 | >50 | >10 | ND |
| 104 | >50 | >10 | ND |
| 105 | >50 | >10 | ND |
| 106 | 6.0 | 8.1 | 0.7 |
| 107 | >50 | >10 | ND |
| 108 | >50 | >10 | ND |
| 109 | 9.4 | 3.3 | 2.8 |
| 110 | 8.5 | 4.0 | 2.1 |
| 111 | 5.2 | 2.9 | 1.8 |
| 112 | 6.3 | >10 | <0.6 |
| 113 | 19.0 | >10 | <1.9 |
| 114 | 13.6 | >10 | <1.4 |
| 115 | 13.2 | 7.4 | 1.8 |
| 116 | >50 | 9.9 | >5.0 |
| 117 | >50 | 6.1 | >8.2 |
| 118 | 6.2 | 2.6 | 2.4 |
| 119 | 37.2 | >10 | <3.7 |
| 120 | >50 | >10 | ND |
| 126 | >50 | >10 | ND |
| 127 | >50 | >10 | ND |
| 128 | >50 | >10 | ND |
| 129 | 6.3 | 0.1 | 46.7 |
| 130 | 4.2 | 0.2 | 23.9 |
| 131 | 37.2 | >10 | <3.7 |
| 132 | >50 | >10 | ND |
| 133 | 29.0 | 0.9 | 32.5 |
| 134 | 22.6 | 0.5 | 42.1 |
| 135 | >50 | >10 | ND |
| 136 | >50 | >10 | ND |
| 137 | 7.3 | 4.4 | 1.7 |
| 138 | >50 | >10 | ND |
| 139 | >50 | >10 | ND |
| 140 | >50 | >10 | ND |
| 141 | 7.7 | 3.6 | 2.1 |
| 142 | >50 | >10 | ND |
| 143 | >50 | >10 | ND |
| 144 | 5.4 | >10 | <0.5 |
| 145 | >50 | >10 | ND |
| 146 | >50 | 4.4 | >11.4 |
| 147 | >50 | >10 | ND |
| 148 | >50 | >10 | ND |
| 149 | 28.8 | 9.1 | 3.2 |
| 150 | >50 | >10 | ND |
| 151 | 31.8 | >10 | <3.2 |
| 152 | 43.6 | >10 | <4.4 |
| 153 | >50 | >10 | ND |
| 154 | 25.5 | 2.9 | 8.9 |
| 155 | 6.1 | 4.0 | 1.5 |
| 156 | 20.8 | 5.8 | 3.6 |
| 157 | 27.6 | >10 | <2.8 |
| 158 | 16.0 | 0.2 | 81.0 |
| 168 | >50 | 10.0 | >5.0 |
| 172 | >50 | >10 | ND |
| 174 | >50 | >10 | ND |
| 175 | >50 | >10 | ND |
| 176 | >50 | >10 | ND |
| 177 | >50 | >10 | ND |
| 178 | >50 | >10 | ND |
| 179 | 45.0 | >10 | <4.5 |
| 180 | >50 | >10 | ND |
| 181 | 28.7 | >10 | <2.9 |
| 182 | 18.0 | 5.9 | 3.0 |
| 183 | >50 | >10 | ND |
| 184 | >50 | 5.0 | >10 |
| 185 | >50 | >10 | ND |
| 186 | >50 | >10 | ND |
| 187 | >50 | >10 | ND |
| 188 | 9.0 | 3.1 | 2.9 |
| 189 | >50 | >10 | ND |
| 190 | >50 | >10 | ND |
| 191 | 12.0 | >10 | <1.2 |
| 192 | >50 | >10 | ND |
| 193 | >50 | >10 | ND |
| 194 | >50 | >10 | ND |
| 195 | 38.6 | >10 | <3.9 |
| 196 | >50 | >10 | ND |
| 197 | >50 | 2.4 | >20.6 |
| 198 | >50 | >10 | ND |
| 199 | 9.4 | 1.3 | 7.2 |
| 201 | >50 | >10 | ND |
| 202 | >50 | 8.5 | >5.9 |
| 203 | 11.8 | 2.9 | 4.1 |
| 204 | 37.2 | 8.6 | 4.3 |
| 205 | >50 | >10 | ND |
| 206 | >50 | >10 | ND |
| 207 | >50 | >10 | ND |
| 208 | 31.0 | 5.7 | 5.4 |
| 209 | 10.2 | 3.0 | 3.4 |
| 210 | 10.5 | 2.5 | 4.3 |
| 211 | 13.2 | 2.1 | 6.3 |
| 212 | >50 | >10 | ND |
| 213 | >50 | >10 | ND |
| 214 | >50 | >10 | ND |
| 215 | >50 | >10 | ND |
| 216 | >50 | >10 | ND |
| 217 | >50 | >10 | ND |
| 218 | >50 | >10 | ND |
| 219 | >50 | >10 | ND |
| 220 | 15.4 | 1.3 | 11.8 |
| 221 | >50 | >10 | ND |
| 222 | >50 | >10 | ND |
| 229 | 5.0 | 3.4 | 1.5 |
| 230 | 9.5 | >10 | <1.0 |
| 231 | 10.8 | 1.8 | 6.2 |
| 232 | 14.4 | 2.6 | 5.6 |
| 243 | >50 | >10 | ND |
| 244 | >50 | >10 | ND |
| 245 | >50 | >10 | ND |
| 246 | 5.5 | 3.8 | 1.4 |
| 247 | 7.6 | 3.3 | 2.3 |
| 248 | 14.4 | 2.6 | 5.6 |
| 249 | 50.0 | >10 | <5.0 |
| 250 | >50 | >10 | ND |
| 251 | >50 | >10 | ND |
| 252 | 14.4 | 2.3 | 6.2 |
| 253 | >50 | >10 | ND |
| 254 | 50.0 | >10 | <5.0 |
| 255 | >50 | >10 | ND |
| 256 | 6.2 | 2.5 | 2.5 |
| 257 | 5.0 | 2.5 | 2.0 |
| 259 | 23.6 | >10 | <2.4 |
| 260 | 13.9 | 4.3 | 3.2 |
| 261 | 22.4 | 3.1 | 7.3 |
| 266 | >50 | >10 | ND |
| 267 | >50 | >10 | ND |
| 268 | >50 | >10 | ND |
| 269 | >50 | >10 | ND |
| 272 | 17.2 | 5.6 | 3.1 |
| 273 | 15.6 | 4.9 | 3.2 |
| 274 | >50 | >10 | ND |
| 275 | >50 | >10 | ND |
| 276 | 32.2 | >10 | <0.6 |
| 277 | >50 | >10 | ND |
| 278 | >50 | >10 | ND |
| 279 | >50 | >10 | ND |
| 280 | >50 | >10 | ND |
| 281 | 14.3 | 2.0 | 7.2 |
| 282 | >50 | >10 | ND |
| 283 | >50 | >10 | ND |
| 284 | >50 | >10 | ND |
| 285 | >50 | >10 | ND |
| 286 | >50 | >10 | ND |
| 291 | >50 | >10 | ND |
| 292 | >50 | >10 | ND |
| 293 | >50 | >10 | ND |
| 294 | 28.2 | >10 | <2.8 |
| 295 | >50 | >10 | ND |
| 296 | 12.9 | 3.0 | 4.3 |
| 297 | >50 | >10 | ND |
| 298 | >50 | >10 | ND |
| 299 | 31.6 | >10 | <3.2 |
| 312 | 25.5 | 4.8 | 5.3 |
| 313 | >50 | >10 | ND |
| 314 | 21.9 | 2.7 | 8.0 |
| 315 | 25.1 | 3.3 | 7.6 |
| 316 | >50 | >10 | ND |
| 317 | >50 | >10 | ND |
| 318 | 27.0 | 1.8 | 14.8 |
| 319 | 6.4 | 0.8 | 8.1 |
| 320 | >50 | >10 | ND |
| 321 | 23.9 | 5.1 | 4.7 |
| 322 | >50 | >10 | ND |
| 323 | 28.3 | 8.9 | 3.2 |
| 324 | 27.5 | 1.9 | 14.2 |
| 327 | >50 | >10 | ND |
| 328 | 11.1 | 2.6 | 4.3 |
| 329 | >50 | >10 | ND |
| 330 | >50 | 6.2 | >8.1 |
| 331 | 47.0 | 9.9 | 4.7 |
| 332 | 31.2 | 8.0 | 3.9 |
| 333 | >50 | >10 | ND |
| 334 | 24.5 | 2.5 | 9.7 |
| 335 | >50 | >10 | ND |
| 336 | 15.9 | 2.4 | 6.6 |
| 337 | 32.1 | 3.5 | 9.3 |
| 338 | >50 | >10 | ND |
| 339 | 18.2 | 1.7 | 10.4 |
| 340 | >50 | >10 | ND |
| 341 | 26.5 | 4.0 | 6.6 |
| 352 | 29.3 | >10 | <2.9 |
| 353 | 17.5 | 4.2 | 4.2 |
| 354 | 22.0 | 0.5 | 44.9 |
| 355 | 21.2 | 2.0 | 10.5 |
| 356 | 15.5 | 2.0 | 7.6 |
| 357 | 13.1 | 1.2 | 10.7 |
| 358 | 13.2 | >10 | <1.3 |
| 362 | 20.0 | 2.2 | 9.0 |
| 363 | >50 | >10 | ND |
| 364 | 31.1 | 6.8 | 4.5 |
| 365 | 32.9 | >10 | <3.1 |
| 366 | 30.2 | >10 | <3.3 |
| 367 | >50 | >10 | ND |
| 368 | 12.1 | 1.6 | 7.6 |
| 370 | 19.5 | 2.6 | 7.5 |
| 371 | >50 | 8.9 | >5.6 |
| 372 | >50 | >10 | ND |
| 373 | >50 | 7.0 | >7.2 |
| 374 | 8.2 | 3.1 | 2.6 |
| 375 | 12.3 | 6.3 | 1.9 |
| 376 | >50 | >10 | ND |
| 377 | 14.6 | 8.3 | 1.8 |
| 378 | >50 | >10 | ND |
| 381 | 12.4 | 6.2 | 2.0 |
| 389 | >50 | >10 | ND |
| 392 | 25.9 | 4.7 | 5.5 |
| 394 | 30.9 | 5.5 | 5.6 |
| 395 | 37.4 | 7.6 | 4.9 |
| 396 | 23.3 | 6.1 | 3.8 |
| 397 | 27.6 | 8.1 | 3.4 |
| 402 | 26.9 | >10 | <3.0 |
| 403 | >50 | >10 | ND |
| 404 | >50 | >10 | ND |
| 405 | >50 | >10 | ND |
| 406 | >50 | >10 | ND |
| 407 | >50 | >10 | ND |
| 408 | 25.4 | 1.5 | 17.0 |
| 409 | 35.2 | 8.8 | 4.0 |
| 411 | >50 | >10 | ND |
| 412 | >50 | >10 | ND |
| 413 | >50 | >10 | ND |
| 414 | >50 | >10 | ND |
| 415 | >50 | >10 | ND |
| 416 | 18.2 | 1.3 | 14.2 |
| 417 | 24.4 | 3.0 | 8.1 |
| 418 | 12.6 | 3.0 | 4.2 |
| 419 | 21.6 | 2.7 | 7.9 |
| 420 | 7.0 | 1.0 | 6.7 |
| 421 | 19.0 | 1.5 | 12.5 |
| 422 | 6.1 | 2.5 | 2.4 |
| 423 | 3.4 | 6.0 | 0.6 |
| 424 | 6.5 | 2.4 | 2.7 |
| 425 | 5.5 | 2.2 | 2.5 |
| 426 | 31.9 | 4.4 | 7.3 |
| 427 | 29.5 | 6.1 | 4.8 |
| 428 | 11.4 | 8.9 | 1.3 |
| 429 | 12.1 | 2.4 | 5.1 |
| 430 | 16.5 | 8.7 | 1.9 |
| 431 | 22.4 | 4.8 | 4.7 |
| 432 | 5.6 | 2.9 | 2.0 |
| 433 | 27.0 | 3.8 | 7.1 |
| 434 | 24.6 | >10 | <2.5 |
| 435 | 26.9 | >10 | <2.7 |
