# Supplementary material for: Synthesis and preliminary evaluation of novel compounds that demonstrate broad host-directed anti-leishmanial activity
Source: PLoS Negl Trop Dis. 2026 Jul 13;20(7):e0014520. doi: 10.1371/journal.pntd.0014520 (PMC13379085; doi:10.1371/journal.pntd.0014520)
Supplement: S1 Fig — Initial Screening Approach. Schematic illustrating the medium-throughput luminescence-based assay to determine effect of compounds on intracellular Leishmania infection and parallel screening to determine effect of compounds on host cell viability. Created in BioRender. Gurysh, E. (2026) https://BioRender.com/p5az9fi. (DOCX) [file pntd.0014520.s003.docx]

**
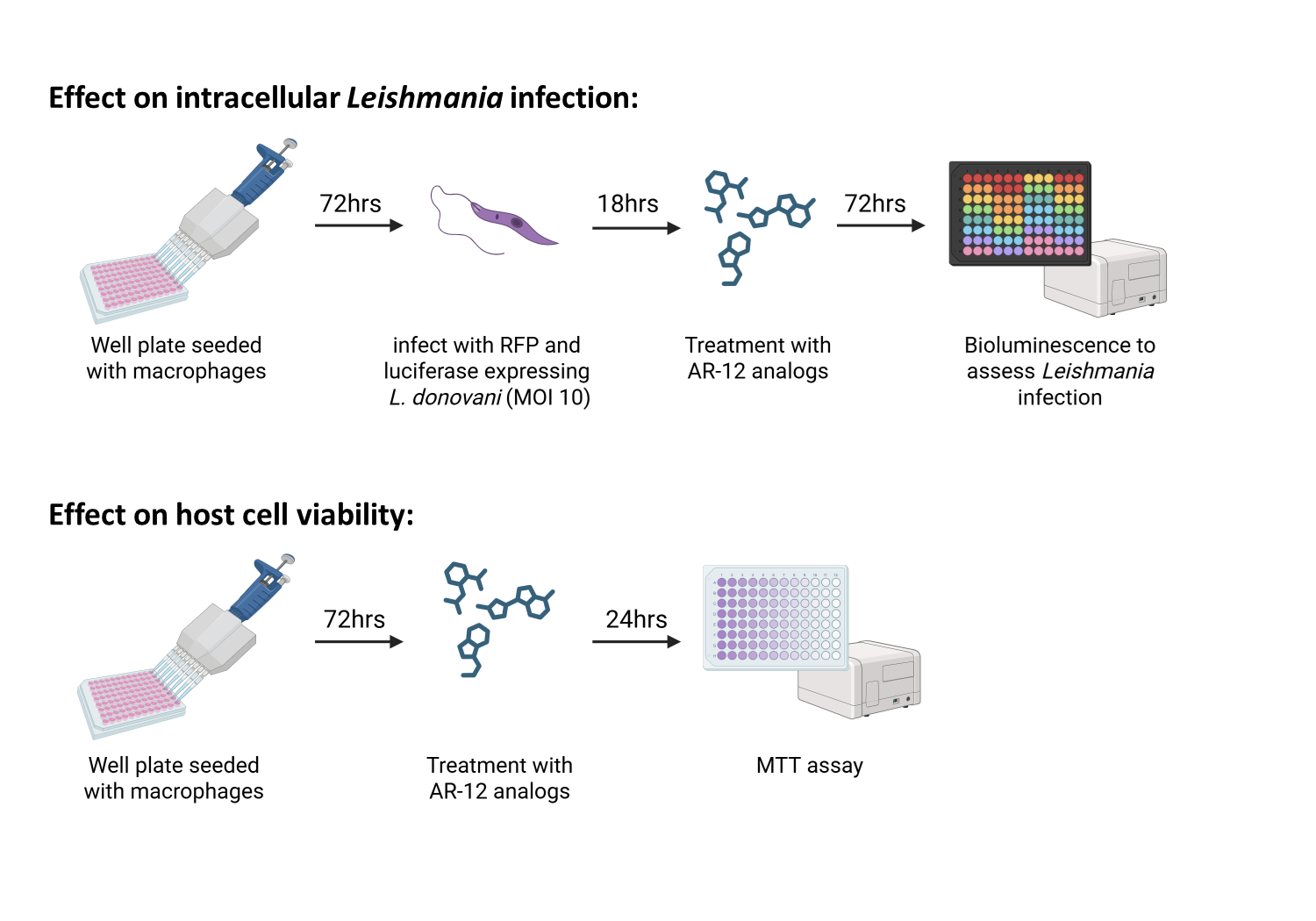
**

**S1 Fig. Initial Screening Approach.** Schematic illustrating the medium-throughput luminescence-based assay to determine effect of compounds on intracellular *Leishmania* infection and parallel screening to determine effect of compounds on host cell viability.
