# Supplementary material for: Synthesis and preliminary evaluation of novel compounds that demonstrate broad host-directed anti-leishmanial activity
Source: PLoS Negl Trop Dis. 2026 Jul 13;20(7):e0014520. doi: 10.1371/journal.pntd.0014520 (PMC13379085; doi:10.1371/journal.pntd.0014520)
Supplement: S2 Fig — (DOCX) [file pntd.0014520.s004.docx]

**
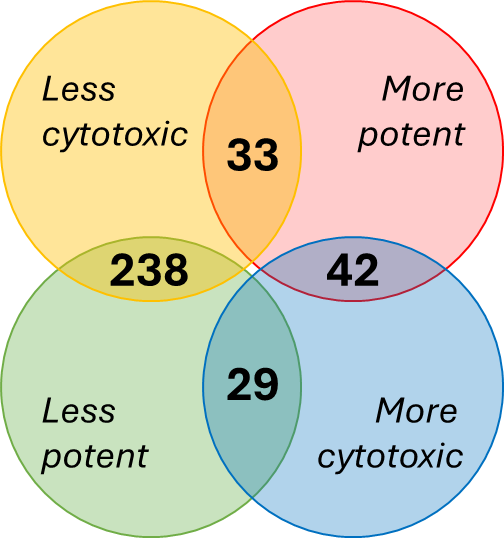
**

**S2 Fig.** Venn diagram demonstrating compound potency against *intracellular L. donovani* (Lum IC_50_) and cytotoxicity against THP-1 host cell (24 hr LC_50_) relative to parental compound AR-12.
