# Supplementary material for: Synthesis and preliminary evaluation of novel compounds that demonstrate broad host-directed anti-leishmanial activity
Source: PLoS Negl Trop Dis. 2026 Jul 13;20(7):e0014520. doi: 10.1371/journal.pntd.0014520 (PMC13379085; doi:10.1371/journal.pntd.0014520)
Supplement: S3 Fig — (DOCX) [file pntd.0014520.s005.docx]

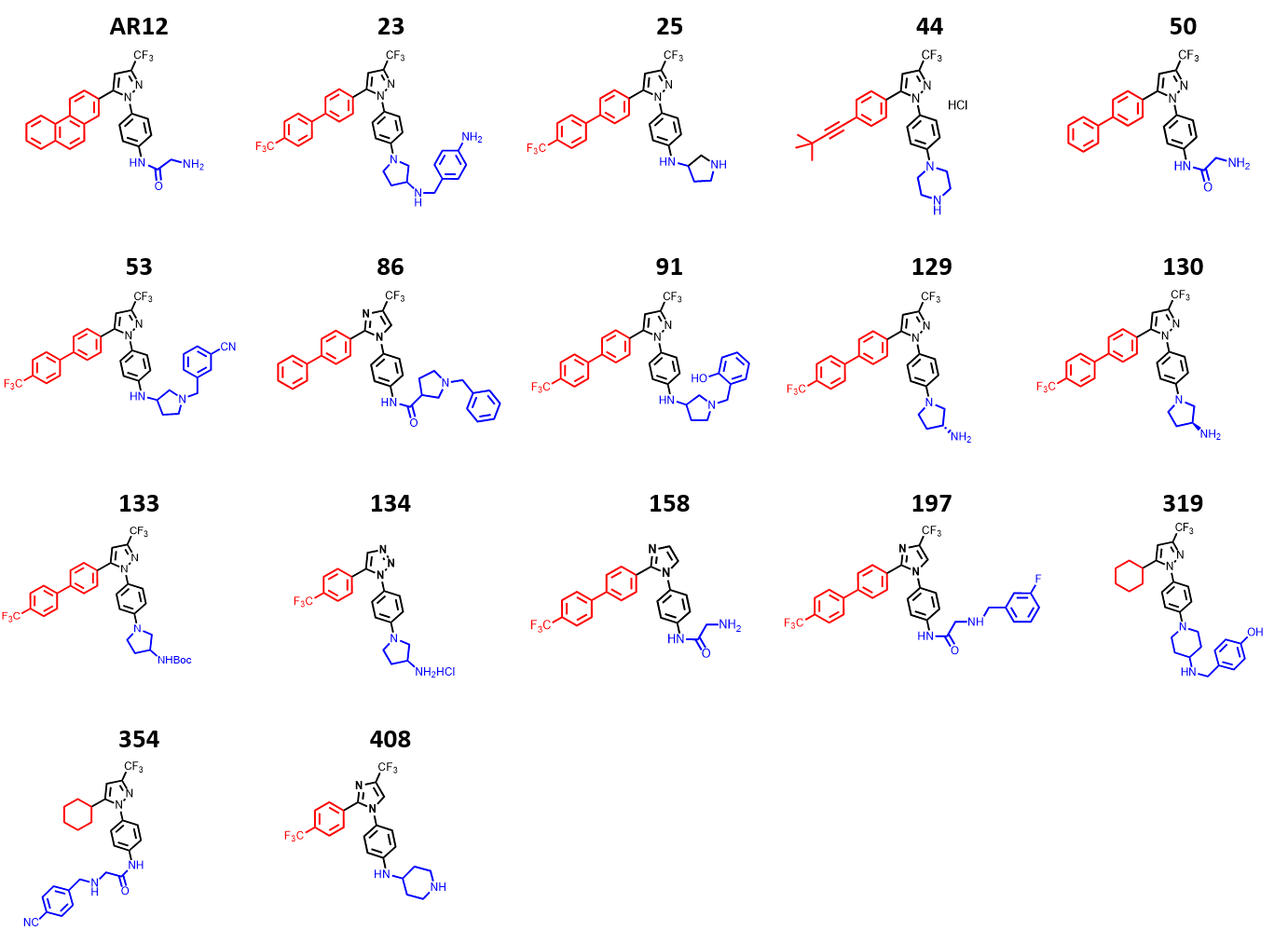


**S3 Fig.** Chemical structures of 16 hit compounds derived from AR-12 with associated core, R1, and R2 modifications highlighted.
