# Supplementary material for: Synthesis and preliminary evaluation of novel compounds that demonstrate broad host-directed anti-leishmanial activity
Source: PLoS Negl Trop Dis. 2026 Jul 13;20(7):e0014520. doi: 10.1371/journal.pntd.0014520 (PMC13379085; doi:10.1371/journal.pntd.0014520)
Supplement: S6 Fig — (DOCX) [file pntd.0014520.s008.docx]

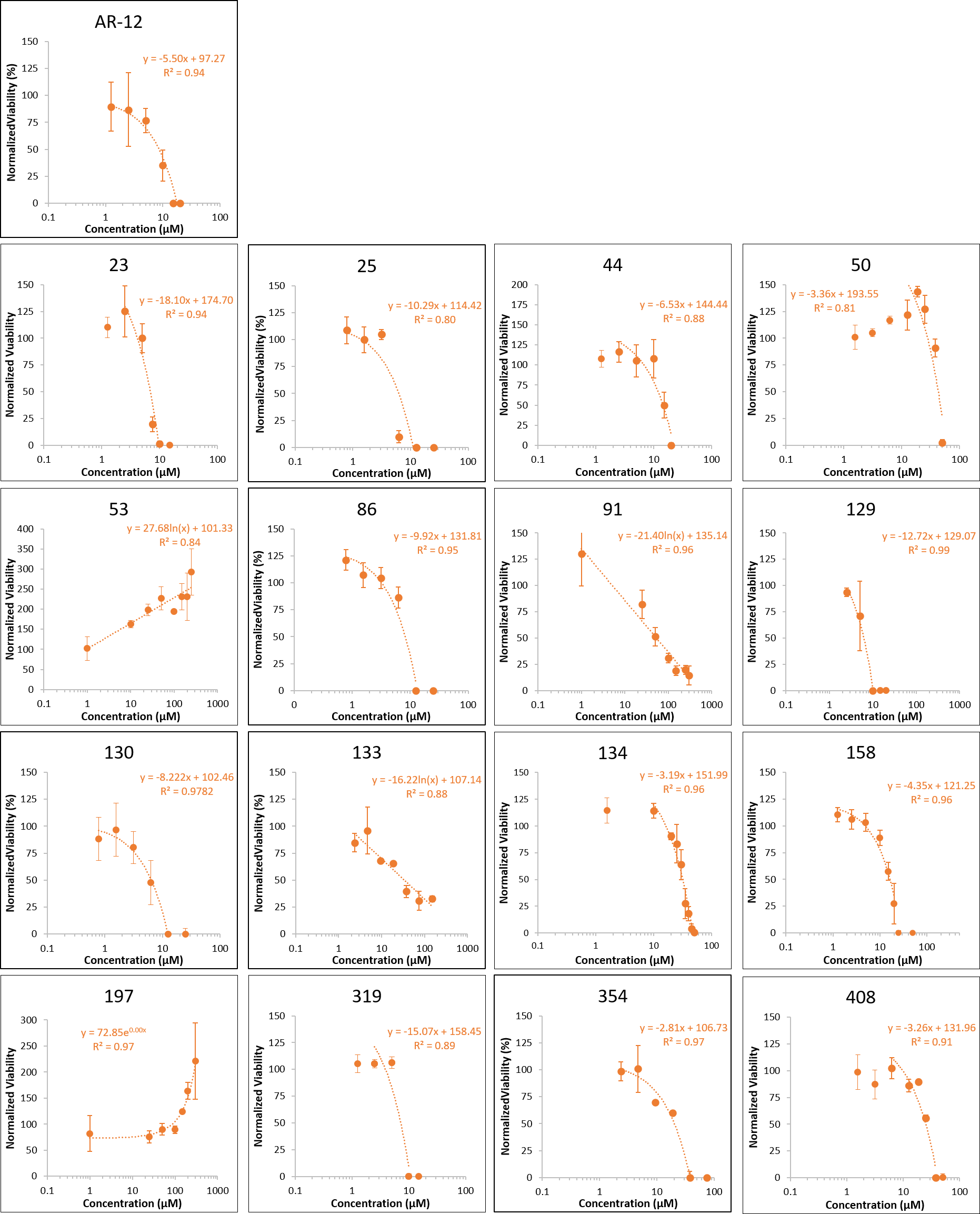


**S6 Fig.** Graphs of THP1 macrophage cell viability after 72-hour incubation with compounds as determined by MTT assay.
