# Supplementary material for: Synthesis and preliminary evaluation of novel compounds that demonstrate broad host-directed anti-leishmanial activity
Source: PLoS Negl Trop Dis. 2026 Jul 13;20(7):e0014520. doi: 10.1371/journal.pntd.0014520 (PMC13379085; doi:10.1371/journal.pntd.0014520)
Supplement: S7 Fig — (DOCX) [file pntd.0014520.s009.docx]

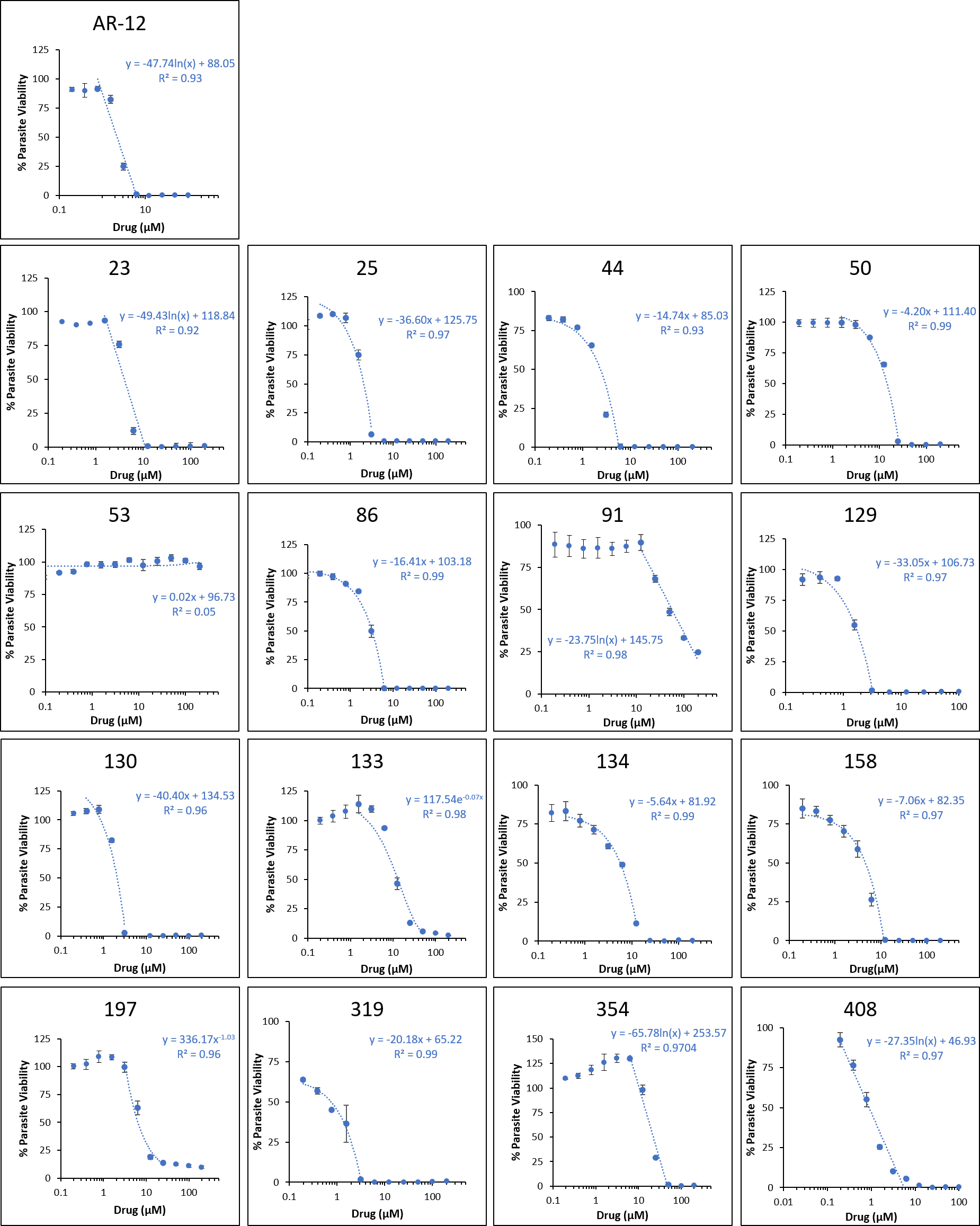


**S7 Fig.** Dose response of extracellular *Leishmania* promastigote viability after 72-hour incubation with compound as measured by resazurin assay.
