# Supplementary material for: Synthesis and preliminary evaluation of novel compounds that demonstrate broad host-directed anti-leishmanial activity
Source: PLoS Negl Trop Dis. 2026 Jul 13;20(7):e0014520. doi: 10.1371/journal.pntd.0014520 (PMC13379085; doi:10.1371/journal.pntd.0014520)
Supplement: S9 Fig — B) Luminescent activity of intracellular L. donovani infected THP1 macrophage cell after 72-hour incubation with 197 compared to chemically modified 197. (DOCX) [file pntd.0014520.s011.docx]

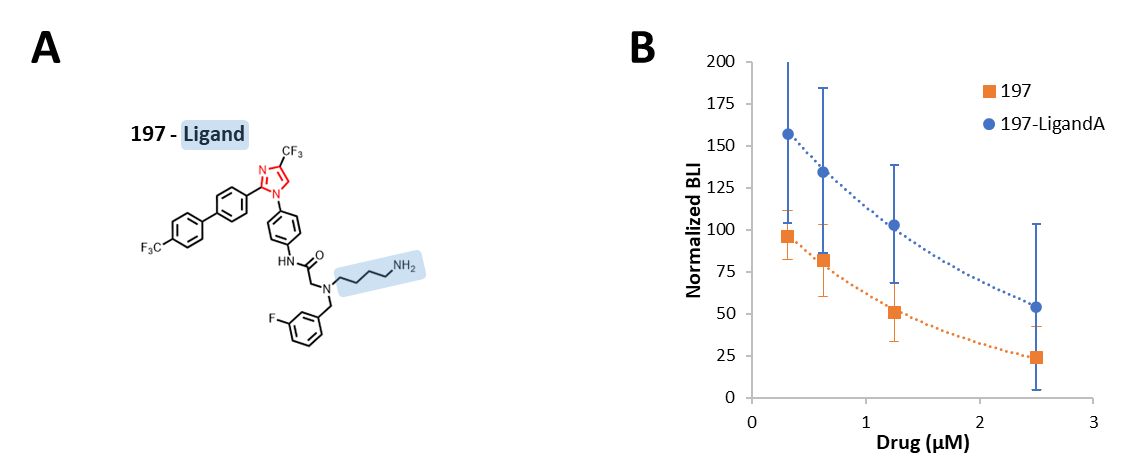


**S9 Fig. A)** Chemical structure of 197 chemically modified for conjugation to agarose bead for affinity capture proteomic analysis. **B)** Luminescent activity of intracellular *L. donovani* infected THP1 macrophage cell after 72-hour incubation with 197 compared to chemically modified 197.
