# Supplementary material for: Synthesis and preliminary evaluation of novel compounds that demonstrate broad host-directed anti-leishmanial activity
Source: PLoS Negl Trop Dis. 2026 Jul 13;20(7):e0014520. doi: 10.1371/journal.pntd.0014520 (PMC13379085; doi:10.1371/journal.pntd.0014520)
Supplement: S10 Fig — Significant proteins Log2FC > 1 and p < 0.05 are shown with black circles. B) All human proteins (gray circle) identified by thermal profile analysis with 197 plotted z-score and -Log10(p-value). Significant proteins with a |z-score| > 1 and p < 0.05 are shown with black circles. C) All Leishmania proteins (gray circle) identified by affinity capture using 197 functionalized bead plotted Log2(fold-change) and -Log10(p-value) over control bead. Significant proteins Log2FC > 1 and p < 0.05 are shown with black circles. Proteins with a (|z-score| > 1 and p < 0.05 overlapping with significant proteins identified by affinity capture (Log2FC > 2 and p < 0.05) are shown with open circles. D) All Leishmania proteins (gray circle) identified by thermal profile analysis with 197 plotted z-score and -Log10(p-value). Significant proteins with a |z-score| > 1 and p < 0.05 are shown with black circles. Significant proteins Log2FC > 1 and p < 0.05 are shown with black circles. Proteins with a (|z-score| > 1 and p < 0.05 overlapping with significant proteins identified by affinity capture (Log2FC > 2 and p < 0.05) are shown with open circles. E) Values for three Leishmania proteins overlapping between two proteomic approaches. (DOCX) [file pntd.0014520.s012.docx]

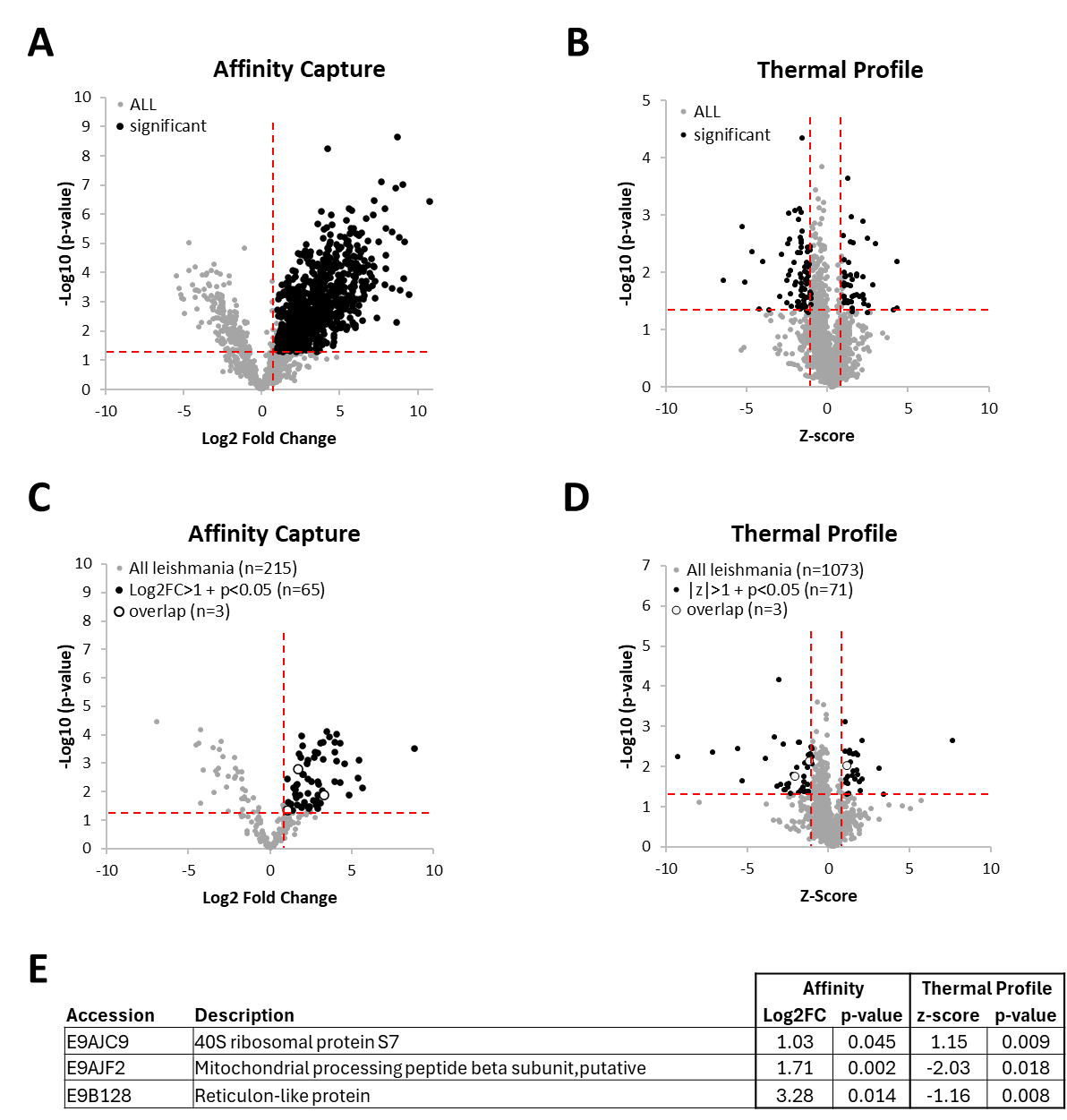


**S10 Fig. A)** All human proteins (gray circle) identified by affinity capture using 197 functionalized bead plotted Log2(fold-change) and -Log10(p-value) over control bead. Significant proteins Log2FC > 1 and p< 0.05 are shown with black circles. **B)** All human proteins (gray circle) identified by thermal profile analysis with 197 plotted z-score and -Log10(p-value). Significant proteins with a |z-score| > 1 and p< 0.05 are shown with black circles. **C)** All *Leishmania* proteins (gray circle) identified by affinity capture using 197 functionalized bead plotted Log2(fold-change) and -Log10(p-value) over control bead. Significant proteins Log2FC > 1 and p< 0.05 are shown with black circles. Proteins with a (|z-score| > 1 and p< 0.05 overlapping with significant proteins identified by affinity capture (Log2FC > 2 and p< 0.05) are shown with open circles. **D)** All *Leishmania* proteins (gray circle) identified by thermal profile analysis with 197 plotted z-score and -Log10(p-value). Significant proteins with a |z-score| > 1 and p< 0.05 are shown with black circles. Significant proteins Log2FC > 1 and p< 0.05 are shown with black circles. Proteins with a (|z-score| > 1 and p< 0.05 overlapping with significant proteins identified by affinity capture (Log2FC > 2 and p< 0.05) are shown with open circles. **E)** Values for three *Leishmania* proteins overlapping between two proteomic approaches.
