# Supplementary material for: Synthesis and preliminary evaluation of novel compounds that demonstrate broad host-directed anti-leishmanial activity
Source: PLoS Negl Trop Dis. 2026 Jul 13;20(7):e0014520. doi: 10.1371/journal.pntd.0014520 (PMC13379085; doi:10.1371/journal.pntd.0014520)
Supplement: S11 Fig — Data is presented as mean ± standard deviation of biological triplicates. (DOCX) [file pntd.0014520.s013.docx]

**
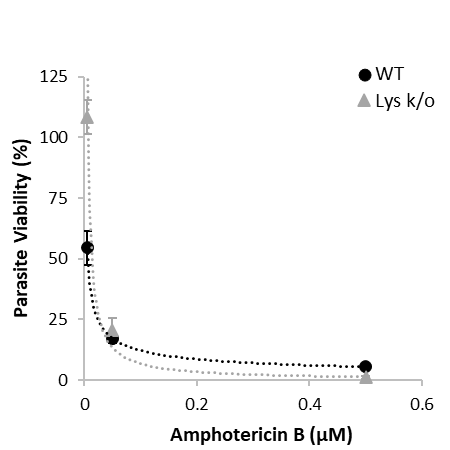
**

**Supplemental Figure 11.** Dose response of amphotericin B on intracellular *Leishmania* burden in bone marrow derived macrophages derived from wildtype C57BL/6 (WT, black circle) or lysozyme knockout mice (Lys K/O, gray triangle) as identified image-based Giemsa staining. Data is presented as mean ± standard deviation of biological triplicates.
