# Supplementary material for: Synthesis and preliminary evaluation of novel compounds that demonstrate broad host-directed anti-leishmanial activity
Source: PLoS Negl Trop Dis. 2026 Jul 13;20(7):e0014520. doi: 10.1371/journal.pntd.0014520 (PMC13379085; doi:10.1371/journal.pntd.0014520)
Supplement: S1 File — (DOCX) [file pntd.0014520.s014.docx]

**Synthesis and Characterization of Selected Analogs:**

Seven compounds were commercially available and purchased for this project. **121** (PRE-084 hydrochloride), **122** (SA4503 dihydrochloride), **123** (NE 100 hydrochloride), and **124** (BD 1047 dihydrobromide) were purchased from Tocris Bioscience, Inc. **125** (haloperidol) was acquired from Alfa Aesar. **278** ((S)-2-(3-fluorophenyl)pyrrolidine d-Tartrate) was sourced from AstaTech Inc. Lastly, **435** (notoginsenoside R1) was purchased from Sigma-Aldrich.


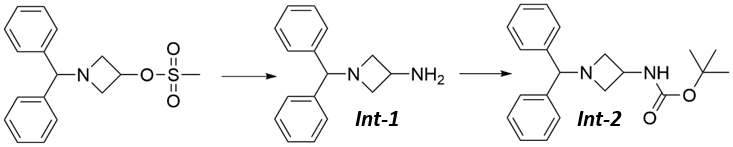


**11:** In a glass pressure reactor was combined 1-Benzhydrylazetidin-3-yl methanesulfonate (5.07 g, 15.9 mmol) and NH_4_OH (19.5 mL, 288.6 mmol) in 2-propanol (30 mL). The mixture was sealed with a Teflon cap and heated to 70^o^ C for 3 hours. The reaction mixture was quenched with saturated aqueous NaHCO_3_ and extracted with ethyl acetate. The organic layer was washed with brine, dried (Na_2_SO_4_), filtered and concentrated to yield 3.76 g (100%) of ***int-1*** as an off-white gel, which required no further purification. ^1^H-NMR (CDCl_3_) δ 7.40-7.27 (m, 4 H), 7.24-7.15 (m, 6 H), 4.27 (s, 1 H), 3.67-3.50 (m, 3 H), 2.73-2.64 (m, 2 H).

A mixture containing ***int-1*** (3.76 g, 15.8 mmol) in tetrahydrofuran (75 mL) and 5% aqueous Na_2_CO_3_ (90 mL) was cooled to 0^o^ C. A solution of di-tert-butyl decarbonate (4.48 g, 20.5 mmol) in tetrahydrofuran (15 mL) was added slowly and the reaction stirred at room temperature for 18 hours. Upon completion, the solvents were removed *in vacuo* and the residue was extracted with ethyl acetate. The organic layer was washed with brine, dried (MgSO_4_), filtered and concentrated to yield 6.03 g of a white solid. The crude material was stirred in hexanes and the resulting solid was filtered, washed with hexanes and dried to obtain 3.91 g (73%) of ***int-2*** (**11**) as a white solid.  ^1^H-NMR (CDCl_3_) δ 7.39 (m, 4 H), 7.29-7.15 (m, 6 H), 4.86 (br s, 1 H), 4.32-4.27 (m, 2 H), 3.52 (t, 2 H, J = 9.0 Hz), 2.87-2.79 (m, 2 H), 1.42 (s, 9 H). LC-MS, calculated for C_21_H_26_N_2_O_2_ (MH)^+^ 339.4; observed 339.2.


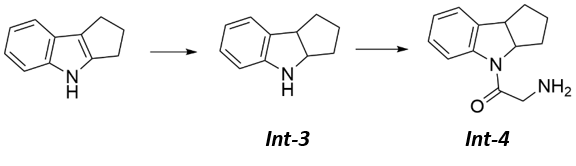


**320:** 1,2,3,4-Tetrahydrocyclopent[b]indole (4.0 g, 25.4 mmol) and 10% Palladium on Carbon (500 mg) were suspended in ethanol (50 mL) and concentrated hydrochloride acid (2.7 mL), charged with hydrogen gas and agitated on a Parr Apparatus at room temperature for 18 hours. The mixture was filtered through Celite and the pad was washed with methanol. The filtrate was concentrated, diluted with 1 N hydrochloric acid and extracted with diethyl ether. The aqueous layer was neutralized to pH of 8 with 2 N aqueous NaOH and extracted with dichloromethane. The organic layer was washed with brine, dried (MgSO_4_), filtered and concentrated to yield 3.05 g (75%) of **3** as a brown oil, which required no further purification. ^1^H-NMR (CDCl_3_) δ 7.02 (d, 1 H, J = 6.0 Hz), 6.98 (d, 1 H, J = 6.0 Hz), 6.67 (dd, 1 H, J = 3.0 Hz, 6.0 Hz), 6.52 (d, 1 H, J = 9.0 Hz), 4.36 (dd, 1 H, J = 3.0 Hz, 6.0 Hz), 3.77 (t, 2 H, J = 9.0 Hz), 2.02-1.51 (m, 6 H).

A mixture containing ***int-3*** (3.05 g, 19.15 mmol), 2-chloroacetamide (3.71 g, 39.6 mmol) and diisopropylethylamine (10.3 mL, 59.1 mmol) in N, N-dimethylformamide (8 mL), in a glass pressure reactor, was sealed with a Teflon cap and heated to 100^o^ C for 18 hours. The mixture was diluted with water and extracted with ethyl acetate. The organic layer was washed with brine, dried (MgSO_4_), filtered and concentrated. The crude material was adsorbed onto Celite and purified over silica gel using 0-50% hexane/ethyl acetate yielding 3.74 g (86%) of ***int-4*** (**320**) as a yellow solid. ^1^H-NMR (CDCl_3_) δ 7.07 (dd, 2 H, J = 6.0 Hz), 6.74 (dd, 1 H, J = 6.0 Hz, 9.0 Hz), 6.50 (br s, 1 H), 6.34 (d, 1 H, J = 9.0 Hz), 5.49 (br s, 1 H), 4.20-4.05 (m, 2 H), 3.78 (dd, 2 H, J = 9.0 Hz), 2.04-1.49 (m, 6 H). LC-MS, calculated for C_13_H_16_N_2_O (MH)^+^ 217.3; observed 217.0. Anal. Calculated for C_13_H_16_N_2_O: C, 72.19; H, 7.45; N, 12.95. Found: C, 71.93; H, 7.43; N, 12.90.

Novel Pyrazole Scaffolds (Type A: Standard Structure with N-substituted Anilines):


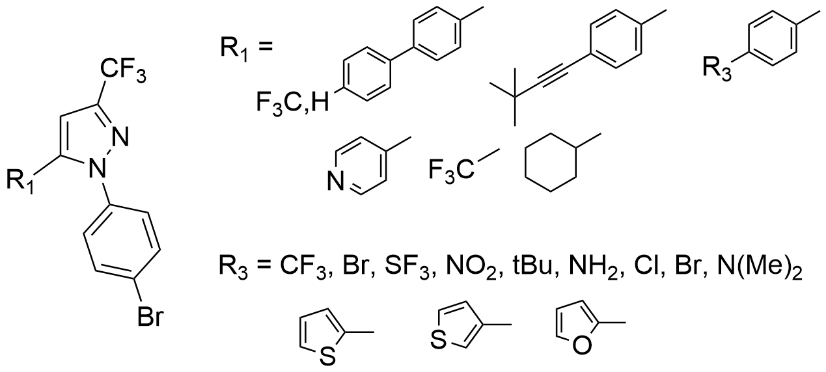


Examples:


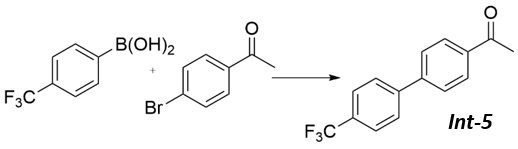


***int-5***: A mixture of 4-Trifluoromethylphenylboronic acid (7.84 g, 41.3 mmol), 4-Bromoacetophenone (8.25 g, 41.4 mmol), Palladium acetate (186 mg, 0.82 mmol), K_2_CO_3_ (17.1 g, 123.8 mmol) and Tetrabutylammonium bromide (17.3 g, 53.6 mmol) in THF (40 mL) and nitrogen gas was bubbled into the mixture for two minutes. Water (410 mL) was added and the reaction was heated to 60 C for 2 hours. Upon cooling to room temperature, the mixture was extracted with ethyl acetate. The organic material was washed with water and brine, dried (MgSO_4_), filtered and concentrated to yield a quantitative yield (11.2 g) of a copper-colored solid (***int-5***), which was pure enough for further synthesis. ^1^H-NMR (CDCl_3_) δ 8.05 (d, 2 H, J = 6 Hz), 7.79-7.69 (m, 6 H), 2.68 (s, 3 H).


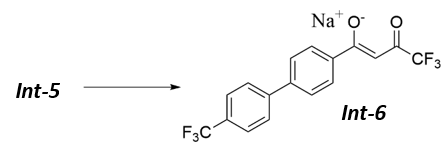


***int-6***: Into an oven dried flask was introduced sodium hydride (60% wt./mineral oil, 6.54 g, 272.4 mmol) and stirred in anhydrous THF (50 mL) for 5 minutes at room temperature. Ethyl trifluoroacetate (21.7 mL, 182.4 mmol) was added dropwise and this mixture stirred at room temperature for 10 minutes. A solution of ***int-5*** (24 g, 90.8 mmol) in anhydrous THF (85 mL) was added dropwise and the reaction mixture was refluxed for 3 hours. The reaction was concentrated and the residue was partitioned between ethyl acetate and water. The aqueous layer was extracted with ethyl acetate and the combined organics were washed with brine, dried (Na_2_SO_4_) and concentrated to yield a quantitative yield of a yellow solid (***int-6***), which was pure enough for the next step. ^1^H-NMR (CDCl_3_) δ 7.70 (d, 2 H, J = 6 Hz), 7.63 (d, 2 H, J = 9 Hz), 7.50 (d, 2 H, J = 9 Hz), 7.36 (d, 2 H, J = 9 Hz), 6.08 (s, 1 H).


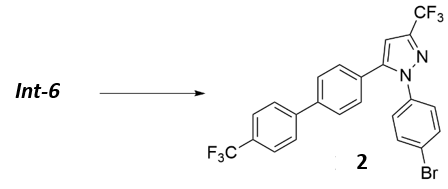


**2:** A mixture containing ***int-6*** (20 g, 52.3 mmol) and 4-bromohydrazine hydrochloride (16.4 g, 73.4 mmol) in ethanol (650 mL) was refluxed for 18 hours. The solvent was concentrated and the residue was partitioned between ethyl acetate and saturated aqueous NaHCO_3_. The aqueous layer was extracted with ethyl acetate and the combined organics were washed with brine, dried (MgSO_4_), filtered and concentrated. The crude material was purified over silica gel using 1-5% ethyl acetate from hexanes to yield 18.7 g of a yellow solid that contained both pyrazole isomers. The solid was crystallized from DCM/hexanes to yield 6.9 g (26%) of pure **2** as a yellow solid. ^1^H-NMR (CDCl_3_) δ 7.71 (dd, 4 H, J = 9 Hz), 7.60 (d, 2 H, J = 6 Hz), 7.53 (d, 2 H, J = 9 Hz), 7.31 (d, 2 H, J = 6 Hz), 7.25 (d, 2 H, J = 9 Hz), 6.81 (s, 1 H). LC-MS, calculated for C_23_H_13_BrF_6_N_2_ (MH)^+^ 512.2; observed 513.0.


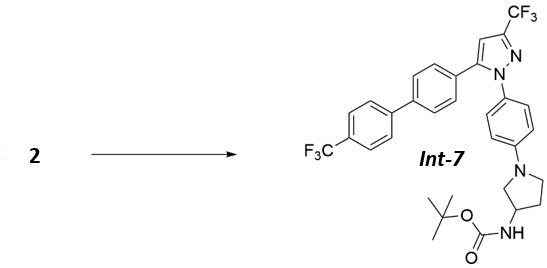


***int-7*:** The following were combined in a heavy-duty glass reactor: **2** (4.65 g, 9.10 mmol), 3-N-Boc-aminopyrrolidine (2.72 g, 14.5 mmol), BINAP (1.7 g, 2.73 mmol), Pd_2_(dba)_3_ (1.08 g, 1.18 mmol) and Cs_2_CO_3_ (4.75 g, 14.5 mmol) in anhydrous toluene (95 mL) and nitrogen gas was bubbled into the mixture for two minutes. The reactor was then sealed with a Teflon cap and heated to 110^o^ C for 18 hours. Upon cooling, the mixture was filtered through Celite and the filter pad was rinsed with ethyl acetate. The filtrate was washed with water and brine, dried (Na_2_SO_4_), filtered and concentrated. The crude material was purified over silica gel using 0-10% ethyl acetate from hexanes to yield 4.6 g (80%) of a yellow solid (***int-7***). ^1^H-NMR (CDCl_3_) δ 7.71 (dd, 4 H, J = 9 Hz), 7.56 (d, 2 H, J = 6 Hz), 7.35 (d, 2 H, J = 9 Hz), 7.18 (d, 2 H, J = 9 Hz), 6.78 (s, 1 H), 6.49 (d, 2 H, J = 9 Hz), 4.78-4.68 (m, 1 H), 4.42-4.33 (m, 1 H), 3.59 (dd, 1 H, J = 3 Hz, 6 Hz), 3.47-3.32 (m, 2 H), 3.17 (dd, 1 H, J = 3 Hz, 6 Hz), 2.33-2.26 (m, 1 H), 2.05-1.94 (m, 1 H), 1.46 (s, 9 H).


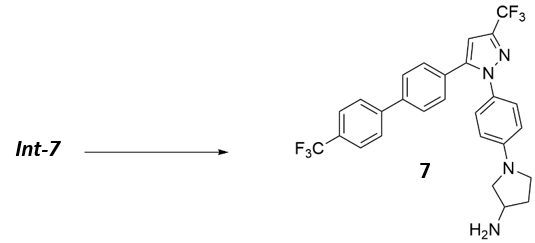


**7:** A solution of ***int-7*** (1.68 g, 2.72 mmol) in dichloromethane (30 mL) was cooled to 0^o^ C and treated with trifluoroacetic acid (2.0 mL, 26.9 mmol). The reaction warmed to room temperature and stirred for 18 hours. Upon completion, the mixture was concentrated and the residue was partitioned between ethyl acetate and 2 N aqueous NaOH. The aqueous layer was extracted with ethyl acetate and the combined organic layers were washed with brine, dried (Na_2_SO_4_), filtered and concentrated to yield a tan solid (**7**, 1.34 g, 94%) that required no further purification. ^1^H-NMR (CDCl_3_) δ 7.66 (s, 4 H), 7.56 (d, 2 H, J = 9 Hz), 7.35 (d, 2 H, J = 9 Hz), 7.17 (d, 2 H, J = 9 Hz), 6.78 (s, 1 H), 6.49 (d, 2 H, J = 9 Hz), 3.75 (dd, 1 H, J = 6 Hz), 3.55-3.45 (m, 2 H), 3.35 (dd, 1 H, J = 6 Hz), 3.04 (dd, 1 H, J = 3 Hz, 6 Hz), 2.29-2.18 (m, 1 H), 1.88-1.78 (m, 1 H). ESI-MS, calculated for C_27_H_22_F_6_N_4_ (MH)^+^ 517.4; observed 517.6; Anal. Calculated for C_27_H_22_F_6_N_4_; C, 62.79; H, 4.29; N, 10.84. Found: C, 62.72; H, 4.29; N, 10.59.

**129 (5b: R-amino orientation):** The product was isolated as a brown gel in 59 % yield (160 mg). ^1^H-NMR (CDCl_3_) δ 7.69 (s, 4 H), 7.56 (d, 2 H, J = 9 Hz), 7.35 (d, 2 H, J = 9 Hz), 7.17 (d, 2 H, J = 9 Hz), 6.78 (s, 1 H), 6.49 (d, 2 H, J = 9 Hz), 3.76 (dd, 1 H, J = 3 Hz, 6 Hz), 3.55-3.45 (m, 2 H), 3.38-3.30 (m, 1 H), 3.04 (dd, 1 H, J = 3 Hz, 6 Hz), 2.29-2.18 (m, 1 H), 1.88-1.78 (m, 1 H). ^13^C-NMR (CDCl_3_, 75 MHz) δ 147.7, 143.7, 139.7, 129.2, 127.4, 127.3, 126.7, 125.9, 111.4, 104.9, 56.4, 51.4, 46.3, 34.9. ESI-MS, calculated for C_27_H_22_F_6_N_4_ (MH)^+^ 517.4; observed 517.6; Anal. Calculated for C_27_H_22_F_6_N_4_; C, 62.79; H, 4.29; N, 10.84. Found: C, 62.69; H, 4.37; N, 10.55; [α] = + 4.28 (c = 0.70/CHCl_3_).

**130 (5c: S-amino orientation):** The product was isolated as a tan solid in 82 % yield (335 mg). ^1^H-NMR (CDCl_3_) δ 7.66 (s, 4 H), 7.56 (d, 2 H, J = 9 Hz), 7.35 (d, 2 H, J = 9 Hz), 7.17 (d, 2 H, J = 9 Hz), 6.78 (s, 1 H), 6.49 (d, 2 H, J = 9 Hz), 3.75 (dd, 1 H, J = 6 Hz), 3.55-3.45 (m, 2 H), 3.38-3.30 (dd, 1 H, J = 6 Hz, 9 Hz), 3.04 (dd, 1 H, J = 3 Hz, 6 Hz), 2.29-2.18 (m, 1 H), 1.88-1.78 (m, 1 H). ESI-MS, calculated for C_27_H_22_F_6_N_4_ (MH)^+^ 517.4; observed 517.6; Anal. Calculated (with 0.2 mol water) for C_27_H_22_F_6_N_4_; C, 62.35; H, 4.34; N, 10.77. Found: C, 62.16; H, 4.37; N, 10.65; [α] = - 2.50 (c = 0.80/CHCl_3_).


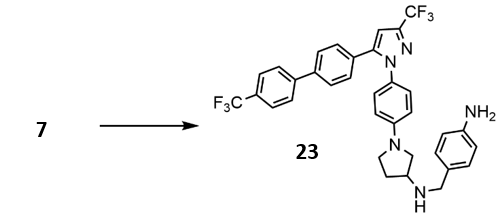


**23:** A solution of **7** (150 mg, 0.29 mmol), 4-nitrobenzaldehyde (44 mg, 0.29 mmol) and 4A molecular sieves (150 mg) in anhydrous methanol (3 mL) and anhydrous tetrahydrofuran (1.5 mL) was stirred at room temperature for 18 hours. The reaction was cooled to 0^o^ C and treated with sodium borohydride (22 mg, 0.58 mmol) was added and the reaction stirred for 4 hours at room temperature. The reaction was concentrated and the residue partitioned between saturated aqueous sodium bicarbonate solution and ethyl acetate. The combined organic layers were washed with brine, dried (Na_2_SO_4_), filtered and concentrated to yield 203.6 mg of a brown gel. The crude material was purified over silica gel using 0-10 % methanol from dichloromethane to yield 65 mg (36%) of **23** as an off-white solid. ^1^H-NMR (CDCl_3_) δ 7.69 (s, 4 H), 7.56 (d, 2 H, J = 9 Hz), 7.34 (d, 2 H, J = 6 Hz), 7.14 (dd, 4 H, J = 3 Hz, 9 Hz), 6.77 (s, 1 H), 6.66 (d, 2 H, J = 6 Hz), 6.47 (d, 2 H, J = 9 Hz), 3.76 (s, 2 H), 3.63-3.41 (m, 6 H), 3.39-3.29 (m, 1 H), 3.19-3.08 (m, 1 H), 2.31-2.19 (m, 1 H), 1.99-1.88 (m, 1 H). ESI-MS, calculated for C_34_H_27_F_6_N_5_O_2_ (MH)^+^ 622.6; observed 622.3.

Other Selected Analogs:


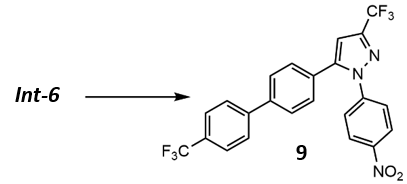


**9:** A mixture containing ***int-6*** (19.16 g, 50.1 mmol) and 4-nitrohydrazine hydrochloride (12.35 g, 65.1 mmol) in ethanol (600 mL) was refluxed for 18 hours. The solvent was concentrated and the residue was partitioned between ethyl acetate and saturated aqueous NaHCO_3_. The aqueous layer was extracted with ethyl acetate and the combined organics were washed with brine, dried (MgSO_4_), filtered and concentrated. The crude material was purified over silica gel using 1-5% ethyl acetate from hexanes to yield 10.58 g (44%) of pure **9** as a yellow solid. ^1^H-NMR (CDCl_3_) δ 8.27 (d, 2 H, J = 6 Hz), 7.75-7.69 (m, 4 H), 7.64 (d, 2 H, J = 9 Hz), 7.57 (d, 2 H, J = 6 Hz), 7.36 (d, 2 H, J = 9 Hz), 6.85 (s, 1 H). ESI-MS, calculated for C_23_H_13_F_6_N_3_O_2_ (MH)^+^ 477.4; observed 477.8; Anal. Calculated for C_23_H_13_F_6_N_3_O_2_; C, 57.87; H, 2.74; N, 8.80. Found: C, 57.61; H, 2.92; N, 8.71.


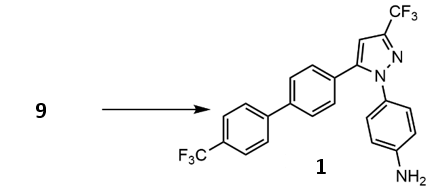


**1:** A mixture containing **9** (8.93 g, 18.7 mmol), Tin (II) chloride (12.4 g, 65.5 mmol) and concentrated hydrochloric acid (28 mL, 336 mmol) in ethanol (125 mL) was heated to 50 ^o^C for 2.5 hours. The solvent was concentrated and the residue was partitioned between ethyl acetate and 2 N NaOH. The aqueous layer was extracted with ethyl acetate and the combined organics were washed with water and brine, dried (MgSO_4_), filtered and concentrated to yield 8.15 g (97 %) of **1** as a tan solid, which was used without any further purification. ^1^H-NMR (CDCl_3_) δ 7.68 (dd, 4 H, J = 9 Hz), 7.56 (d, 2 H, J = 9 Hz), 7.35 (d, 2 H, J = 9 Hz), 7.13 (d, 2 H, J = 9 Hz), 6.78 (s, 1 H), 6.66 (d, 2 H, J = 9 Hz), 3.82 (br s, 2 H). LC-MS, calculated for C23H15F6N3 (MH)+ 448.3; observed 448.0.


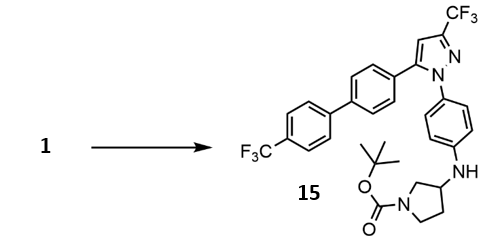


**15:** A mixture of **1** (6 g, 13.4 mmol) and N-Boc-3-pyrrolidinone (3.23 g, 17.4 mmol) in glacial acetic acid (110 mL) was treated with anhydrous sodium sulfate (11.43 g, 80.4 mmol); the mixture was then cooled to 0^o^ C. Sodium triacetoxyborohydride (5.97 g, 28.2 mmol) was added and the reaction was stirred at room temperature for 18 hours. Upon completion, the reaction was concentrated and the residue was partitioned between ethyl acetate and saturated aqueous sodium bicarbonate. The aqueous layer was extracted with ethyl acetate. The organic layer was washed with brine, dried (Na_2_SO_4_), filtered and concentrated to yield 10.58 g of a black gel. The crude material was purified over silica gel using 0-20 % ethyl acetate from hexanes to yield 5.18 g (63 %) of **15** as a tan solid. ^1^H-NMR (CDCl_3_) δ 7.69 (s, 4 H), 7.60 (d, 2 H, J = 9 Hz), 7.36 (d, 2 H, J = 9 Hz), 7.16 (d, 2 H, J = 9 Hz), 6.78 (s, 1 H), 6.57 (d, 2 H, J = 9 Hz), 4.10-4.00 (m, 1 H), 3.98-3.90 (m, 1 H), 3.78-3.65 (m, 1 H), 3.56-3.45 (m, 2 H), 3.37-3.19 (m 1 H), 2.26-2.18 (m,1 H), 1.97-1.86 (m, 1 H), 1.46 (s, 9 H). ESI-MS, calculated for C_32_H_30_F_6_N_4_O_2_ (M+Na)^+^ 639.6; observed 639.5.


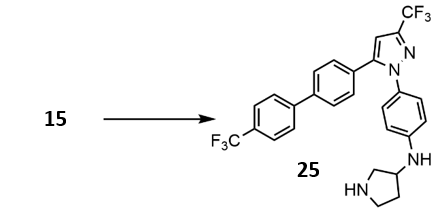


**25:** A solution of **15** (4.90 g, 7.96 mmol) in dichloromethane (55 mL) was cooled to 0^o^ C and treated with trifluoroacetic acid (5.9 mL, 79.4 mmol). The reaction warmed to room temperature and stirred for 18 hours. Upon completion, the mixture was concentrated and the residue was partitioned between ethyl acetate and 2 N aqueous NaOH. The aqueous layer was extracted with ethyl acetate and the combined organic layers were washed with brine, dried (Na_2_SO_4_), filtered and concentrated to yield a brown solid (**25**, 4.0 g, 97%) that required no further purification. ^1^H-NMR (CDCl_3_) δ 7.68 (dd, 4 H, J = 9 Hz), 7.56 (d, 2 H, J = 9 Hz), 7.36 (d, 2 H, J = 9 Hz), 7.14 (d, 2 H, J = 9 Hz), 6.78 (s, 1 H), 6.56 (d, 2 H, J = 9 Hz), 4.05-3.91 (m, 2 H), 3.21-3.08 (m, 2 H), 3.01-2.93 (m, 1 H), 2.91-2.85 (m, 1 H), 2.27-2.15 (m, 1 H). LC-MS, calculated for C_27_H_22_F_6_N_4_ (MH)^+^ 517.4; observed 517.2. Anal. Calculated (with 0.8 mol of water) for C_27_H_22_F_6_N_4_; C, 61.08; H, 4.48; N, 10.55. Found: C, 61.23; H, 4.30; N, 10.43.


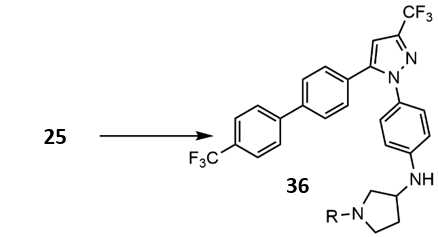


**36 [R = CH_2_-(4-biphenyl)]:** A solution of **25** (150 mg, 0.29 mmol), biphenyl 4-carboxaldehyde (58 mg, 0.32 mmol) and 4A molecular sieves (150 mg) in anhydrous 1,2-dichloroethane (4 mL) was stirred at room temperature for 5 hours. Sodium triacetoxyborohydride (136 mg, 0.64 mmol) was added and the reaction stirred for 18 hours at room temperature. The reaction was quenched with saturated aqueous sodium bicarbonate solution and extracted with ethyl acetate and the combined organic layers were washed with brine, dried (Na_2_SO_4_), filtered and concentrated to yield 176 mg of a brown solid. The crude material was purified over silica gel using 0-40 % ethyl acetate from hexanes to yield 85.7 mg (43%) of **36** as a white solid. ^1^H-NMR (CDCl_3_) δ 7.68 (dd, 4 H, J = 9 Hz), 7.60-7.53 (m, 6 H), 7.46-7.33 (m, 7 H), 7.12 (d, 2 H, J = 6 Hz), 6.77 (s, 1 H), 6.54 (d, 2 H, J = 9 Hz), 4.11 (dd, 1 H, J = 6 Hz), 4.01 (br s, 1 H), 3.68 (s, 2 H), 2.86-2.75 (m, 2 H), 2.62 (d, 1 H, J = 3 Hz, 6 Hz), 2.48 (dd, 1 H, J = 6 Hz, 9 Hz), 2.34 (dd, 1 H, J = 6 Hz, 9 Hz), 1.77-1.65 (m, 1 H). ESI-MS, calculated for C_40_H_32_F_6_N_4_ (MH)^+^ 683.7; observed 683.7.

**53 [R = CH_2_-(3-cyanophenyl)]:** Using 3-cyanobenzaldehyde, the product was isolated as a white solid in 58% yield (142 mg). ^1^H-NMR (CDCl_3_) δ 7.65 (dd, 5 H, J = 12 Hz), 7.55 (d, 2 H, J = 9 Hz), 7.43 (m, d, 1 H, J = 6 Hz), 7.35 (dd, 2 H, J = 9 Hz), 7.14 (dd, 2 H, J = 9 Hz), 6.77 (s, 1 H), 6.55 (d, 2 H, J = 9 Hz), 4.16-4.03 (m, 2 H), 3.65 (s, 2 H), 2.84-2.71 (m, 2 H), 2.59 (dd, 1 H, J = 9 Hz), 2.46-2.28 (m, 2 H), 1.76-1.65 (m, 1 H). ^13^C-NMR (CDCl_3_, 75 MHz) δ 147.6, 143.7, 140.4, 139.9, 133.0, 132.2, 130.9, 129.5, 129.2, 127.4, 126.9, 125.9, 118.9, 113.3, 112.5, 105.0, 60.8, 59.3, 52.8, 52.4, 32.6. ESI-MS, calculated for C_35_H_27_F_6_N_5_ (MH)^+^ 632.6; observed 632.6.

**91 [R = CH_2_-(2-hydroxyphenyl)]:** Using 2-hydroxybenzaldehyde, the product was isolated as an off-white solid in 48% yield (138.6 mg). ^1^H-NMR (CDCl_3_) δ 7.68 (dd, 4 H, J = 9 Hz), 7.56 (d, 2 H, J = 9 Hz), 7.35 (dd, 2 H, J = 9 Hz), 7.16 (dd, 3 H, J = 6 Hz, 9 Hz), 6.99 (d, 1 H, J = 9 Hz), 6.84-6.76 (m, 3 H), 6.53 (d, 2 H, J = 9 Hz), 4.06-3.97 (m, 2 H), 3.84 (s, 2 H), 2.97-2.85 (m, 2 H), 2.70 (dd, 1 H, J = 3 Hz, 6 Hz), 2.56 (dd, 1 H, J = 9 Hz), 2.42 (dd, 1 H, J = 6 Hz, 9 Hz), 1.78-1.72 (m, 1 H). ESI-MS, calculated for C_34_H_28_F_6_N_4_O (MH)^+^ 623.6; observed 623.8.

Preparation of Novel Pyrazole Scaffolds (Type B: Left-Ring Substitution with N-substituted Anilines):


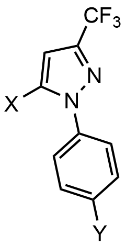


Following procedures described above, a few examples include:

**247 (X = Cyclohexyl, Y = 3-Aminopyrrolidinyl):** Using 1-Cyclohexylethanone in the procedure for **7**, **247** was isolated as a tan oil in 65% yield (125 mg). ^1^H-NMR (CDCl_3_) δ 7.26 (d, 2 H, J = 9 Hz), 6.54 (d, 2 H, J = 9 Hz), 6.53 (s, 1 H), 3.81-3.71 (m, 1 H), 3.61-3.49 (m, 2 H), 3.05 (dd, 1 H, J = 3 Hz, 6 Hz), 2.79-2.67 (m, 1 H), 2.32-2.21 (m, 1 H), 2.08-2.00 (m, 2 H), 1.91-1.71 (m, 4 H), 1.60-1.22 (m, 5 H). ESI-MS, calculated for C_20_H_25_F_3_N_4_ (MH)^+^ 379.4; observed 379.4; Anal. Calculated for C_20_H_25_F_3_N_4_; C, 63.47; H, 6.65; N, 14.80. Found: C, 63.31; H, 6.50; N, 14.57.

**272 (X = Cyclopropyl, Y *=* 3-Aminopyrrolidinyl)**: **272** was isolated as a tan solid (139.6 mg, 24%, over 2 steps). ^1^H-NMR (CDCl_3_) δ 7.34 (d, 2 H, J = 6 Hz), 6.64 (d, 2 H, J = 6 Hz), 6.14 (s, 1 H), 4.07-3.94 (m, 2 H), 3.19-3.07 (m, 2 H), 3.00-2.85 (m, 2 H), 2.28-2.14 (m, 1 H), 1.79-1.68 (m, 3 H), 1.02-0.95 (m, 2 H), 0.78-0.73 (m, 2 H). NMR (CDCl_3_, 75 MHz) δ 147.6, 129.2, 126.6, 112.9, 99.2, 54.0, 53.9, 45.8, 33.7, 8.8, 7.4. ESI-MS, calculated for C_17_H_19_F_3_N_4_ (MH)^+^ 337.4; observed 337.4. Anal. Calculated for C_17_H_19_F_3_N_4_; C, 60.70; H, 5.69; N, 16.65. Found: C, 60.49; H, 5.80; N, 16.50.

**273 (X = 2-Furanyl, Y *=* 3-Aminopyrrolidinyl)**: **273** was isolated as a tan solid (382 mg, 37%, over 2 steps). ^1^H-NMR (CDCl_3_) δ 7.42 (d, 2 H, J = 3 Hz), 7.20 (d, 2 H, J = 6 Hz), 6.87 (s, 1 H), 6.63 (d, 2 H, J = 6 Hz), 6.33 (d, 1 H, J = 3 Hz), 5.92 (d, 1 H, J = 3 Hz), 4.14-3.95 (m, 2 H), 3.22-3.08 (m, 2 H), 3.01-2.86 (m, 2 H), 2.29-2.19 (m, 1 H), 1.76-1.67 (m, 1 H). NMR (CDCl_3_, 75 MHz) δ 148.3, 143.6, 142.8, 136.4, 129.1, 127.4, 119.4, 112.9, 111.3, 109.3, 102.8, 53.9, 53.8, 45.8, 33.6. ESI-MS, calculated for C_18_H_17_F_3_N_4_O (MH)^+^ 363.3; observed 363.4. Anal. Calculated for C_18_H_17_F_3_N_4_O; C, 59.75; H, 4.73; N, 15.48. Found: C, 59.36; H, 4.84; N, 15.52.


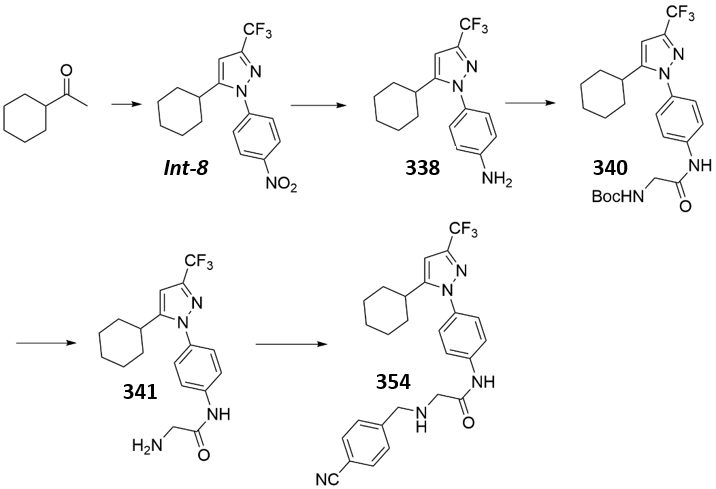


***int-8*:** Into an oven dried flask was introduced sodium hydride (60% wt./mineral oil, 9.52 g, 237.6 mmol) and stirred in anhydrous THF (140 mL) for 5 minutes at room temperature. Ethyl trifluoroacetate (18.9 mL, 158.8 mmol) was added dropwise and this mixture stirred at room temperature for 10 minutes. A solution of cyclohexyl methyl ketone (10 g, 79.2 mmol) in anhydrous THF (40 mL) was added dropwise and the reaction mixture was refluxed for 3 hours. The reaction was concentrated and the residue was partitioned between ethyl acetate and water. The aqueous layer was extracted with ethyl acetate and the combined organics were washed with brine, dried (Na_2_SO_4_) and concentrated to yield a quantitative yield of a yellow oil, which was used without further purification. ^1^H-NMR (CD_3_OD) δ 5.24 (s, 1 H), 2.94-2.84 (m, 1 H), 1.82-1.66 (m, 4 H), 1.40-1.19 (m, 6 H). Using this material, the procedure for **2** was followed, substituting 4-nitrohydrazine hydrochloride. During pyrazole formation, two different pyrazoles may form. The product (***int-8***, the less polar isomer, with approximate R_f_ = 0.7) was isolated as a yellow oil in 59% yield (15.9 g). ^1^H-NMR (CDCl_3_) δ 8.36 (d, 2 H, J = 9 Hz), 7.73 (d, 2 H, J = 9 Hz), 6.72 (s, 1 H), 2.77-2.69 (m, 1 H), 1.86-1.73 (m, 4 H), 1.59-1.22 (m, 6 H).

**338:** A mixture containing ***int-8*** (21.8 mmol), stannous chloride (14.5 g, 76.3 mmol) and concentrated HCl (33 mL, 396 mmol) in ethanol (145 mL) was heated to 50^o^ C for 2.5 hours. Upon cooling to room temperature, the solvent was concentrated and the residue was diluted with ethyl acetate and 2 N NaOH (300 mL) was stirred in the mixture stirred at room temperature for 1h. The aqueous layer was extracted with ethyl acetate and the combined organics were washed with water and brine, dried (MgSO_4_), filtered and concentrated. The crude material was adsorbed onto silica gel and purified via ISCO using 1-20% ethyl acetate from hexanes to yield 3.54 g (52%) of a white solid (**338**). ^1^H-NMR (CDCl_3_) δ 7.22 (d, 2 H, J = 9 Hz), 6.70 (d, 2 H, J = 9 Hz), 6.54 (s, 1 H), 3.83 (br s, 2 H), 2.74-2.65 (m, 1 H), 2.05-1.96 (m, 2 H), 1.84-1.71 (m, 2 H), 1.51-1.31 (m, 6 H). ^13^C NMR (CDCl_3_, 75 MHz) δ 147.1, 127.0, 114.7, 105.2, 60.3, 37.3, 33.0, 26.2, 26.0, 14.1; ESI-MS, calculated for C_16_H_18_F_3_N_3_ (MH)^+^ 310.3; observed 310.0. Anal. Calculated for C_16_H_18_F_3_N_3_; C, 62.12; H, 5.86; N, 13.58. Found: C, 62.06; H, 5.98; N, 13.44.

**340:** A mixture containing **338** (3.03 g, 9.79 mmol), Boc-glycine (3.77 g, 21.5 mmol), diisopropylethylamine (6.0 mL, 34.4 mmol), 4-N,N-dimethylaminopyridine (180 mg, 1.47 mmol) and N-(3-dimethylaminopropyl)-n-ethylcarbodiimide hydrochloride (4.13 g, 21.5 mmol) in tetrahydrofuran (90 mL) was stirred at room temperature for 18 hours. The solvent was concentrated and the residue was partitioned between ethyl acetate and 1 N HCl. The aqueous layer was extracted with ethyl acetate and the combined organics were washed with saturated aqueous NaHCO_3_, water and brine, dried (Na_2_SO_4_), filtered and concentrated. The crude material was adsorbed onto silica gel and purified via ISCO using 3-20% ethyl acetate from hexanes to yield 3.60 g (72%) of a white solid (**340**). ^1^H-NMR (CDCl_3_) δ 8.35 (br s, 1 H), 7.64 (d, 2 H, J = 9 Hz), 7.43 (d, 2 H, J = 9 Hz), 6.59 (s, 1 H), 5.22 (br s, 1 H), 3.94 (d, 2 H, J = 6 Hz), 2.71 (dd, 1 H, J = 3 Hz, 9 Hz), 2.07-2.00 (m, 2 H), 1.84-1.62 (m, 3 H), 1.49 (s, 9 H), 1.44-1.24 (m, 5 H). ^13^C NMR (CDCl_3_, 75 MHz) δ 126.3, 119.8, 37.3, 33.0, 28.2, 26.2, 26.0; ESI-MS, calculated for C_23_H_29_F_3_N_4_O_3_ (MH)^+^ 467.5; observed 467.0. Anal. Calculated for C_23_H_29_F_3_N_4_O_3_; C, 59.21; H, 6.26; N, 12.01. Found: C, 59.12; H, 6.26; N, 12.16.

**341:** A solution of **340** (3.36 g, 7.84 mmol) in dichloromethane (80 mL) was cooled to 0^o^ C and treated with trifluoroacetic acid (5.9 mL, 79.4 mmol). The reaction warmed to room temperature and stirred for 18 hours. Upon completion, the mixture was concentrated and the residue was partitioned between ethyl acetate and 2 N aqueous NaOH. The aqueous layer was extracted with ethyl acetate and the combined organic layers were washed with brine, dried (Na_2_SO_4_), filtered and concentrated to yield a tan solid. The crude material was purified over silica gel using 0-5% methanol from dichloromethane to yield 2.60 g (90%) of a white solid. Some of the crude material (400 mg) was adsorbed onto silica gel and purified via ISCO using 0-20% methanol from dichloromethane to yield 372 mg (93%) of a white solid (**341**). ^1^H-NMR (CDCl_3_) δ 9.59 (br s, 1 H), 7.73 (d, 2 H, J = 9 Hz), 7.44 (d, 2 H, J = 9 Hz), 6.59 (s, 1 H), 3.50 (s, 2 H), 2.72 (dd, 1 H, J = 3 Hz, 9 Hz), 2.08-1.96 (m, 2 H), 1.88-1.71 (m, 3 H), 1.52-1.33 (m, 5 H). ^13^C NMR (CDCl_3_, 75 MHz) δ 170.7, 158.6, 138.2, 136.5, 126.3, 119.3, 105.9, 45.1, 37.3, 33.0, 28.2, 26.2, 26.0; ESI-MS, calculated for C_18_H_21_F_3_N_4_O (MH)^+^ 367.3; observed 367.0. Anal. Calculated for C_18_H_21_F_3_N_4_O; C, 59.00; H, 5.77; N, 15.29. Found: C, 58.97; H, 5.81; N, 15.17.

**354:** A mixture containing **341** (300 mg, 0.82 mmol), 4-cyanobenzyl bromide (164 mg, 0.835 mmol) and triethylamine (0.29 mL, 2.08 mmol) in dimethylformamide (7 mL) was stirred for 18 hours at room temperature. The reaction was poured into a saturated aqueous LiCl solution and extracted with ethyl ether. The organic layer was washed with brine, dried (Na_2_SO_4_), filtered and concentrated. The crude material was purified over silica gel using 1-5% methanol from dichloromethane to yield 212 mg (54%) of a white solid (**354**). ^1^H-NMR (CDCl_3_) δ 9.19 (s, 1 H), 7.68 (d, 2 H, J = 9 Hz), 7.45 (dd, 2 H, J = 6 Hz, 9 Hz), 6.60 (s, 1 H), 3.95 (s, 2 H), 3.46 (s, 2 H), 2.72 (dd, 1 H, J 3 Hz, 6 Hz), 2.07-1.99 (m, 2 H), 1.85-1.72 (m, 5 H), 1.52-1.25 (m, 6 H). ^13^C NMR (CDCl_3_, 75 MHz) δ 169.0, 158.4, 144.2, 137.9, 135.2, 132.6, 128.6, 126.4, 119.4, 111.6, 106.0, 53.5, 52.4, 37.3, 33.0, 26.2, 25.9; ESI-MS, calculated for C_26_H_26_F_3_N_5_O (MH)^+^ 482.5; observed 482.0. Anal. Calculated for C_26_H_26_F_3_N_5_O; C, 64.85; H, 5.44; N, 14.54. Found: C, 64.56; H, 5.51; N, 14.48.

Examples of Imidazole Structure Synthesis:


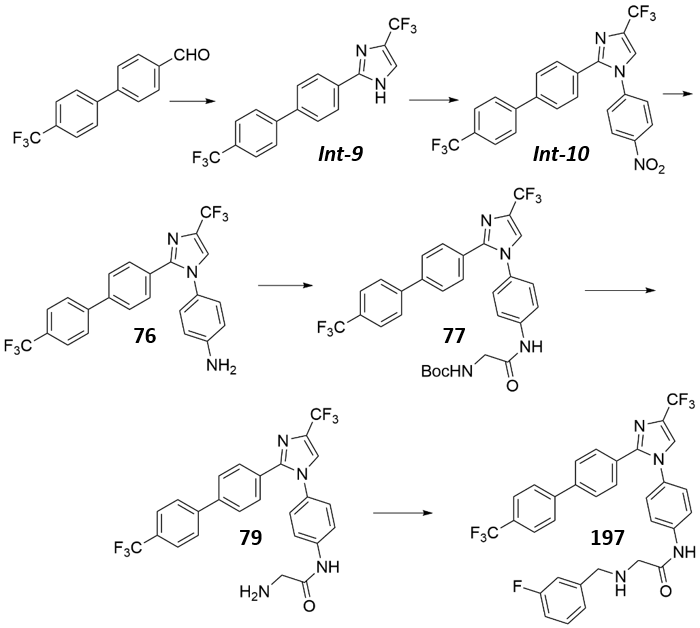


***int-9*:** A mixture containing 1,1-Dibromo-3,3,3-trifluoroacetone (3.3 g, 12.0 mmol) and sodium acetate (1.32 g, 16.0 mmol) in water (42 mL) was heated to 95^o^ C for 1 hour, then cooled to room temperature. 4’-Trifluoromethylbiphenyl-4-carboxaldehyde (2.0 g, 8.0 mmol) in methanol (102 mL) was cooled to 0^o^ C and treated with dropwise addition of ammonium hydroxide (49 mL, 725.2 mmol). This mixture stirred for 10 minutes at 0^o^ C and then the first mixture was added dropwise; the combined reaction slowly warmed to room temperature and stirred for 18 hours. The resulting solids were filtered, washed with water and dried and the filtrate was concentrated. The dried solids and filtrate residue were partitioned between dichloromethane and water. The organic layer was washed with brine, dried (Na_2_SO_4_) and concentrated. The crude material was adsorbed onto silica gel and purified via ISCO using 0-30% ethyl acetate from hexanes to yield 727 mg (25%) of a yellow solid (***int-9***). ^1^H-NMR (CDCl_3_) δ 8.00-7.95 (m, 1 H), 7.75-7.68 (m, 7 H), 7.64-7.57 (m, 2 H); LC-MS, calculated for C_17_H_10_F_6_N_2_ (MH)^+^ 357.2; observed 357.0.

***int-10*:** A mixture containing ***int-9*** (727 mg, 2.04 mmol), 4-fluoronitrobenzene (864 mg, 6.12 mmol) and K_2_CO_3_ (1.0 g, 7.23 mmol) in N, N-dimethylformamide (21 mL) was heated to 90^o^ C for 21 hours. Upon cooling to room temperature, the reaction was poured into saturated aqueous LiCl solution and extracted with ethyl acetate. The combined organics were washed with water (5 x) and brine, dried (MgSO_4_), filtered and concentrated. The crude material was adsorbed onto silica gel and purified via ISCO using 1-20% ethyl acetate from hexanes to yield 465 mg (48%) of an off-white solid (***int-10***). ^1^H-NMR (CDCl_3_) δ 8.36 (d, 2 H, J = 9 Hz), 7.70 (dd, 4 H, J = 6 Hz, 9 Hz), 7.57 (d, 3 H, J = 6 Hz), 7.49 (d, 4 H, J = 9 Hz); ESI-MS, calculated for C_23_H_13_F_6_N_3_O_2_ (MH)^+^ 478.3; observed 478.0.

**76:** A mixture containing ***int-10*** (465 mg, 0.974 mmol), stannous chloride dihydrate (770 mg, 3.41 mmol) and concentrated HCl (1.5 mL, 18.0 mmol) in ethanol (10 mL) was heated to 50^o^ C for 3.5 hours. Upon cooling to room temperature, the solvent was concentrated and the residue was diluted with ethyl acetate and 2 N NaOH. The aqueous layer was extracted with ethyl acetate and the combined organics were washed with water and brine, dried (Na_2_SO_4_), filtered and concentrated to yield 537 mg (> 100%) of a yellow gel (**76**), which required no further purification. ^1^H-NMR (CDCl_3_) δ 7.69 (dd, 4 H, J = 6 Hz, 9 Hz), 7.52 (dd, 4 H, J = 6 Hz, 9 Hz), 7.44 (s, 1 H), 7.07 (dd, 2 H, J = 9 Hz), 6.71 (d, 2 H, J = 9 Hz), 3.91 (br s, 2 H).

**77:** A mixture containing **76** (0.974 mmol), Boc-glycine (260 mg, 1.46 mmol), diisopropylethylamine (0.6 mL, 3.44 mmol) and Propylphosphonic anhydride solution, 50 wt. % in ethyl acetate (1.8 mL, 3.02 mmol) in anhydrous tetrahydrofuran (35 mL) was sealed tightly and stirred at room temperature for 68 hours. The solvent was concentrated to 20% volume and the residue was partitioned between ethyl acetate and saturated aqueous NaHCO_3_. The organic layer was washed with brine, dried (Na_2_SO_4_), filtered and concentrated. The crude material was adsorbed onto silica gel and purified via ISCO using 1-50% ethyl acetate from hexanes to yield 542 mg (92%) of an off-white solid (**77**). ^1^H-NMR (CDCl_3_) δ 8.52 (br s, 1 H), 7.71-7.63 (m, 7 H), 7.53-7.47 (m, 5 H), 7.28-7.22 (m, 1 H), 5.24 (br s, 1 H), 3.94 (d, 2 H, J = 6 Hz), 1.49 (s, 9 H).

**79:** A solution of **77** (540 mg, 0.893 mmol) in dichloromethane (26 mL) was cooled to 0^o^ C and treated with trifluoroacetic acid (1.4 mL, 18.8 mmol). The reaction warmed to room temperature and stirred for 18 hours. Upon completion, the mixture was concentrated and the residue was partitioned between ethyl acetate and 2 N aqueous NaOH. The aqueous layer was extracted with ethyl acetate and the combined organic layers were washed with brine, dried (Na_2_SO_4_), filtered and concentrated to yield 405 mg (90%) of a yellow solid (**79**), which required no further purification. ^1^H-NMR (CDCl_3_) δ 9.68 (br s, 1 H), 7.72 (d, 2 H, J = 9 Hz), 7.64 (dd, 4 H, J = 9 Hz), 7.55-7.49 (m, 5 H), 7.25 (d, 2 H, J = 6 Hz), 3.52 (s, 2 H). LC-MS, calculated for C_25_H_18_F_6_N_4_O (MH)^+^ 504.4; observed 505.2.

**197:** A mixture containing **79** (405 mg, 0.805 mmol), 3-fluorobenzyl bromide (0.11 mL, 0.897 mmol) and triethylamine (0.28 mL, 2.01 mmol) in dimethylformamide (7 mL) was stirred for 25 hours at room temperature. The reaction was poured into a saturated aqueous LiCl solution and extracted with ethyl ether. The organic layer was washed with brine, dried (Na_2_SO_4_), filtered and concentrated to obtain 523 mg of a yellow gel. The crude material was purified over silica gel using 0-5% methanol from dichloromethane to yield 212 mg (43%) of an off-white solid (**197**). ^1^H-NMR (CDCl_3_) δ 9.39 (s, 1 H), 7.71-7.64 (m, 6 H), 7.54-7.47 (m, 6 H), 7.37-7.23 (m, 3 H), 7.11-6.96 (m, 3 H), 3.88 (s, 2 H), 3.47 (s, 2 H). ^13^C-NMR (CDCl_3_, 75 MHz) δ 169.7, 138.3, 133.1, 130.5, 129.4, 128.8, 127.4, 126.7, 125.8, 123.7, 120.3, 115.0, 114.8, 53.6, 52.4. LC-MS, calculated for C_32_H_23_F_7_N_4_O (MH)^+^ 613.5; observed 613.2. Anal. Calculated for C_32_H_23_F_7_N_4_O; C, 62.74; H, 3.78; N, 9.14. Found: C, 62.46; H, 3.86; N, 9.09.


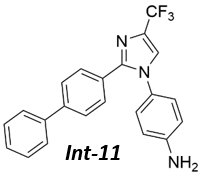


***int-11*:** Using (1,1’-biphenyl)-4-carboxaldehyde as the starting material through the procedures to prepare **76**, ***int-11*** was isolated as a yellow solid (3.81 g). ^1^H-NMR (CDCl_3_) δ 7.61 (dd, 2 H, J = 6 Hz), 7.50 (dd, 4 H, J = 3 Hz, 6 Hz), 7.45-7.32 (m, 4 H), 7.05 (d, 2 H, J = 6 Hz), 6.71 (d, 2 H, J = 6 Hz), 3.89 (br s, 2 H).


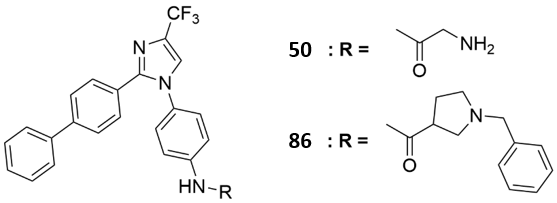


**50:** Following the procedures for preparing **77** and **79**, **50** was isolated as an off-white solid (264 mg, 79% for the final step). ^1^H-NMR (CDCl_3_) δ 9.66 (br s, 1 H), 7.74 (d, 2 H, J = 9 Hz), 7.57 (d, 2 H, J = 9 Hz), 7.50 (dd, 4 H, J = 9 Hz), 7.43 (dd, 2 H, J = 6 Hz, 9 Hz), 7.36 (d, 1 H, J = 9 Hz), 7.32-7.24 (m, 3 H), 3.52 (s, 2 H). ESI-MS, calculated for C_24_H_19_F_3_N_4_O (MH)^+^ 437.4; observed 437.0. Anal. Calculated (with 0.2 mol water) for C_24_H_19_F_3_N_4_O; C, 65.50; H, 4.44; N, 12.73. Found: C, 66.04; H, 4.38; N, 12.83.

**86:** A mixture containing ***int-11*** (400 mg, 1.05 mmol), 1-(Phenylmethyl)-3-pyrrolidinecarboxylic acid (325 mg, 1.58 mmol), diisopropylethylamine (0.65 mL, 3.73 mmol) and Propylphosphonic anhydride solution, 50 wt. % in ethyl acetate (1.9 mL, 3.19 mmol) in anhydrous tetrahydrofuran (38 mL) was sealed tightly and stirred at room temperature for 18 hours. The solvent was concentrated to 20% volume and the residue was partitioned between ethyl acetate and saturated aqueous NaHCO_3_. The organic layer was washed with brine, dried (Na_2_SO_4_), filtered and concentrated. The crude material was adsorbed onto silica gel and purified via ISCO using 0-10% methanol from dichloromethane to yield 513 mg (86%) of an off-white solid (**86**). ^1^H-NMR (CDCl_3_) δ 9.77 (s, 1 H), 7.57 (dd, 4 H, J = 6 Hz, 9 Hz), 7.48 (dd, 6 H, J = 6 Hz), 7.42 (d, 1 H, J = 6 Hz), 7.37-7.32 (m, 5 H), 7.29-7.20 (m, 4 H), 3.73 (dd, 2 H, J = 6 Hz, 12 Hz), 3.16 (dd, 2 H, J = 6 Hz, 9 Hz), 2.95 (dd, 1 H, J = 6 Hz, 9 Hz), 2.42-2.33 (m, 3 H), 2.12-2.05 (m, 1 H). LC-MS, calculated for C_34_H_29_F_3_N_4_O (MH)^+^ 567.6; observed 567.2. Anal. Calculated for C_34_H_29_F_3_N_4_O; C, 72.07; H, 5.15; N, 9.88. Found: C, 71.97; H, 5.25; N, 9.82.


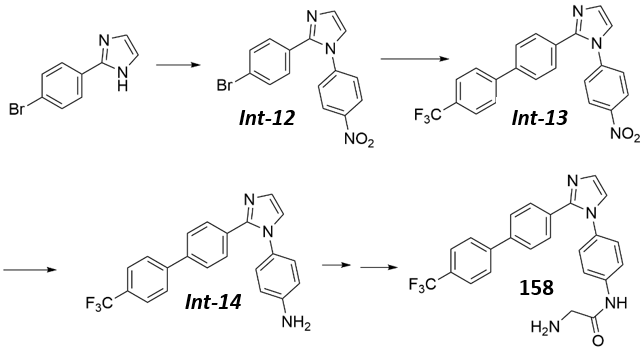


***int-12*:** A mixture containing 2-(4-Bromophenyl)-1H-imidazole (500 mg, 2.25 mmol), 4-fluoronitrobenzene (950 mg, 6.74 mmol) and K_2_CO_3_ (930 mg, 6.74 mmol) in N, N-dimethylformamide (21 mL) was heated to 90^o^ C for 21 hours. Upon cooling to room temperature, the poured into saturated aqueous LiCl solution and extracted with ethyl acetate. The combined organics were washed with water (5 x) and brine, dried (MgSO_4_), filtered and concentrated. The crude material was adsorbed onto silica gel and purified via ISCO using 0-5% methanol from dichloromethane to yield 700 mg (90%) of an off-white solid (***int-12***).

***int-13*:** A mixture containing ***int-12*** (500 mg, 1.46 mmol), 4-trifluoromethylphenylboronic acid (330 mg, 1.75 mmol), palladium tetrakis(triphenylphosphine) (170 mg, 0.146 mmol) and 1.0 M aqueous Na_2_CO_3_ (4.37 mL, 4.37 mmol) in ethylene glycol dimethyl ether (15 mL) was heated to 100^o^ C for 21 hours. Upon cooling to room temperature, the reaction was diluted with water and poured extracted with ethyl acetate. The combined organics were washed with brine, dried (MgSO_4_), filtered and concentrated. The crude material was adsorbed onto silica gel and purified via ISCO using 10-75% ethyl acetate from hexanes to yield 500 mg (84%) of an off-white solid (***int-13***).

***int-14*:** A mixture containing ***int-13*** (500 mg, 1.22 mmol), stannous chloride dihydrate (1.85 g, 9.78 mmol) and 1.0 M HCl (24 mL, 24 mmol) in ethanol (40 mL) was heated to 50^o^ C for 3.5 hours. Upon cooling to room temperature, the solvent was concentrated and the residue was diluted with ethyl acetate and 2 N NaOH. The aqueous layer was extracted with ethyl acetate and the combined organics were washed with water and brine, dried (Na_2_SO_4_), filtered and concentrated. The crude material was adsorbed onto silica gel and purified via ISCO using 0-5% methanol from dichloromethane to yield 470 mg (>100%) of an off-white solid (***int-14***).

**158:** A mixture containing ***int-14*** (50 mg, 0.132 mmol), Boc-glycine (69 mg, 0.394 mmol), diisopropylethylamine (0.15 mL, 0.792 mmol) and Propylphosphonic anhydride solution, 50 wt. % in ethyl acetate (0.25 mL, 0.394 mmol) in anhydrous tetrahydrofuran (20 mL) was sealed tightly and stirred at room temperature for 18 hours. The solvent was diluted with ethyl acetate (20 mL) and saturated aqueous NaHCO_3_ (25 mL). The organic layer was washed with brine, dried (Na_2_SO_4_), filtered and concentrated. The crude material was adsorbed onto silica gel and purified via ISCO using 10-50% ethyl acetate from hexanes to yield 50 mg (0.0933 mmol, 71%) of an off-white solid, which was dissolved in methanol (5 mL), cooled to 0^o^ C and treated with 4.0 N hydrochloric acid in dioxane (1.2 mL, 4.8 mmol). The reaction warmed to room temperature and stirred for 18 hours. Upon completion, the mixture was concentrated and the residue was partitioned between dichloromethane and saturated aqueous NaHCO_3_. The aqueous layer was extracted with dichloromethane and the combined organic layers were washed with brine, dried (Na_2_SO_4_), filtered and concentrated. The crude material was adsorbed onto silica gel and purified via ISCO using 0-10% methanol from dichloromethane to yield 25 mg (63%) of an off-white solid (**158**). ^1^H-NMR (CDCl_3_) δ 9.60 (br s, 1 H), 7.68 (dd, 4 H, J = 8.4 Hz, 9.6 Hz), 7.49 (dd, 4 H, J = 2.0 Hz), 7.29-7.21 (m, 6 H), 3.50 (s, 2 H). LC-MS, calculated for C_24_H_19_F_3_N_4_O(MH)^+^ 436.4; observed 437.2.


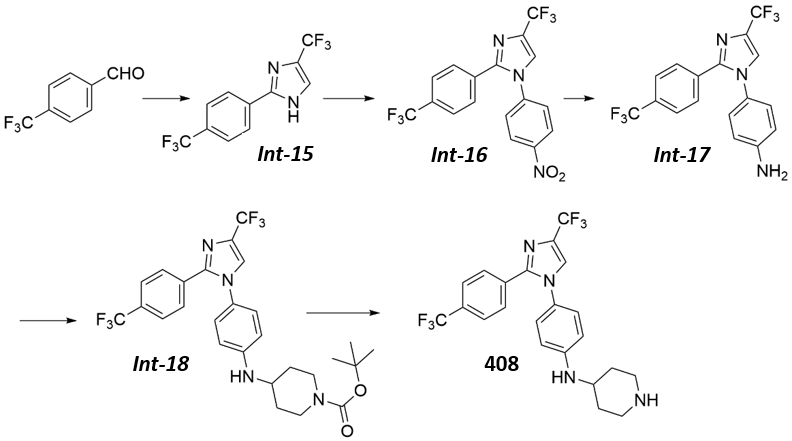


***int-15*:** A mixture containing 1,1-Dibromo-3,3,3-trifluoroacetone (4.64 g, 17.2 mmol) and sodium acetate (1.88 g, 23.0 mmol) in water (42 mL) was heated to 95^o^ C for 1 hour, then cooled to room temperature. 4’-Trifluoromethylbenzaldehyde (2.0 g, 11.4 mmol) in methanol (102 mL) was cooled to 0^o^ C and treated with dropwise addition of ammonium hydroxide (50 mL). This mixture stirred for 10 minutes at 0^o^ C and then the first mixture was added dropwise; the combined reaction slowly warmed to room temperature and stirred for 18 hours. The resulting solids were filtered, washed with water and dried and the filtrate was concentrated. The dried solids and filtrate residue were partitioned between dichloromethane and water. The organic layer was washed with brine, dried (Na_2_SO_4_) and concentrated. The crude material was adsorbed onto silica gel and purified via ISCO using 0-30% ethyl acetate from hexanes to yield 1.0 g (33%) of a yellow solid (***int-15***).

***int-16*:** A mixture containing ***int-15*** (1.0 g, 3.57 mmol), 4-fluoronitrobenzene (1.52 g, 10.7 mmol) and K_2_CO_3_ (1.48 g, 10.7 mmol) in N, N-dimethylformamide (40 mL) was heated to 90^o^ C for 21 hours. Upon cooling to room temperature, the poured into saturated aqueous LiCl solution and extracted with ethyl acetate. The combined organics were washed with water (5 x) and brine, dried (MgSO_4_), filtered and concentrated. The crude material was adsorbed onto silica gel and purified via ISCO using 0-5% methanol from dichloromethane to yield 1.0 g (70%) of an off-white solid (***int-16***).

***int-17*:** A mixture containing ***int-16*** (1.0 g, 2.49 mmol), stannous chloride dihydrate (3.77 g, 19.9 mmol) and concentrated HCl (4.15 mL, 49.9 mmol) in ethanol (40 mL) was heated to 50^o^ C for 3.5 hours. Upon cooling to room temperature, the solvent was concentrated and the residue was diluted with ethyl acetate and 2 N NaOH. The aqueous layer was extracted with ethyl acetate and the combined organics were washed with water and brine, dried (Na_2_SO_4_), filtered and concentrated. The crude material was adsorbed onto silica gel and purified via ISCO using 0-5% methanol from dichloromethane to yield 275 mg (70%) of an off-white solid (***int-17***).

**408:** A mixture containing ***int-17*** (100 mg, 0.269 mmol), tert-Butyl 4-oxopiperidine-1-carboxylate (160 mg, 0.809 mmol) and anhydrous sodium sulfate (catalytic amount) in acetic acid (5 mL) was stirred at room temperature for 2 hours. Sodium triacetoxyborohydride (360 mg, 1.61 mmol) was added and the reaction stirred at room temperature for 18 hours. The reaction was concentrated and the residue was partitioned between ethyl acetate and saturated aqueous NaHCO_3_. The crude material was adsorbed onto silica gel and purified via ISCO using 10-75% ethyl acetate from hexanes to yield 100 mg (0.181 mmol, 67%) of an off-white solid, which was dissolved in anhydrous dioxane (5 mL), cooled to 0^o^ C and treated with 4.0 N hydrochloric acid in dioxane (2.25 mL, 9.03 mmol). The reaction warmed to room temperature and stirred for 18 hours. Upon completion, the mixture was concentrated and the residue was partitioned between dichloromethane and saturated aqueous NaHCO_3_. The aqueous layer was extracted with dichloromethane and the combined organic layers were washed with brine, dried (Na_2_SO_4_), filtered and concentrated to obtain 25 mg (30%) of an off-white solid (**408**). ^1^H-NMR (CDCl_3_) δ 7.56 (d, 2 H, J = 8.0 Hz), 7.51 (d, 2 H, J = 8.4 Hz), 7.41 (s, 1 H), 7.00 (dd, 2 H, J = 6.8 Hz, 8.8 Hz), 6.62 (dd, 2 H, J = 8.8 Hz, 9.6 Hz), 3.78-3.61 (m, 4 H), 3.27-3.17 (m, 2 H), 3.12-3.01 (m, 1 H), 2.36-2.23 (m, 2 H). LC-MS, calculated for C_22_H_20_F_6_N_4_(MH)^+^ 455.4; observed 455.2.

Triazole Scaffold:


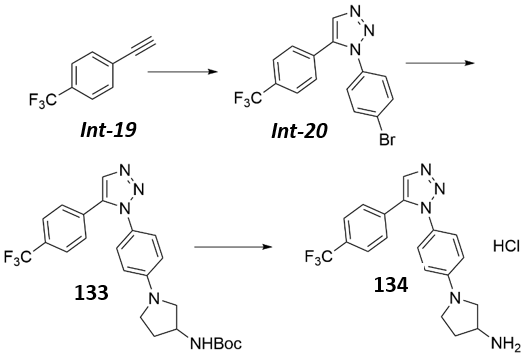


***int-20*:** A mixture containing ***int-19*** (198 mg, 1.0 mmol) and 4-Trifluoromethylstyrene (179 mg, 1.05 mmol) in dimethylsulfoxide (3.3 mL) was treated with tetramethylammonium hydroxide (25% solution - 9.1 mg/37uL of water) and stirred at room temperature for 20 hours. The reaction was diluted with water (15 mL) and extracted with ethyl acetate. The organic layer was washed with water and brine, dried (Na_2_SO_4_), filtered and concentrated. The crude material was adsorbed onto silica gel and purified via ISCO using 5-30% ethyl acetate from hexanes to yield 350 mg (95%) of a yellow-brown solid (***int-20***).

**133:** The following were combined in a heavy-duty glass reactor: ***int-20*** (110 mg, 0.3 mmol), 3-N-Boc-aminopyrrolidine (72.4 mg, 0.39 mmol), BINAP (56 mg, 0.09 mmol), Pd_2_(dba)_3_ (27.5 mg, 0.03 mmol) and Cs_2_CO_3_ (127 mg, 0.39 mmol) in anhydrous toluene (3 mL) and nitrogen gas was bubbled into the mixture for two minutes. The reactor was then sealed with a Teflon cap and heated to 110^o^ C for 15 hours. Upon cooling, the mixture was filtered through Celite and the filter pad was rinsed with ethyl acetate. The filtrate was washed with water and brine, dried (Na_2_SO_4_), filtered and concentrated. The crude material was purified over silica gel using 0-100% ethyl acetate from hexanes to yield 158.5 mg (>100%) of a yellow solid (**133**). ^1^H-NMR (CDCl_3_) δ 7.89 (s, 1 H), 7.59 (d, 2 H, J = 7.6 Hz), 7.37 (d, 2 H, J = 8.4 Hz), 7.15 (d, 2 H, J = 8.8 Hz), 6.53 (d, 2 H, J = 8.8 Hz), 4.74-4.68 (m, 1 H), 4.44-4.36 (m, 1 H), 3.66-3.60 (m, 1 H), 3.47-3.42 (m, 1 H), 3.41-3.34 (m, 1 H), 3.21-3.16 (m, 1 H), 2.36-2.17 (m, 1 H), 2.04-1.97 (m, 1 H), 1.44 (s, 9 H). LC-MS, calculated for C_24_H_26_F_3_N_5_O_2_ (MH)^+^ 473.5; observed 474.2.

**134:** A solution of **133** (158.5 mg, 0.334 mmol) in dichloromethane (20 mL) was cooled to 0^o^ C and treated with trifluoroacetic acid (0.5 mL, 6.73 mmol). The reaction warmed to room temperature and stirred for 15 hours. Upon completion, the mixture was concentrated and the residue was partitioned between ethyl acetate and 2 N aqueous NaOH. The aqueous layer was extracted with ethyl acetate and the combined organic layers were washed with brine, dried (Na_2_SO_4_), filtered and concentrated to yield 112.5 mg (90%) of a yellow solid, which required no further purification. This material was dissolved in diethyl ether and treated with 2 N HCl/diethyl ether, stirred at room temperature for 18 hours, filtered, washed with diethyl ether and dried to yield 104 mg (76%) of **134** as a white solid. ^1^H-NMR (CD_3_OD) δ 8.08 (s, 1 H), 7.63 (d, 2 H, J = 8.0 Hz), 7.48 (d, 2 H, J = 8.4 Hz), 7.21 (d, 2 H, J = 8.8 Hz), 6.71 (d, 2 H, J = 8.8 Hz), 4.09-4.01 (m, 1 H), 3.72-3.61 (m, 2 H), 3.49-3.42 (m, 2 H), 2.54-2.44 (m, 2 H). ^13^C-NMR (CD_3_OD, 75 MHz) δ 148.2, 137.1, 132.8, 129.2, 129.1, 128.1, 126.6, 125.5, 125.4, 125.3, 125.2, 124.7, 118.1, 112.2, 50.3, 48.3, 47.0, 29.1. LC-MS, calculated for C_19_H_18_F_3_N_5_ (MH)^+^ 374.4; observed 374.2. Anal. Calculated (with 0.4 mol diethyl ether) for C_19_H_19_ClF_3_N_5_; C, 54.62; H, 5.45; N, 15.46. Found: C, 54.64; H, 5.05; N, 15.05.


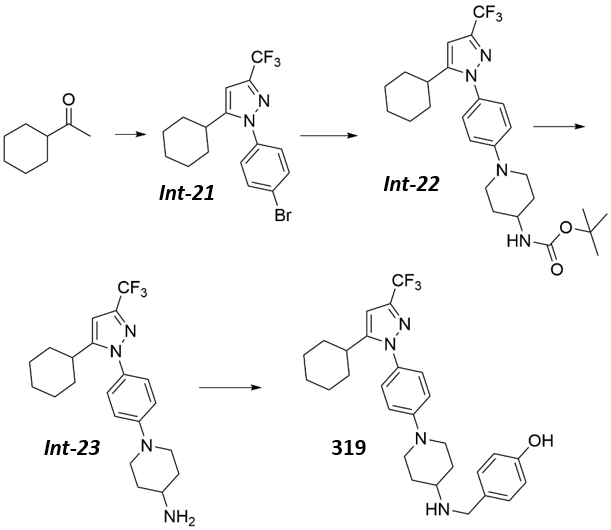


***int-21*:** Into an oven dried flask was introduced sodium hydride (60% wt./mineral oil, 9.52 g, 237.6 mmol) and stirred in anhydrous THF (140 mL) for 5 minutes at room temperature. Ethyl trifluoroacetate (18.9 mL, 158.8 mmol) was added dropwise and this mixture stirred at room temperature for 10 minutes. A solution of cyclohexyl methyl ketone (10 g, 79.2 mmol) in anhydrous THF (40 mL) was added dropwise and the reaction mixture was refluxed for 3 hours. The reaction was concentrated and the residue was partitioned between ethyl acetate and water. The aqueous layer was extracted with ethyl acetate and the combined organics were washed with brine, dried (Na_2_SO_4_) and concentrated to yield a quantitative yield of a yellow oil, which was used without further purification. ^1^H-NMR (CD_3_OD) δ 5.24 (s, 1 H), 2.94-2.84 (m, 1 H), 1.82-1.66 (m, 4 H), 1.40-1.19 (m, 6 H).

A mixture containing the previous product (79.2 mmol) and 4-bromohydrazine hydrochloride (24 g, 103 mmol) in ethanol (1 L) was refluxed for 18 hours. The solvent was concentrated and the residue was partitioned between ethyl acetate and saturated aqueous NaHCO_3_. The aqueous layer was extracted with ethyl acetate and the combined organics were washed with brine, dried (MgSO_4_), filtered and concentrated. The crude material was purified over silica gel via a flush column using 3-5% ethyl acetate from hexanes to remove baseline impurities from the isomeric product mixture. Upon sitting at room temperature, a yellow solid (undesired isomer) precipitated from the mixture. The liquid portion of the mixture (rich in ***int-21***) was decanted and the yellow solid was triturated in hexanes to remove all of ***int-21*** (with some of the undesired isomer) and filtered. This combined filtrate and decantate was concentrated, adsorbed onto silica gel and purified via ISCO using 2-15% dichloromethane from hexanes to yield 4.58 g (15%) of an orange oil (***int-21***). ^1^H-NMR (CDCl_3_) δ 7.59 (d, 2 H, J = 9 Hz), 7.37 (d, 2 H, J = 9 Hz), 6.62 (s, 1 H), 2.75-2.66 (m, 1 H), 2.09-1.99 (m, 2 H), 1.90-1.70 (m, 2 H), 1.53-1.21 (m, 6 H). Anal. Calculated for C_16_H_16_BrF_3_N_2_; C, 51.48; H, 4.32; N, 7.50. Found: C, 51.73; H, 4.51; N, 7.51.

***int-22*:** The following were combined in a heavy-duty glass reactor: ***int-21*** (1.1 g, 2.95 mmol), 4-(tert-butoxycarbonylamino)piperidine (1.18 g, 5.89 mmol), BINAP (551 mg, 0.884 mmol), Pd_2_(dba)_3_ (540 mg, 0.589 mmol) and Cs_2_CO_3_ (1.92 g, 5.89 mmol) in anhydrous toluene (40 mL) and nitrogen gas was bubbled into the mixture for two minutes. The reactor was then sealed with a Teflon cap and heated to 110^o^ C for 15 hours. Upon cooling, the mixture was filtered through Celite and the filter pad was rinsed with ethyl acetate. The filtrate was washed with water and brine, dried (Na_2_SO_4_), filtered and concentrated to obtain 3.51 g of a rust-colored gel. The crude material was purified over silica gel using 0-100% ethyl acetate from hexanes to yield 426 mg (29%) of a yellow solid (***int-22***). ^1^H-NMR (CDCl_3_) δ 7.31 (d, 2 H, J = 9 Hz), 6.93 (d, 2 H, J = 9 Hz), 6.55 (s, 1 H), 4.48 (br s, 1 H), 3.73-3.64 (m, 3 H), 2.91 (dd, 2 H, J = 3 Hz, 9 Hz), 2.74-2.67 (m, 1 H), 2.07-2.00 (m, 4 H), 1.84-1.67 (m, 7 H), 1.62-1.25 (m, 7 H), 1.46 (s, 9 H).

***int-23*:** A solution of ***int-22*** (426 mg, 0.865 mmol) in dichloromethane (17 mL) was cooled to 0^o^ C and treated with trifluoroacetic acid (0.65 mL, 8.75 mmol). The reaction warmed to room temperature and stirred for 15 hours. Upon completion, the mixture was concentrated and the residue was partitioned between ethyl acetate and 2 N aqueous NaOH. The aqueous layer was extracted with ethyl acetate and the combined organic layers were washed with brine, dried (Na_2_SO_4_), filtered and concentrated to yield 281 mg of ***int-23*** (>100%) of a yellow gel, which required no further purification. ^1^H-NMR (CDCl_3_) δ 7.30 (d, 2 H, J = 9 Hz), 6.94 (d, 2 H, J = 9 Hz), 6.55 (s, 1 H), 3.75-3.69 (m, 2 H), 2.89-2.80 (m, 2 H), 2.73-2.66 (m, 1 H), 2.10-1.71 (m, 7 H), 1.53-1.27 (m, 8 H). LC-MS, calculated for C_21_H_27_F_3_N_4_ (MH)^+^ 393.4; observed 393.0.

**319:** A solution of ***int-23*** (262 mg, 0.667 mmol), 4-hydroxybenzaldehyde (83 mg, 0.68 mmol) and 4A molecular sieves (300 mg) in anhydrous methanol (7.5 mL) and anhydrous tetrahydrofuran (3.5 mL), was stirred at room temperature for 18 hours. The reaction was cooled to 0^o^ C and treated with sodium borohydride (51 mg, 1.33 mmol); the reaction stirred for 4 hours at room temperature. The reaction was concentrated and the residue partitioned between saturated aqueous sodium bicarbonate solution and ethyl acetate. The combined organic layers were washed with brine, dried (Na_2_SO_4_), filtered and concentrated to yield 390 mg of a yellow gel. The crude material was purified over silica gel using 0.5-5 % methanol from dichloromethane to yield 146 mg (44%) of **319** as a white solid. ^1^H-NMR (CDCl_3_) δ 7.30 (d, 2 H, J = 9 Hz), 7.17 (d, 2 H, J = 9 Hz), 6.93 (d, 2 H, J = 9 Hz), 6.72 (d, 2 H, J = 9 Hz), 6.55 (s, 1 H), 3.77 (s, 2 H), 3.72 (dd, 2 H, J = 12 Hz), 2.80 (dd, 2 H, J = 12 Hz), 2.74-2.65 (m, 2 H), 2.36 (br s, 1 H), 2.02 (d, 4 H, J = 12 Hz), 1.89-1.71 (m, 3 H), 1.62-1.22 (m, 7 H). ^13^C NMR (CDCl_3_, 75 MHz) δ 151.5, 129.4, 126.5, 115.6, 115.4, 54.0, 50.2, 47.9, 37.3, 33.0, 32.1, 26.2, 26.0; ESI-MS, calculated for C_28_H_33_F_3_N_4_O (M)^-^ 497.6; observed 497.2. Anal. Calculated for C_28_H_33_F_3_N_4_O; C, 67.45; H, 6.67; N, 11.23. Found: C, 67.19; H, 6.63; N, 11.11.


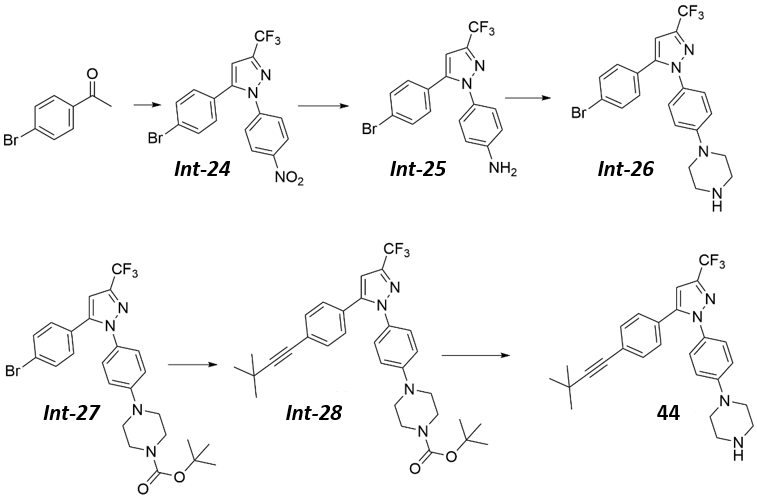


***int-24*:** To a suspension of sodium hydride (60% wt./mineral oil, 1.69 g, 70.3 mmol) and stirred in anhydrous THF (50 mL) in a 3-neck flask fitted with a reflux condenser, ethyl trifluoroacetate (7.14 g, 50.2 mmol), in 6 mL of anhydrous THF, was added dropwise; this mixture stirred at room temperature for 10 minutes. A solution of p-Bromoacetophenone (5 g, 25.1 mmol) in anhydrous THF (25 mL) was added dropwise, over 30 minutes, and the reaction mixture was refluxed for 3 hours. The reaction was quenched acetic acid/water (5 mL, 1/1 ratio) and concentrated. The residue was partitioned between ethyl acetate and acetic acid/water and the combined organics were washed with brine, dried (Na_2_SO_4_) and concentrated to generate 6.5 g (88%) of a yellow solid, which was used without further purification.

A mixture containing the previous product (0.733 g, 2.5 mmol) and 4-nitrohydrazine hydrochloride (0.57 g, 3.0 mmol) in ethanol (25 mL) was refluxed for 2 hours. Another 47 mg (0.25 mmol) of 4-nitrohydrazine hydrochloride was added and the reaction refluxed for 15 hours. The solvent was concentrated and the residue was partitioned between ethyl acetate and water. The aqueous layer was extracted with ethyl acetate and the combined organics were washed with brine, dried (MgSO_4_), filtered and concentrated. The crude material was adsorbed onto silica gel and purified via ISCO using 0.5-10% ethyl acetate from hexanes to yield 0.69 g (67%) of a white solid (***int-24***).

***int-25*:** A mixture containing ***int-24*** (82.4 mg, 0.20 mmol), stannous chloride dihydrate (133 mg, 0.70 mmol) and concentrated HCl (0.3 mL, 3.6 mmol) in ethanol (1.5 mL) was heated to 50^o^ C for 2.5 hours. Upon cooling to room temperature, the solvent was concentrated and the residue was diluted with ethyl acetate and 2 N NaOH. The aqueous layer was extracted with ethyl acetate and the combined organics were washed with water and brine, dried (Na_2_SO_4_), filtered and concentrated to yield 75 mg (98%) of an off-white solid (***int-25***), which required no further purification.

***int-26*:** A mixture containing ***int-25*** (38.2 mg, 0.10 mmol), Bis(2-chloroethyl)amine hydrochloride (35.6 mg, 0.20 mmol) and K_2_CO_3_ (27.6 mg, 0.20 mmol) in 1-methoxyethanol (1.0 mL) was heated to 175^o^ C for 2 hours via microwaves. The reaction was heated for another 1 hour to enhance completion. Upon cooling to room temperature, the reaction was diluted with 2 N NaOH. The mixture was extracted with ethyl acetate and the combined organics were washed with water and brine, dried (Na_2_SO_4_), filtered and concentrated. The crude material was adsorbed onto silica gel and purified via ISCO using 0-15% (1% NH_4_OH/methanol) from dichloromethane to yield 27 mg (60%) of a colorless oil (***int-26***).

***int-27*:** A mixture containing ***int-26*** (44 mg, 0.097 mmol), di-tert-butyl dicarbonate (23.4 mg, 0.107 mmol) in ethanol (1.0 mL) was heated to 30^o^ C for 30 minutes. The reaction was concentrated and diluted with water. The mixture was extracted with ethyl acetate and the combined organics were washed with water and brine, dried (Na_2_SO_4_), filtered and concentrated. The crude material was adsorbed onto silica gel and purified via ISCO using 0-20% ethyl acetate from hexanes to yield 29 mg (54%) of a colorless oil (***int-27***).

***int-28*:** The following were combined in an I-CHEM vial with a screw cap septum: ***int-27*** (43.8 mg, 0.08 mmol), 3,3-Dimethyl-1-butyne (16 mg, 0.16 mmol), Palladium tetrakis(triphenylphosphine) (9.2 mg, 0.008 mmol), CuI (3.0 mg, 0.016 mmol) in diisopropylamine (2 mL). The vial was repeatedly evacuated and backfilled with nitrogen. The reaction was heated to 70^o^ C for 15 hours. Upon cooling, the mixture was filtered through Celite and the filter pad was rinsed with ethyl acetate. The filtrate was concentrated and the crude material was purified over silica gel using 0-18% ethyl acetate from hexanes to yield 43 mg (94%) of a colorless oil (***int-28***).

**44:** A solution of ***int-28*** (34 mg, 0.061 mmol) in dichloromethane (2 mL) was cooled to 0^o^ C and treated with trifluoroacetic acid (0.2 mL, 2.69 mmol). The reaction warmed to room temperature and stirred for 15 hours. Upon completion, the mixture was concentrated and the residue was partitioned between ethyl acetate and 2 N aqueous NaOH. The aqueous layer was extracted with ethyl acetate and the combined organic layers were washed with brine, dried (Na_2_SO_4_), filtered and concentrated to yield 281 mg of **44** (>100%) of a yellow gel, which required no further purification. ^1^H-NMR (CD_3_OD) δ 7.67-7.61 (m, 2 H), 7.58-7.52 (m, 2 H), 7.30-7.17 (m, 4 H), 3.49-3.43 (m, 4 H), 3.37-3.32 (m, 4 H), 1.27 (s, 9 H). LC-MS, calculated for C_26_H_27_F_3_N_4_ (MH)^+^ 452.5; observed 453.2.
