# Supplementary material for: Synthesis and preliminary evaluation of novel compounds that demonstrate broad host-directed anti-leishmanial activity
Source: PLoS Negl Trop Dis. 2026 Jul 13;20(7):e0014520. doi: 10.1371/journal.pntd.0014520 (PMC13379085; doi:10.1371/journal.pntd.0014520)
Supplement: Attachment — Submitted filename: Response to Reviewers.docx [file pntd.0014520.s016.docx]

**Response to Reviewers**

We would like to thank the reviewers for their time spent with this manuscript. A response to reviewers has been prepared and edits to the manuscript have been made accordingly. We believe the manuscript has been significantly strengthened because of the comments of the reviewers and thank them again for providing the comments as well as responding to the revision. Below the reviewers’ response, we have indicated our response in blue text.

**Reviewer #1:** The authors evaluated a library of AR-12-based compounds against Leishmania spp. The study is innovative and shows the potential of the candidate molecules. Above all, in the assays for target search and confirmation and mechanism of action, the results proved promising. For this peer review, this manuscript is considered promising for publication.

We thank the reviewer for their positive assessment of our work and for recognizing the novelty of our approach, as well as the potential translational significance of these compounds for the development of host-directed therapies against leishmaniasis.

**Reviewer #2:** Overall, this manuscript addresses an interesting question on the development of host-targeting treatments. Experiments are well designed and appropriately interpreted. However, I have several concerns that must be addressed. Major concerns: 

1. Fig 3, RTI-354 L. donovani treatment, why is there a 0.01 um data point when all the other DRC curves end at 0.1?

We thank the reviewer for identifying this inconsistency. This was an error in the figure presentation, and the graph has been corrected in the revised manuscript.

 2. Why don't all the DRC plots in Fig 3 have the same number of data points? This also applies to Fig S3-S7. If data was suppressed, a clear rationale should be provided and non-suppressed data included in the SI.

We appreciate this observation. In the original figures, we included only the concentration range spanning the active portion of the dose-response curve (i.e., around the IC₅₀ or LC₅₀ values). For example, not including lower concentrations where the viability was 100% or higher concentration where the viability was 0%. However, we can see how this could be confusing. For transparency, we have now included the full data set in the figures.

3. For protein enrichment and thermal proteome profiling analyses: is the p-value corrected for multiple hypothesis testing (eg FDR correction or similar)? If yes, correction method should be specified in the methods, and text and figure legends should clarify that the p-values are corrected. Using uncorrected p-values is not appropriate.

We thank the reviewer for the opportunity to clarify our analytical approach. The primary analyses were not based solely on p-values but instead incorporated both statistical significance and fold-change thresholds. Furthermore, candidate targets were prioritized only after cross-validation using an independent proteomic approach. To address the reviewer’s concern, we have now included Bonferroni-corrected p-values (q values) in the raw data file.

4. Page 16, interpretation of Figure S8: the modified and unmodified compounds do not actually show the same curves. The lack of significant differences could be because of the big error bars. Additional replicates with less technical variation should be performed to confirm whether they perform the same or not.

We appreciate the reviewer’s concern. However, we do not believe that additional replication of this assay is necessary for the conclusions of the current study. The thermal proteome profiling experiments were conducted using the non-ligated parent compound (**197**) to further support this point. The comparison shown in **Figure S8** (now **Figure S9**) was included primarily to demonstrate that chemical modification did not result in a substantial loss of activity. While some variability is present, the overall activity profiles remain comparable and do not affect the interpretation of the proteomic studies. 
 
Minor concerns: 
5. "Further the chemical ligation to 197" (top of page 16): what does this mean? I think there are some words missing here. "Although the highest selectivity was noted with 53 in two strains, 197 displayed broad activity against the promastigote as well as host-directed activity, further the chemical ligation to 197 was more approachable than for 53."

We thank the reviewer for identifying this lack of clarity. The sentence has been revised as follows:
*“Although the highest selectivity was noted with* ***53*** *in two strains,* ***197*** *displayed broad activity against the promastigote as well as host-directed activity. Furthermore, the synthetic strategy required to introduce a chemical handle onto* ***197*** *for affinity-capture studies was more approachable than for* ***53****. Therefore,* ***197*** *was selected for subsequent proteomic analyses."*

6. Need LC-MS parameters for affinity capture.

We thank the reviewer for identifying this omission. Detailed sample preparation, LC-MS/MS acquisition parameters, and data analysis procedures have now been added to the Methods section to ensure reproducibility and transparency.

*“**Lanes (1cm) for each sample were excised and cut into 1mm cubes and placed in 1ml of destain solution for 2 hours at room temperature (RT). After removal of destain solution, each sample was washed 2x with 100% acetonitrile and incubated for 10 min at RT. Proteins were reduced with 10 mM DTT for 10 min at 55 °C, then for 20 min at RT. After dithiothreitol (DTT) was removed, samples were alkylated with 100 mM IAA for 45 min in the dark at RT, and in-gel digested with 20 ng/ul trypsin (Promega) overnight at 37˚C. Peptides were extracted with 200 ul of acetonitrile (ACN), gently vortex and incubated at RT for 10 minutes, then transferred to a new tube. This was performed twice. Peptides were desalted with C18 spin columns (Pierce) and then dried via vacuum centrifugation. Peptide samples were stored at -80˚C until further analysis.*

*The peptide samples were resuspended in 15 μL of 5% ACN/0.1% formic acid and analyzed by LC-MS/MS using an Easy nLC 1200 coupled to a QExactive HF mass spectrometer (Thermo Scientific). Samples were injected onto an Easy Spray PepMap C18 column (75 μm id × 25 cm, 2 μm particle size) (Thermo Scientific) and separated over a 90 minute method. The gradient for separation consisted of 5–45% mobile phase B at a 250 nl/min flow rate, where mobile phase A was 0.1% formic acid in water and mobile phase B consisted of 0.1% formic acid in ACN. The QExactive HF was operated in data-dependent mode where the 15 most intense precursors were selected for subsequent fragmentation. Resolution for the precursor scan (m/z 375–1700) was set to 60,000, while MS/MS scan resolution was set to 15,000. The normalized collision energy was set to 27% for higher-energy collisional dissociation (HCD). Peptide match was set to preferred, and precursors with unknown charge or a charge state of 1 and ≥ 7 were excluded.*

*Raw data files were searched against the Uniprot reviewed human database (containing 20,360 entries, downloaded January 2022), a Uniprot Leishmania database (containing 8,269 entries, downloaded April 2022), and the MaxQuant common contaminants database (246 entries), using the Sequest HT search engine node within Proteome Discoverer (v2.5, Thermo Fisher). Enzyme specificity was set to trypsin/P, up to two missed cleavage sites were allowed, methionine oxidation and N-terminus acetylation were set as variable modifications, and cysteine carbamidomethylation was set as a static modification. The Minora node was used to extract label-free quantification (LFQ) intensities. A 1% peptide-level false discovery rate (FDR) and a 5% protein-level FDR were used to filter all data. Match between runs was enabled. Data filtering, imputation, and statistical analysis was performed in Perseus software (version 1.6.14.0).”*

7. In TPP methods, AGC target should be 4x10^5 (power or superscript) not 4x105 (bottom of page 33, two instances).

We thank the reviewer for catching this typographical error. The AGC target value has been corrected.

**Reviewer #3:** * This study tackles a significant unmet need in leishmaniasis therapy and proposes a well‑defined translational pathway toward novel host‑directed treatments. Nevertheless, the additional experiments listed above are essential before the manuscript can be recommended for publication.

1. Although the proteomic data suggest that compound 197 interacts with lysozyme, no direct evidence of protein‑ligand binding has been presented. To substantiate this mechanism, the authors should perform at least one biophysical assay (e.g., DSF, ITC, SPR, or FRET) using recombinant lysozyme together with the four most promising hits and the parent compound AR‑12.

We appreciate the reviewer’s suggestion; however, we believe that the combination of affinity-capture proteomics and thermal proteome profiling provides strong orthogonal evidence supporting an interaction between compound 197 and lysozyme. Thermal proteome profiling specifically measures ligand-induced changes in protein thermal stability and is widely used to identify protein-drug interactions in biologically relevant contexts. Furthermore, our target identification strategy included biological validation studies. Evaluation of additional compounds and more detailed biophysical characterization of compound-lysozyme interactions represent important future directions.

2. The five aforementioned compounds should also be evaluated for their ability to modulate lysozyme enzymatic activity (e.g., using a muramidase assay).

We agree with the reviewer that functional validation is important. Accordingly, we performed a lysozyme activity assay using a commercially available kit (Abcam) and found that compound **197** produced a dose-dependent increase in lysozyme activity. These results have been added as **Figure 4D** and further support lysozyme as a biologically relevant target. We feel that characterization of the other leads will be important future direction.

3. Moreover, the best four hits should be evaluated against recombinant PDK-1 (e.g., using a kinase assay), the primary target of the parent compound AR-12 (IC₅₀ = 5 µM; <https://doi.org/10.1158/0008-5472.CAN-03-4063>).

We respectfully believe that this experiment falls outside the scope of the present study. The host-directed activity observed for the lead compounds occurs at concentrations below the reported IC₅₀ of AR-12 for PDK-1 inhibition (5 µM). In addition, PDK-1 was not identified as a target in either the affinity-capture or thermal proteome profiling datasets.

4. The chemical synthesis of the AR‑12 analog library is central to the study, yet the manuscript provides only a brief overview. A detailed description of the design strategy and synthetic routes should be incorporated into the Results and Discussion section.

We thank the reviewer for this suggestion. To provide greater context regarding the medicinal chemistry strategy, we have expanded the manuscript to include a detailed description of the library design rationale, scaffold diversification strategy, and synthetic approach. This additional discussion highlights the structure-activity relationship (SAR) considerations that guided library development and compound optimization.

“*To systematically expand the chemical space surrounding AR-12, we designed a modular library centered on a 5-trifluoromethyl-substituted pyrazole core. The CF3 group was strategically retained to preserve the lipophilicity and metabolic stability associated with the parent scaffold, while providing an electronically stabilized heterocyclic platform for regioselective diversification. Our design strategy utilized a two-vector approach to enable a comprehensive structure-activity relationship (SAR) exploration. The first is a C3-diversification (R1). This was undertaken to probe hydrophobic and steric interactions hypothesized to influence target engagement. We then introduced biphenyl substituents to increase aromatic surface area and rigidity, alongside a series of alkyl and aryl groups (e.g., cyclohexyl) to systematically vary steric bulk and conformational flexibility. The second site of diversity is the N2-Functionalization (R2). This was achieved through the installation of a 4-aminobenzyl handle which was selected as a synthetically tractable and chemically versatile intermediate that permitted late-stage derivatization while maintaining a consistent spatial orientation relative to the pyrazole core. The synthetic route was designed to be convergent and modular, allowing for the independent variation of the R1 and R2 positions. The 5-CF3 pyrazole intermediate served as a common scaffold from which sequential N-alkylation and C3 cross-coupling (or equivalent substitution chemistry) enabled the rapid generation of 343 structurally diverse analogues. This approach facilitated efficient library expansion while maintaining synthetic scalability and reproducibility across the chemical series. By varying R1 and R2, we were able to isolate the specific electronic and steric contributions of each substituent to the observed antiparasitic activity”*

5.¹³C NMR data are absent for the final test compounds. The authors should provide complete characterization for all synthesized molecules: ¹H NMR, ¹³C NMR, LC‑MS, and HR‑MS, together with the corresponding spectra. For synthetic intermediates, at minimum ¹H NMR and/or LC‑MS data should be supplied. This is essential for both structure confirmation and reproducibility.

We appreciate the reviewer’s concern regarding compound characterization. Because this work was conducted over an extended period, certain synthetic intermediates are no longer available for additional analysis. Nevertheless, we have substantially expanded the characterization data provided for the compounds, including ¹H NMR and ¹³C NMR for final test compounds and additional LC-MS, ESI-MS, and elemental analysis results when available. We believe this data provides sufficient support for compound identity and purity. This is included in the methods and supplemental data after Supplemental Figure 11.
 
6. To enable a full assessment of the target deconvolution data, the authors should provide a comprehensive list of the identified targets, i.e., 841 significant human proteins from the affinity capture, and 123 human proteins and 71 leishmanial proteins from TPP, while highlighting 26 overlapping human proteins. The list should include the quantitative metrics reported for each technique, presented as a Supplemental Table (ideally a spreadsheet).

We agree that access to the complete datasets will benefit readers. Accordingly, we have added this data to the supplement containing the full affinity-capture and thermal proteome profiling datasets, including all identified proteins and associated quantitative metrics, as an Excel spreadsheet.
 
7. Although 2D structures of all compounds are given in Supplemental Table 1 (pages 43‑52), I recommend adding a schematic that displays the core scaffold together with the R¹ and R² substituents for every entry in Table 1 (page 12). This will greatly aid readers in visualizing SAR trends.

We thank the reviewer for this helpful suggestion. To facilitate interpretation of the structure-activity relationship data, we have added a new schematic representation of the core scaffold and associated R¹ and R² substituents as **Supplemental Figure 3**.
 
8. Supplemental Tables 1 & 2 should be converted to a spreadsheet containing compound identifiers, 2D structures, SMILES notations, and activity data.

We appreciate this recommendation. To improve accessibility and facilitate future analysis by readers, we have added SMILES annotations to **Supplemental Data Excel file**.

9. What are the limitations of the current study?

We thank the reviewer for this important suggestion. We have expanded the Discussion to include a dedicated section addressing study limitations.

*“This study has several limitations. First, all experiments were conducted in vitro. Future studies evaluating compound 197 in relevant animal models of leishmaniasis will be important to determine translational potential. Additionally, while the proteomic analyses and biologic assays identify lysozyme as a mechanistically relevant target, direct evidence of protein–ligand binding has not yet been measured. Biophysical approaches will be necessary to confirm target engagement and further define the interaction between lysozyme and compound 197. Extending these studies to the other three lead analogs, as well as the parent compound AR-12, may provide additional insight into the structural determinants underlying this host-directed anti-leishmanial activity.”*

10. The main text uses plain numeric identifiers (e.g., 197) whereas the Materials and Methods and Supplementary Information use the “RTI‑” prefix (e.g., RTI‑197). Adopt a single naming convention throughout the manuscript, either the plain number 197 or the prefixed form RTI‑197, and apply it consistently.

We thank the reviewer for identifying this inconsistency. We have standardized compound nomenclature throughout the manuscript and now use the simplified numeric identifiers consistently.
 
11.Compound identifiers should be made bold (e.g., 197 or RTI‑197) throughout the manuscript.

We thank the reviewer for this suggestion. Compound identifiers have now been formatted consistently and bolded throughout the manuscript.

12. Page 10: replace “substituted benzene biphenyl derivatives” with “substituted benzenes and biphenyl derivatives”.

We thank the reviewer for identifying this wording issue. The text has been revised accordingly.
 
13. Page 11: replace “(50, 158, 197, 354–355)” with “(50, 158, 197, 354, and 355)”. Similar changes should be made throughout the manuscript.

We thank the reviewer for this suggestion. This and similar instances throughout the manuscript have been revised for clarity and consistency.
