# Supplementary material for: Synthesis and preliminary evaluation of novel compounds that demonstrate broad host-directed anti-leishmanial activity
Source: PLoS Negl Trop Dis. 2026 Jul 13;20(7):e0014520. doi: 10.1371/journal.pntd.0014520 (PMC13379085; doi:10.1371/journal.pntd.0014520)
Supplement: Peer review history

# Author comment: Author response to Decision Letter 0

**DOI:** 10.1371/journal.pntd.0014520.r001

**Transfer Alert**

This paper was transferred from another journal. As a result, its full editorial history (including decision letters, peer reviews and author responses) may not be present.

12 Nov 2025

# Peer review: Decision Letter 0

**DOI:** 10.1371/journal.pntd.0014520.r002

**Authors:** Brice Rotureau, Susan Madison-Antenucci

7 Jan 2026

-->PNTD-D-25-02046

Discovery of Host-Directed Small Molecules with Broad Anti-Leishmanial Efficacy

PLOS Neglected Tropical Diseases

Dear Dr. Ainslie,

Thank you for submitting your manuscript to PLOS Neglected Tropical Diseases. After careful consideration, we feel that it has merit but does not fully meet PLOS Neglected Tropical Diseases's publication criteria as it currently stands. Therefore, we invite you to submit a revised version of the manuscript that addresses the points raised during the review process.

Please submit your revised manuscript within by Mar 08 2026 11:59PM. If you will need more time than this to complete your revisions, please reply to this message or contact the journal office at plosntds@plos.org. When you're ready to submit your revision, log on to https://www.editorialmanager.com/pntd/ and select the 'Submissions Needing Revision' folder to locate your manuscript file.

Please include the following items when submitting your revised manuscript:

* A letter that responds to each point raised by the editor and reviewer(s). You should upload this letter as a separate file labeled 'Response to Reviewers'. This file does not need to include responses to any formatting updates and technical items listed in the 'Journal Requirements' section below.

* A marked-up copy of your manuscript that highlights changes made to the original version. You should upload this as a separate file labeled 'Revised Manuscript with Track Changes'.

* An unmarked version of your revised paper without tracked changes. You should upload this as a separate file labeled 'Manuscript'.

If you would like to make changes to your financial disclosure, competing interests statement, or data availability statement, please make these updates within the submission form at the time of resubmission. Guidelines for resubmitting your figure files are available below the reviewer comments at the end of this letter.

We look forward to receiving your revised manuscript.

Kind regards,

Brice Rotureau, PhD

Academic Editor

PLOS Neglected Tropical Diseases

Susan Madison-Antenucci

Section Editor

PLOS Neglected Tropical Diseases

Shaden Kamhawi

co-Editor-in-Chief

PLOS Neglected Tropical Diseases

orcid.org/0000-0003-4304-636XX

Paul Brindley

co-Editor-in-Chief

PLOS Neglected Tropical Diseases

orcid.org/0000-0003-1765-0002

**Journal Requirements:**

1) Please ensure that the CRediT author contributions listed for every co-author are completed accurately and in full.

At this stage, the following Authors/Authors require contributions: Elizabeth G. Gurysh, M. Shamim Hasan Zahid, Monica M. Johnson, Antonio Landavazo, Ojas A Namjoshi, Joseph W Wilson, Devika M. Varma, Ryan N. Woodring, Aaron T. Hendricksen, Joseph F. Vath, Baiyi Quan, Erica N. Pino, Michael C. Fitzgerald, Eric M. Bachelder, Bruce E. Blough, and Kristy Ainslie. Please ensure that the full contributions of each author are acknowledged in the "Add/Edit/Remove Authors" section of our submission form.

The list of CRediT author contributions may be found here: https://journals.plos.org/plosntds/s/authorship#loc-author-contributions

2) Please upload all main figures as separate Figure files in .tif or .eps format. For more information about how to convert and format your figure files please see our guidelines:

https://journals.plos.org/plosntds/s/figures

3) We notice that your supplementary Figures, Tables, and information are included in the manuscript file. Please remove them and upload them with the file type 'Supporting Information'. Please ensure that each Supporting Information file has a legend listed in the manuscript after the references list.

4) Some material included in your submission may be copyrighted. According to PLOSu2019s copyright policy, authors who use figures or other material (e.g., graphics, clipart, maps) from another author or copyright holder must demonstrate or obtain permission to publish this material under the Creative Commons Attribution 4.0 International (CC BY 4.0) License used by PLOS journals. Please closely review the details of PLOSu2019s copyright requirements here: PLOS Licenses and Copyright. If you need to request permissions from a copyright holder, you may use PLOS's Copyright Content Permission form.

Please respond directly to this email and provide any known details concerning your material's license terms and permissions required for reuse, even if you have not yet obtained copyright permissions or are unsure of your material's copyright compatibility. Once you have responded and addressed all other outstanding technical requirements, you may resubmit your manuscript within Editorial Manager.

Potential Copyright Issues:

- Figure S1. Please confirm whether you drew the images / clip-art within the figure panels by hand. If you did not draw the images, please provide (a) a link to the source of the images or icons and their license / terms of use; or (b) written permission from the copyright holder to publish the images or icons under our CC BY 4.0 license. Alternatively, you may replace the images with open source alternatives. See these open source resources you may use to replace images / clip-art:

- https://commons.wikimedia.org

- https://openclipart.org/.

5) In the online submission form, you indicated that "The datasets generated and analyzed during the current study are available from the corresponding author on reasonable request.". All PLOS journals now require all data underlying the findings described in their manuscript to be freely available to other researchers, either

1. In a public repository

2. Within the manuscript itself

3. Uploaded as supplementary information.

This policy applies to all data except where public deposition would breach compliance with the protocol approved by your research ethics board. If your data cannot be made publicly available for ethical or legal reasons (e.g., public availability would compromise patient privacy), please explain your reasons by return email and your exemption request will be escalated to the editor for approval. Your exemption request will be handled independently and will not hold up the peer review process, but will need to be resolved should your manuscript be accepted for publication. One of the Editorial team will then be in touch if there are any issues.

6) Please amend your detailed Financial Disclosure statement. This is published with the article. It must therefore be completed in full sentences and contain the exact wording you wish to be published.

- State the initials, alongside each funding source, of each author to receive each grant. For example: "This work was supported by the National Institutes of Health (####### to AM; ###### to CJ) and the National Science Foundation (###### to AM)."

- State what role the funders took in the study. If the funders had no role in your study, please state: "The funders had no role in study design, data collection and analysis, decision to publish, or preparation of the manuscript.".

If you did not receive any funding for this study, please simply state: u201cThe authors received no specific funding for this work.u201d

**Reviewers' Comments:**

Reviewer's Responses to Questions

**Summary and General Comments**

Use this section to provide overall comments, discuss strengths/weaknesses of the study, novelty, significance, general execution and scholarship. You may also include additional comments for the author, including concerns about dual publication, research ethics, or publication ethics. If requesting major revision, please articulate the new experiments that are needed.

Reviewer #1: The authors evaluated a library of AR-12-based compounds against Leishmania spp. The study is innovative and shows the potential of the candidate molecules. Above all, in the assays for target search and confirmation and mechanism of action, the results proved promising. For this peer review, this manuscript is considered promising for publication.

Reviewer #2: Overall, this manuscript addresses an interesting question on the development of host-targeting treatments. Experiments are well designed and appropriately interpreted. However, I have several concerns that must be addressed. Major concerns:

1. Fig 3, RTI-354 L. donovani treatment, why is there a 0.01 um data point when all the other DRC curves end at 0.1?

2. Why don't all the DRC plots in Fig 3 have the same number of data points? This also applies to Fig S3-S7. If data was suppressed, a clear rationale should be provided and non-suppressed data included in the SI.

3. For protein enrichment and thermal proteome profiling analyses: is the p-value corrected for multiple hypothesis testing (eg FDR correction or similar)? If yes, correction method should be specified in the methods, and text and figure legends should clarify that the p-values are corrected. Using uncorrected p-values is not appropriate.

4. Page 16, interpretation of Figure S8: the modified and unmodified compounds do not actually show the same curves. The lack of significant differences could be because of the big error bars. Additional replicates with less technical variation should be performed to confirm whether they perform the same or not.

Minor concerns:

1. "Further the chemical ligation to 197" (top of page 16): what does this mean? I think there are some words missing here.

2. Need LC-MS parameters for affinity capture.

3. In TPP methods, AGC target should be 4x10^5 (power or superscript) not 4x105 (bottom of page 33, two instances).

Reviewer #3: * This study tackles a significant unmet need in leishmaniasis therapy and proposes a well‑defined translational pathway toward novel host‑directed treatments. Nevertheless, the additional experiments listed above are essential before the manuscript can be recommended for publication.

**********

PLOS authors have the option to publish the peer review history of their article (what does this mean?). If published, this will include your full peer review and any attached files.

If you choose “no”, your identity will remain anonymous but your review may still be made public.

**Do you want your identity to be public for this peer review?**  For information about this choice, including consent withdrawal, please see our Privacy Policy.

Reviewer #1: No

Reviewer #2: No

Reviewer #3: No

**********

**Key Review Criteria Required for Acceptance?**

As you describe the new analyses required for acceptance, please consider the following:

**Methods**

-Are the objectives of the study clearly articulated with a clear testable hypothesis stated?

-Is the study design appropriate to address the stated objectives?

-Is the population clearly described and appropriate for the hypothesis being tested?

-Is the sample size sufficient to ensure adequate power to address the hypothesis being tested?

-Were correct statistical analysis used to support conclusions?

-Are there concerns about ethical or regulatory requirements being met?

Reviewer #3: * Although the proteomic data suggest that compound 197 interacts with lysozyme, no direct evidence of protein‑ligand binding has been presented. To substantiate this mechanism, the authors should perform at least one biophysical assay (e.g., DSF, ITC, SPR, or FRET) using recombinant lysozyme together with the four most promising hits and the parent compound AR‑12.

* The five aforementioned compounds should also be evaluated for their ability to modulate lysozyme enzymatic activity (e.g., using a muramidase assay).

* Moreover, the best four hits should be evaluated against recombinant PDK-1 (e.g., using a kinase assay), the primary target of the parent compound AR-12 (IC₅₀ = 5 µM; https://doi.org/10.1158/0008-5472.CAN-03-4063).

**********

**Results**

-Does the analysis presented match the analysis plan?

-Are the results clearly and completely presented?

-Are the figures (Tables, Images) of sufficient quality for clarity?

Reviewer #3: * The chemical synthesis of the AR‑12 analog library is central to the study, yet the manuscript provides only a brief overview. A detailed description of the design strategy and synthetic routes should be incorporated into the Results and Discussion section.

* ¹³C NMR data are absent for the final test compounds. The authors should provide complete characterization for all synthesized molecules: ¹H NMR, ¹³C NMR, LC‑MS, and HR‑MS, together with the corresponding spectra. For synthetic intermediates, at minimum ¹H NMR and/or LC‑MS data should be supplied. This is essential for both structure confirmation and reproducibility.

* To enable a full assessment of the target deconvolution data, the authors should provide a comprehensive list of the identified targets, i.e., 841 significant human proteins from the affinity capture, and 123 human proteins and 71 leishmanial proteins from TPP, while highlighting 26 overlapping human proteins. The list should include the quantitative metrics reported for each technique, presented as a Supplemental Table (ideally a spreadsheet).

* Although 2D structures of all compounds are given in Supplemental Table 1 (pages 43‑52), I recommend adding a schematic that displays the core scaffold together with the R¹ and R² substituents for every entry in Table 1 (page 12). This will greatly aid readers in visualizing SAR trends.

* Supplemental Tables 1 & 2 should be converted to a spreadsheet containing compound identifiers, 2D structures, SMILES notations, and activity data.

**********

**Conclusions**

-Are the conclusions supported by the data presented?

-Are the limitations of analysis clearly described?

-Do the authors discuss how these data can be helpful to advance our understanding of the topic under study?

-Is public health relevance addressed?

Reviewer #3: * What are the limitations of the current study?

**********

**Editorial and Data Presentation Modifications?**

Use this section for editorial suggestions as well as relatively minor modifications of existing data that would enhance clarity. If the only modifications needed are minor and/or editorial, you may wish to recommend “Minor Revision” or “Accept”.

Reviewer #3: * The main text uses plain numeric identifiers (e.g., 197) whereas the Materials and Methods and Supplementary Information use the “RTI‑” prefix (e.g., RTI‑197). Adopt a single naming convention throughout the manuscript, either the plain number 197 or the prefixed form RTI‑197, and apply it consistently.

* Compound identifiers should be made bold (e.g., 197 or RTI‑197) throughout the manuscript.

* Page 10: replace “substituted benzene biphenyl derivatives” with “substituted benzenes and biphenyl derivatives”.

* Page 11: replace “(50, 158, 197, 354–355)” with “(50, 158, 197, 354, and 355)”. Similar changes should be made throughout the manuscript.

[NOTE: If reviewer comments were submitted as an attachment file, they will be attached to this email and accessible via the submission site. Please log into your account, locate the manuscript record, and check for the action link "View Attachments". If this link does not appear, there are no attachment files.]

**Figure resubmission:**

While revising your submission, we strongly recommend that you use PLOS’s NAAS tool (https://ngplosjournals.pagemajik.ai/artanalysis) to test your figure files. NAAS can convert your figure files to the TIFF file type and meet basic requirements (such as print size, resolution), or provide you with a report on issues that do not meet our requirements and that NAAS cannot fix.

After uploading your figures to PLOS’s NAAS tool - https://ngplosjournals.pagemajik.ai/artanalysis, NAAS will process the files provided and display the results in the "Uploaded Files" section of the page as the processing is complete. If the uploaded figures meet our requirements (or NAAS is able to fix the files to meet our requirements), the figure will be marked as "fixed" above. If NAAS is unable to fix the files, a red "failed" label will appear above. When NAAS has confirmed that the figure files meet our requirements, please download the file via the download option, and include these NAAS processed figure files when submitting your revised manuscript.

**Reproducibility:**

To enhance the reproducibility of your results, we recommend that authors of applicable studies deposit laboratory protocols in protocols.io, where a protocol can be assigned its own identifier (DOI) such that it can be cited independently in the future. Additionally, PLOS ONE offers an option to publish peer-reviewed clinical study protocols. Read more information on sharing protocols at https://plos.org/protocols?utm_medium=editorial-email&utm_source=authorletters&utm_campaign=protocols

-->

# Author comment: Author response to Decision Letter 1

**DOI:** 10.1371/journal.pntd.0014520.r003

24 Jun 2026

Attachment Submitted filename: Response to Reviewers.docx

# Editor report: Decision Letter 1

**DOI:** 10.1371/journal.pntd.0014520.r004

**Authors:** Brice Rotureau, Susan Madison-Antenucci, Brice Rotureau, Susan Madison-Antenucci

30 Jun 2026

Dear Professor Ainslie,

We are pleased to inform you that your manuscript 'Synthesis and preliminary evaluation of novel compounds that demonstrate broad host-directed anti-leishmanial activity' has been provisionally accepted for publication in PLOS Neglected Tropical Diseases.

Before your manuscript can be formally accepted you will need to complete some formatting changes, which you will receive in a follow up email. A member of our team will be in touch with a set of requests.

Please note that your manuscript will not be scheduled for publication until you have made the required changes, so a swift response is appreciated.

IMPORTANT: The editorial review process is now complete. PLOS will only permit corrections to spelling, formatting or significant scientific errors from this point onwards. Requests for major changes, or any which affect the scientific understanding of your work, will cause delays to the publication date of your manuscript.

Should you, your institution's press office or the journal office choose to press release your paper, you will automatically be opted out of early publication. We ask that you notify us now if you or your institution is planning to press release the article. All press must be co-ordinated with PLOS.

Thank you again for supporting Open Access publishing; we are looking forward to publishing your work in PLOS Neglected Tropical Diseases.

Best regards,

Brice Rotureau, PhD

Academic Editor

PLOS Neglected Tropical Diseases

Susan Madison-Antenucci

Section Editor

PLOS Neglected Tropical Diseases

Shaden Kamhawi

co-Editor-in-Chief

PLOS Neglected Tropical Diseases

orcid.org/0000-0003-4304-636XX

Paul Brindley

co-Editor-in-Chief

PLOS Neglected Tropical Diseases

orcid.org/0000-0003-1765-0002

***********************************************************

Thank you for this revised version that was significantly improved by considering most of the reviewers' comments.

I would however recommend adding elements of your response to the questions 1 and 3 from reviewer 3 in the discussion.

p.p1 {margin: 0.0px 0.0px 0.0px 0.0px; line-height: 16.0px; font: 14.0px Arial; color: #323333; -webkit-text-stroke: #323333}span.s1 {font-kerning: none

# Editor report: Acceptance letter

**DOI:** 10.1371/journal.pntd.0014520.r005

**Authors:** Brice Rotureau, Susan Madison-Antenucci, Brice Rotureau, Susan Madison-Antenucci

Dear Professor Ainslie,

We are delighted to inform you that your manuscript, "Synthesis and preliminary evaluation of novel compounds that demonstrate broad host-directed anti-leishmanial activity," has been formally accepted for publication in PLOS Neglected Tropical Diseases.

We have now passed your article onto the PLOS Production Department who will complete the rest of the publication process. All authors will receive a confirmation email upon publication.

The corresponding author will soon be receiving a typeset proof for review, to ensure errors have not been introduced during production. Please review the PDF proof of your manuscript carefully, as this is the last chance to correct any scientific or type-setting errors. Please note that major changes, or those which affect the scientific understanding of the work, will likely cause delays to the publication date of your manuscript. Note: Proofs for Front Matter articles (Editorial, Viewpoint, Symposium, Review, etc...) are generated on a different schedule and may not be made available as quickly.

Soon after your final files are uploaded, the early version of your manuscript will be published online unless you opted out of this process. The date of the early version will be your article's publication date. The final article will be published to the same URL, and all versions of the paper will be accessible to readers.

For Research Articles, you will receive an invoice from PLOS for your publication fee after your manuscript has reached the completed accept phase. If you receive an email requesting payment before acceptance or for any other service, this may be a phishing scheme. Learn how to identify phishing emails and protect your accounts at https://explore.plos.org/phishing.

Thank you again for supporting open-access publishing; we are looking forward to publishing your work in PLOS Neglected Tropical Diseases.

Best regards,

Shaden Kamhawi

co-Editor-in-Chief

PLOS Neglected Tropical Diseases

Paul Brindley

co-Editor-in-Chief

PLOS Neglected Tropical Diseases
